# Genome-wide characterization and expression analyses of superoxide dismutase (*SOD*) genes in *Gossypium hirsutum*

**DOI:** 10.1186/s12864-017-3768-5

**Published:** 2017-05-12

**Authors:** Wei Wang, Xiaopei Zhang, Fenni Deng, Rui Yuan, Fafu Shen

**Affiliations:** State Key Laboratory of Crop Biology, College of Agronomy, Shandong Agricultural University, Tai’an, 271018 Shandong People’s Republic of China

**Keywords:** SOD, Upland cotton, Genome wide analysis, Abiotic stress, Expression profiles

## Abstract

**Background:**

Superoxide dismutases (SODs) are a key antioxidant enzyme family, which have been implicated in protecting plants against the toxic effects of reactive oxygen species. Despite current studies have shown that the gene family are involved in plant growth and developmental processes and biotic and abiotic stress responses, little is known about its functional role in upland cotton.

**Results:**

In the present study, we comprehensively analyzed the characteristics of the *SOD* gene family in upland cotton (*Gossypium hirsutum*). Based on their conserved motifs, 18 *GhSOD* genes were identified and phylogenetically classified into five subgroups which corroborated their classifications based on gene-structure patterns and subcellular localizations. The *GhSOD* sequences were distributed at different densities across 12 of the 26 chromosomes. The conserved domains, gene family evolution *cis*-acting elements of promoter regions and miRNA-mediated posttranscriptional regulation were predicted and analyzed. In addition, the expression pattern of 18 *GhSOD* genes were tested in different tissues/organs and developmental stages, and different abiotic stresses and abscisic acid, which indicated that the *SOD* gene family possessed temporal and spatial specificity expression specificity and may play important roles in reactive oxygen species scavenging caused by various stresses in upland cotton.

**Conclusions:**

This study describes the first genome-wide analysis of the upland cotton *SOD* gene family, and the results will help establish a foundation for the further cloning and functional verification of the *GhSOD* gene family during stress responses, leading to crop improvement.

**Electronic supplementary material:**

The online version of this article (doi:10.1186/s12864-017-3768-5) contains supplementary material, which is available to authorized users.

## Background

Allotetraploid upland cotton (*Gossypium hirsutum*) accounts for more than 90% of cultivated cotton worldwide and is an important economic crop that provides fiber, seed oil, and protein meal [1]. However, its growth and yield are affected by various environmental stressors, including salinity, drought, heat, cold, herbicides, heavy metals and pathogens. The most common result of such stress is the generation of toxic reactive oxygen species (ROS). Excess ROS, such as superoxide anion, hydroxyl radical, hydrogen peroxide and singlet oxygen, could result in membrane damage, protein oxidation and DNA lesions, and could even lead to irreparable metabolic dysfunctions and cell death. To cope with ROS toxicity, plants have developed efficient antioxidative mechanisms, including many non-enzymatic and enzymatic defense systems. Among the enzymatic defense systems, superoxide dismutases (SODs) (EC 1.15.1.1), a family of antioxidant enzymes, are the first line of defense against oxidative damage and are ubiquitous in every cell of all plant types. As a major defense system against oxidative stress in plants, SOD catalyzes the conversion or dismutation of toxic superoxide anion radicals to hydrogen peroxide and molecular oxygen [2].

In plants, SODs have been classified into three groups based on the type of prosthetic metal: copper/zinc (Cu/Zn)-SOD, manganese (Mn)-SOD and iron (Fe)-SOD. SOD proteins are encoded by nuclear genes and distributed to different cellular compartments. Cu/Zn-SOD is present chiefly in chloroplasts and in the cytosol and mitochondria. Mn-SOD is mainly localized in mitochondria but is also in different types of peroxisomes. Fe-SOD occurs in chloroplasts, and also in peroxisomes and mitochondria [3]. A considerable number of *SOD* genes have been cloned from various monocots and dicots [4–6], and, since the first *SOD* gene cloned from maize [7], reports have indicated that *SODs* are important for stress tolerance [8, 9]. Additionally, plants with *SOD* gene families that have been characterized at the genome-wide level include *Arabidopsis thaliana* [10], *Dimocarpus longan* [4], *Sorghum bicolor* [11], *Populus trichocarpa* [5], *Musa acuminate* [6], *Gossypium raimondii* and *Gossypium arboretum* [12], and the numbers of each of the three SOD-type genes vary among them.

In a previous study, Voloudakis et al. studied the SOD isoenzymes in upland cotton using genetic engineering methods and revealed that the three SOD isoenzymes respond to bacterial blight of cotton [13]. Additionally, Holaday et al. suggested that Mn-SOD increases the tolerance of cotton to low temperature and high light using transgenic technology [14]. However, these studies focused only on the proteins and activity changes, and were unable to effectively elucidate the exact roles of the cotton *SOD* gene family under adverse conditions. Recently, the whole-genome sequences of two diploid cottons (*G. raimondii* and *G. arboretum*) [15–17] and two allotetraploid cottons (*G. hirsutum* and *Gossypium barbadense*) [18–21] were made available to the public, facilitating molecular studies on the expression and the regulatory mechanisms of the cotton *SOD* gene family in response to various stresses. Using these genomes, a genome-wide analysis of the *SOD* gene family in two diploid cottons was performed. In this study, the identification of the *SOD* gene family in upland cotton (*G. hirsutum*) was performed to analyze genomic organization, gene structure, motif composition and phylogenetic relationships. Then, the putative promoters of the upland cotton *SOD*s were also investigated, *cis*-elements involved in stress responses were analyzed, and miRNA target sites of *GhSOD*s were predicted to further clarify the regulatory mechanisms of gene expression. Finally, we studied the expression patterns of the upland cotton *SOD* gene family under abiotic (salt, drought, cold and heat) and hormonal stresses [abscisic acid (ABA)] using a real-time quantitative PCR (qPCR) detection system. This was the first comprehensive study of the *SOD* gene family in upland cotton and provided valuable information for understanding the classification, evolution and putative functions of this family on the whole-genome scale.

## Methods

### Identification of *SOD* genes

The *G. hirsutum* genomes and annotation files [*Gossypium hirsutum* (AD1) Genome NAU-NBI Assembly v1.1 & Annotation v1.1] were downloaded from CottonGen (https://www.cottongen.org). We then filtered gene annotation results based on the following criteria [22]: (1) the longest transcript in each gene loci was chosen to represent that locus; (2) CDSs with length < 150 bp were filtered out; (3) CDSs with percentages of ambiguous nucleotides (‘N’) > 50% were filtered out; (4) CDSs with internal termination codons were filtered out; and (5) the CDSs with hits (BLAST identities ≥ 80%) to RepBase sequences were filtered out (http://www.girinst.org/repbase/index.html). To identify members of the *SOD* gene family in *G. hirsutum*, we used SOD data from previous studies in *G. raimondii* and *G. arboretum* [12] and retrieved SOD protein sequences from the NCBI (http://www.ncbi.nlm.nih.gov/protein/) and the JGI database (http://www.phytozome.net). The full-length protein sequences from *A. thaliana* (NCBI accession: NP_172360.1, NP_565666.1, NP_197311.1, NP_199923.1, NP_197722.1 and NP_187703.1) were used as query sequences to perform multiple database searches using the BLAST algorithm for Proteins (BLASTP) [23]. After removing alignments with identities < 50%, the resulting candidate SOD proteins were aligned to each other to ensure that no gene was represented multiple times. InterProScan (version 4.8) [24] was further used to confirm the inclusion of the SOD domain in each candidate sequence using the Pfam database. The *SOD* gene members of the other 17 plant genomes shown in Additional file 1 were identified using similar methods said above.

*SOD* gene data, including accession number, chromosomal location and ORF length, were collected from the *G. hirsutum* genome and annotation files. All of the candidate SOD protein sequences were analyzed using InterProScan (version 4.8) with the Pfam database [25], and conserved domains were located with Pfam HMM search (http://pfam.xfam.org/search). Conserved protein motifs were predicted by MEME Suite (http://meme-suite.org/tools/meme) with the default settings, except that the minimum and maximum motif widths were set to 20 and 150 amino acids [26]. Physico-chemical characteristics of SOD proteins, including the number of amino acids, molecular weight, theoretical isoelectric point and instability index, were calculated using the ProtParam tool (http://www.expasy.org/tools/protparam.html). Predictions of subcellular localizations of SOD proteins were performed with CELLO v.2.5 (http://cello.life.nctu.edu.tw/) [27] and WoLF PSORT servers (http://www.genscript.com/wolf-psort.html) [28].

### Phylogenetic analysis

We aligned the full-length coding sequences of plant *SOD* genes using the ClustalW program with default parameters [29]. The Gblocks_0.91b local program selected the conserved blocks from multiple alignments [30]. ModelGenerator_0.85 was used to evaluate the fit of major models of amino acid substitutions, and the Bayesian information criterion and Akaike information criterion were applied to select the fit model that met the amino acid frequencies and rates of amino acid substitutions for each amino acid pair using a discrete gamma distribution [31]. The phylogenetic tree was then constructed using the maximum likelihood method with a bootstrap analysis of 1,000 replicates and the Jones-Taylor-Thornton (JTT) with Gamma Distributed (G) substitution model using MEGA6.0 software [32].

### Chromosomal locations and syntenic analysis

The chromosomal distribution of *SOD* genes was drafted from top to bottom on upland cotton chromosomes according to gene positions in the genome annotation by Circos-0.69 (http://circos.ca/) [33]. A syntenic analysis was conducted locally using a method similar to that developed for the Plant Genome Duplication Database (http://chibba.pgml.uga.edu/duplication/) [34]. We used BLAST version 2.2.9 [35] for the pairwise comparison of the filtered SOD protein sets of *G. hirsutum*, *G. raimondii* and *G. arboreum*. Then, MCscanX [36] was employed to identify homologous regions, and syntenic blocks were evaluated using Circos-0.69 (http://circos.ca/) [33]. Default parameters were used in all of the steps. Tandem duplications were characterized as multiple genes of one family located within the same or neighboring intergenic region [37].

### Gene sequences and putative functional analysis

Exons-intron structures of *SOD* genes from *G. hirsutum* were identified using GSDS 2.0 (http://gsds.cbi.pku.edu.cn/) [38]. Functional annotations of *GhSOD* genes were analyzed using the GO term analysis tool (http://geneontology.org/) [39], based on their molecular functions, biological processes, and cellular localizations. The GO annotation results were plotted by the WEGO web tool (http://wego.genomics.org.cn/cgi-bin/wego/index.pl) [40].

### Prediction of *GhSODs* regulatory elements

We obtained cotton miRNA sequences from miRBase (http://www.mirbase.org/) [41], the Plant MicroRNA database (http://bioinformatics.cau.edu.cn/PMRD/) [42], the Cotton EST database (http://www.ncbi.nlm.nih.gov/nucest) and published articles. *GhSOD* genes targeted by miRNAs were predicted by searching 5’ and 3’ UTRs and the CDS of all *GhSOD* genes for complementary sequences of the cotton miRNAs using the psRNATarget server with default parameters (http://plantgrn.noble.org/psRNATarget/?function=3) (Additional file 2) [43]. We selected the targeted sites with high degrees of complementarity shown in Fig. 6. Transcriptional response elements of *SOD* gene promoters were predicted using the PlantCARE serve, a database of plant *cis*-acting regulatory elements and a portal to tools for in *silico* analysis of promoter sequences (http://bioinformatics.psb.ugent.be/webtools/plantcare/html/) [44].

### RNA-seq data analysis

We obtained whole-transcriptome sequencing data for *G. hirsutum* from the NCBI Sequence Read Archive (SRA) (http://www.ncbi.nlm.nih.gov/sra) to analyze the tissue/organ-specific, stage-specific and stress-induced expression patterns of cotton *SOD* genes. The details of the SRA are shown in Additional file 3. We then used fastq-dump from SRAToolkit.2.4.5-2-centos_linux64 to convert the SRA data into the fastq format (http://www.ncbi.nlm.nih.gov/Traces/sra). The HISAT2 pipeline was used to build indices and align the clean reads generated from the above steps to their reference genomes (https://ccb.jhu.edu/software/hisat2/index.shtml) [45]. Then, the text file in the SAM format, which was produced by HISAT2, was sorted and converted to the BAM format using the SAMtools program (http://samtools.sourceforge.net/) [46]. StringTie version 1.2.4 (https://ccb.jhu.edu/software/stringtie/index.shtml) [47] was used to calculate the expression level of each transcript using the reference annotation file of upland cotton in the GFF3 format, which was fragments per kilobase per million reads values. Finally, these values of the *GhSOD* candidates were extracted and plotted using MySQL and the R programming language, respectively.

### Plant materials and stress treatments

Upland cotton TM-1 (provided by State Key Laboratory of Crop Biology, Shandong Agricultural University) was employed in this study. The seeds were germinated in a soil mix [peat moss: perlite, 2:1 (v/v)] in plastic pots at 28 °C in the dark. And identical seedlings were placed in plant growth chambers at temperature regime of 28/21 °C, light intensity of 3,300 lx and photoperiod of 16 h light/8 h dark. And upland cotton TM-1 plants were also grown under standard field conditions (naturally rain-fed with a daytime high temperature of 30 °C –37 °C and nighttime low temperature of 15 °C–30 °C) in Tai’an, the experimental station of Shandong Agricultural University. For the tissue/organ-specific expression profiling analysis, cotyledons, hypocotyls and roots were harvested from 10-day-old seedlings. Young leaves (5-cm diameter), fully expanded leaves (15-cm diameter) and flowers were harvested from field-grown plants. All of the tissues/organs were frozen in liquid nitrogen and stored at −80 °C until total RNA was extracted.

For the gene differential expression profiling analysis, the plantlets were subjected to different abiotic stress treatments after the expansion of the first true leaf. The plantlets were cultivated in Hoagland’s solution supplemented with 200 mM NaCl for the salt treatment, 20% (v/v) polyethylene glycol 6000 for the drought treatment, and sprayed with 100 μM ABA in 0.02% (v/v) Tween 20 for the ABA treatment. The leaves of treated plantlets were harvested at 0, 12 and 24 h. All of the treatments occurred at 28 °C with 3,300 lx continuous light, except the cold stress, which was conducted at 4 °C in a plant growth chamber with 400 lx continuous light, and the heat stress, which was conducted at 38 °C in a plant growth chamber with 3,300 lx continuous light. All of the harvested samples were frozen in liquid nitrogen and stored at −80 °C until total RNA was extracted.

### Real-time reverse transcription-PCR and data analysis

Total RNA from these samples was isolated using RNAprep Pure Plant Kit (Polysaccharides & Polyphenolics-rich, DP441) (TIANGEN, Beijing, China). The concentrations of the isolated RNA samples were determined by 1.5% agarose gel electrophoresis and a NanoDrop 2000 Spectrophotometer (Thermo Fisher Scientific, Wilmington, DE, USA). Reverse transcription PCR was carried out using PrimeScript™ RT reagent Kit with gDNA Eraser (RR047A) (TaKaRa, Dalian, China). Transcript levels were determined using a QuantStudio™ 12 K Flex Real-Time PCR System (Applied Biosystems™, Carlsbad, CA, USA) and SYBR® *Premix Ex Taq*™ (RR420A) (TaKaRa), with three biological replicates and technical replicates. PCRs included an initial denaturation at 95 °C for 3 min, followed by 40 cycles at 95 °C for 10 s, 60 °C for 20 s, and 72 °C for 30 s in a reaction volume of 20 μL in a 96-well plate. Following the PCR, a melting curve analysis was performed. Cycle threshold was used for the relative quantification of the input target number. Relative fold difference represents the number of treated target gene transcript copies relative to the number of untreated gene transcript copies, and was calculated according to the 2^−ΔΔCT^ method [48]; The data were analyzed with Microsoft Office Excel. To normalize the variance among samples, *GhUBQ7* (NCBI accession: DQ116441) was used as an endogenous control. Gene-specific primers used for qPCR are listed in Additional file 4 and were designed using Primer Premier 5.0 [49].

## Results

### Identification of *SOD* genes in the *G. hirsutum* genome

The published genome of *G. hirsutum* (AD1) acc. ‘TM-1’ made it possible to identify all of the upland cotton *SOD* genes. The BLAST algorithm was used to search the upland cotton genome with the known *A. thaliana* SOD protein sequences as bait. A total of 18 putative *GhSOD* genes were identified, and the gene names, sequence IDs and genomic positions were shown in Table 1. Using the same method, 6 *CrSOD*s from the green alga *Chlamydomonas reinhardtii* (v5.5), six *PpSOD*s from a species of moss, *Physcomitrella patens* (v3.3), six *AmtSOD*s from *Amborella trichopoda* (v1.0) and 11 *PtSOD*s from western poplar, *Populus trichocarpa*, (v3.0) were identified. The upland cotton *SOD* gene family was expanded compared with in other plant species. The number of *SOD*s was 6 each in *Brachypodium distachyon* and *Hordeum vulgare*, 7 each in *Oryza sativa*, *Sorghum bicolor* and *Setaria italica*, 10 in *Zea mays*, 12 in *Musa acuminate*, 18 in *Triticum aestivum*, 8 in *A. thaliana*, 9 each in *G. arboretum* and *G. raimondii*, and 13 in *Glycine max* (Additional file 1) [6, 11, 12].

**Table 1:** *Gossypium hirsutum SOD* genes and proteins, and their physico-chemical and biochemical properties

Gene name	Sequence ID	Genomic position	ORF, bp	Protein physicochemical characteristics				Subcellular prediction by CELLO	Subcellular prediction by WoLF PSORT	Predicted Pfam domain
				Length, aa	MW, kDa	Isoelectric point (*pI*)	Instability index			
*GhCSD1*	Gh_A13G1450_NBI-AD1_v1.1	A13:72215416…72228650 +	484	161	16.39	5.50	15.46	Cytoplasm	Cytoplasm	CZ
*GhCSD2*	Gh_D13G1747_NBI-AD1_v1.1	D13:52235057…52236244 -	457	152	15.19	5.47	17.00	Cytoplasm	Cytoplasm	CZ
*GhCSD3*	Gh_A13G0817_NBI-AD1_v1.1	A13:39169572…39170465 -	457	152	15.11	5.92	17.07	Cytoplasm	Cytoplasm	CZ
*GhCSD4*	Gh_D13G1062_NBI-AD1_v1.1	D13:29544943…29545835 -	457	152	15.08	5.92	17.63	Cytoplasm	Cytoplasm	CZ
*GhCSD5*	Gh_A11G2096_NBI-AD1_v1.1	A11:67392336…67395193 +	460	153	15.73	6.82	11.08	Cytoplasm	Cytoplasm	CZ
*GhCSD6*	Gh_D11G2412_NBI-AD1_v1.1	D11:48123478…48125573 +	466	155	15.98	6.82	2.99	Cytoplasm	Cytoplasm	CZ
*GhCSD7*	Gh_A09G2473_NBI-AD1_v1.1	scaffold2314_A09:397…6013 -	1402	467	49.64	6.13	42.69	Chloroplast	Chloroplast	CZ, ZF
*GhCSD8*	Gh_D09G0858_NBI-AD1_v1.1	D09:33602696…33604208 -	643	214	22.51	6.49	26.61	Chloroplast	Chloroplast	CZ
*GhCSD9*	Gh_A05G0722_NBI-AD1_v1.1	A05:7521668…7524293 -	691	230	24.17	6.69	22.04	Chloroplast	Chloroplast	CZ
*GhCSD10*	Gh_D05G0857_NBI-AD1_v1.1	D05:7202468…7204946 -	565	188	19.69	6.23	25.45	Chloroplast	Chloroplast	CZ
*GhMSD1*	Gh_A10G1595_NBI-AD1_v1.1	A10:86118598…86120747 -	694	231	25.96	8.50	44.62	Mitochondrion	Mitochondrion	IMA, IMC
*GhMSD2*	Gh_D10G1852_NBI-AD1_v1.1	D10:51772442…51774601 -	694	231	26.08	8.50	45.13	Mitochondrion	Mitochondrion	IMA, IMC
*GhMSD3*	Gh_A05G2383_NBI-AD1_v1.1	A05:29443309…29446068 -	691	230	25.70	7.14	34.35	Mitochondrion	Mitochondrion	IMA, IMC
*GhMSD4*	Gh_D05G2648_NBI-AD1_v1.1	D05:27530556…27533301 -	691	230	25.68	7.14	34.16	Mitochondrion	Mitochondrion	IMA, IMC
*GhFSD1*	Gh_A07G0392_NBI-AD1_v1.1	A07:4977840…4980739 +	928	309	35.65	4.84	39.39	Chloroplast	Chloroplast	IMA, IMC
*GhFSD2*	Gh_D07G0457_NBI-AD1_v1.1	D07:4887895…4890769 +	925	308	35.52	4.85	44.24	Chloroplast	Chloroplast	IMA, IMC
*GhFSD3*	Gh_A13G0530_NBI-AD1_v1.1	A13:12258203…12260923 +	769	256	29.33	6.17	29.31	Chloroplast	Chloroplast	IMA, IMC
*GhFSD4*	Gh_D13G0600_NBI-AD1_v1.1	D13:8345288…8348000 -	769	256	29.26	5.98	30.34	Chloroplast	Chloroplast	IMA, IMC

*Gh G. hirsutum*, *CSD* Cu/Zn-SOD domain, *FSD* Fe-SOD domain, *MSD* Mn-SOD domain, *aa* amino acid, *MW* theoretical molecular weight of protein, *CZ* Cu/Zn-superoxide dismutase (SOD), *IMA* Fe/Mn-SODs, alpha-hairpin domain, *IMC* Fe/Mn-SODs, C-terminal domain, *ZF* C2H2-type Zn finger

The identified upland cotton *SOD* gene family members encoded predicted proteins, and a physico-chemical analysis showed that the lengths, molecular weights, isoelectric points and instability indices of SOD proteins were within the ranges of 152–467 amino acids, 15.11–49.64 kDa, 4.84–8.50 and 2.99–45.13, respectively (Table 1). The Cu/Zn-SODs and Fe-SODs of *G. hirsutum* were acidic, and the Mn-SODs were basic. The results were similar to the findings in *O. sativa* [50] and *S. bicolor* [11]. The instability index is a protein measurement that is used to determine whether the protein will be stable in a test tube (≤40, probably stable; > 40, probably not stable) [51]. Other than GhCSD7, GhMSD1, GhMSD2 and GhFSD2, most predicted GhSOD proteins were predicted to be stable (Table 1). The results were in accordance with the research on the single Cu/Zn-SOD in *G. hirsutum* [52] and *Brassica campestris* [53].

Cu/Zn-SODs in *G. hirsutum* were predicted to localize in the cytoplasm and chloroplasts, Mn-SODs in mitochondria and Fe-SODs in chloroplasts (Table 1). The information in the literature indicated that Cu/Zn-SODs localize in the cytoplasm, chloroplasts and peroxisomes, and Fe-SODs mainly localize in the chloroplasts, and to some extent in peroxisomes and apoplast, while Mn-SODs localize in the mitochondria [54]. This corroborated our findings. Nevertheless, all of the SOD isoforms (Cu/Zn-SOD, Mn-SOD, Fe-SOD) were nuclearly coded and, where necessary, were transported to their organellar locations by means of NH_2_-terminal targeting sequences [55].

The candidate protein sequences were analyzed, using the Pfam database, for the presence of a SOD domain (Table 1). Based on the domain analysis, Cu/Zn-SODs had a Cu/Zn SOD domain (Pfam: 00080) and Mn- and Fe-SODs both had an Fe/Mn-SOD alpha-hairpin domain (Pfam: 00081) and an Fe/Mn SOD C-terminal domain (Pfam: 02777). In addition, similar to our previous findings for *G. raimondii SOD* genes (*GrCSD4*) [12], a C2H2-type Zn finger domain (Pfam: 13912) was found downstream of *GhCSD7*. Thus, *GhCSD7* and *GhCSD8* had similar gene structures and close evolutionary relationships, but differed in open reading frame (ORF) lengths, protein lengths, molecular weights and isoelectric points. We aligned the genome sequence and mRNA sequence of *GhCSD7* (Gh_A09G2473) with *GhCSD8* (Gh_D09G0858) and 5,000 base pair (bp) downstream (D09:33604208.. 33609208) (Additional file 5), and the result preliminarily indicated that gene annotation errors when sequencing the upland cotton genome were the most likely cause.

The MEME server was used for a conserved motif analysis, which identified six conserved motifs. Motifs 1, 2 and 3 were found to be in Cu/Zn-SODs, while Motifs 4, 5 and 9 were observed in Mn-SODs and Fe-SODs. Pfam analyses revealed that Motifs 1, 2 and 3 were related to the Cu/Zn SOD domain (Pfam: 00080), which contains Cu/Zn-SOD signatures and conserved Cu^2+^- and Zn^2+^-binding sites (Additional file 6). Motifs 4, 5 and 6 were related to the Fe/Mn SOD domain (Pfam: 00081, Pfam: 02777), and Motif 6 included the conserved metal-binding domain “DVWEHAYY” of the Mn- and Fe-SODs. The sequences, locations and logos of the conserved motifs in the GhSOD proteins were shown in Additional files 6 and 7. The data from the Pfam analyses supported our results. Congruent with previous studies in other plant species, the upland cotton *SOD* gene family contained characteristic amino acids, including a series of highly conserved active site residues that play roles in the sequence-specific binding of mental ions.

### Phylogenetic analysis

**Fig. 1:**
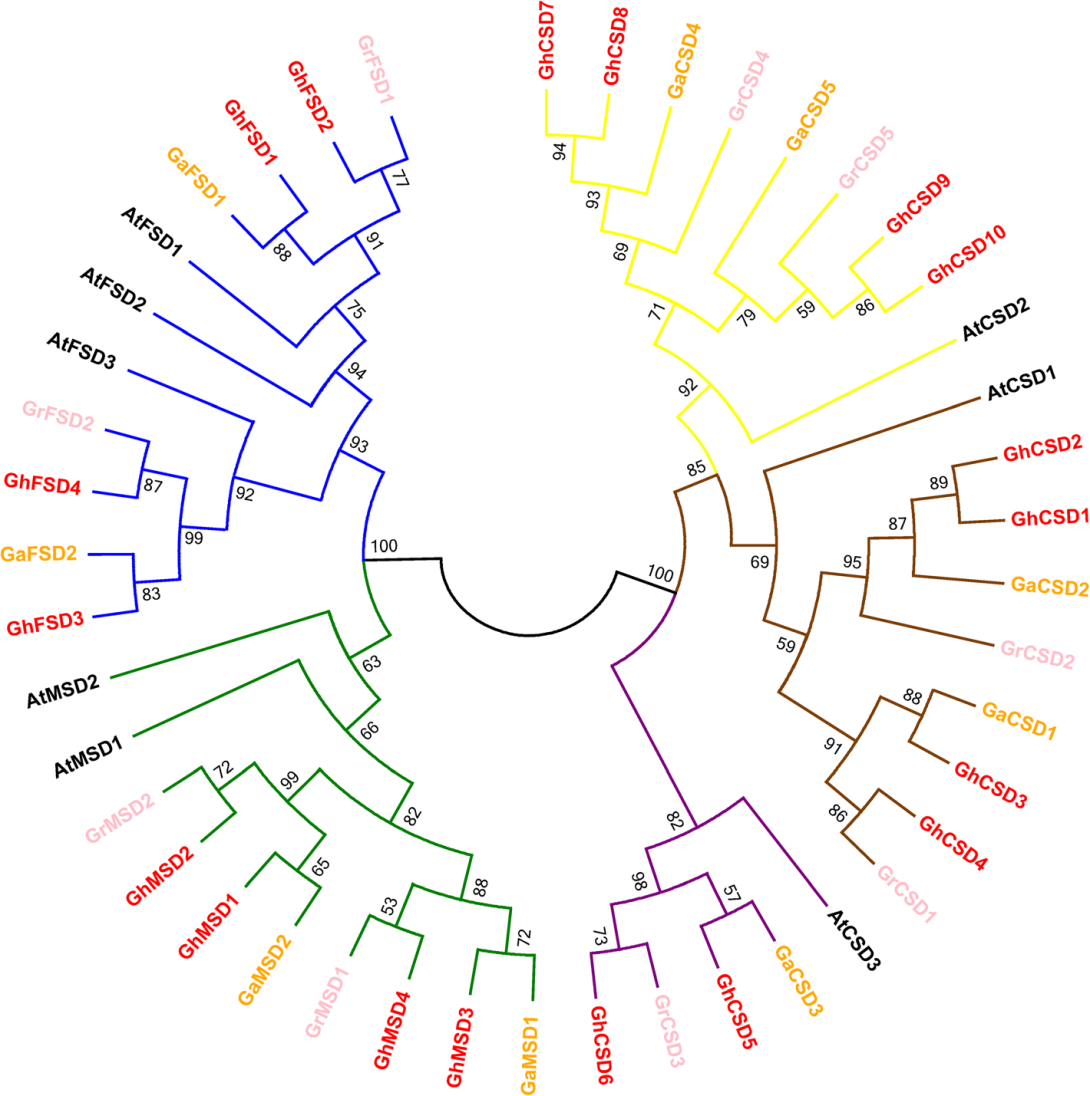
Phylogenetic tree of *SOD* genes from the genomes of three cotton species and *Arabidopsis*. A maximum-likelihood analysis was performed using the MEGA6.0 program. The Jones-Taylor-Thornton with Gamma Distributed substitution model was chosen as the most suitable substitution model based on the result of ModelGenerator before the phylogenetic tree’s construction. Red, pink, orange and black gene labels indicate *G. hirsutum*, *G. raimondii*, *G. arboretum* and *A. thaliana SOD* genes, respectively. Bootstrap values obtained from 1,000 replicates are shown below the nodes. The *SOD* genes were classified into two major groups and five subfamilies (Groups Ia, Ib, Ic, IIa and IIb, which have brown, purple, yellow, green and blue branches, respectively)

**Fig. 2:**
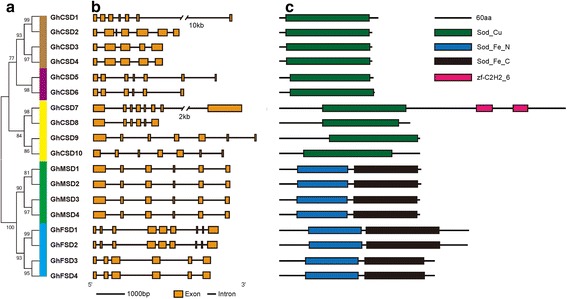
Phylogenetic tree, and gene structure and domain analyses of superoxide dismutase (SOD) in *Gossypium hirsutum*. **a** Phylogenetic tree of *G. hirsutum SOD*s constructed with MEGA 6.0 by the maximum likelihood method. Bootstrap values from 1,000 replicates are indicated at each branch. Groups Ia, Ib, Ic, IIa and IIb are *brown*, *purple*, *yellow*, *green* and *blue*, respectively. **b** Exon–intron structures of *SOD* genes. **c** Conserved domains annotated using the Pfam database

To investigate the evolutionary relationships of SODs between *Gossypium* and *A. thaliana*, we aligned multiple SOD protein sequences and constructed an unrooted phylogenetic tree for the identified multiple *SOD* genes using ClustalW and MEGA6.0, respectively (Fig. 1). The *GhSOD* genes were clustered into two major groups, Cu/Zn- and Mn/Fe-, which showed good accordance with their metal cofactor types. Group I contained three subgroups, Ia, Ib and Ic, represented by brown, purple and yellow, respectively; Group II contained two subgroups, IIa and IIb, represented by green and blue, respectively. According to the subcellular predictions (Table 1), the clustering of group Ia, Ib and Ic may be associated with the subcellular locations of the Cu/Zn-SODs. In the Cu/Zn-SODs, those localized to the chloroplasts clustered together with a high bootstrap value (92%), whereas the cytosolic Cu/Zn-SODs clustered together with a lower bootstrap value (69 and 82%). Previous research reported different structural gene characteristics between plant Cu/Zn-SODs observed in the cytoplasm, chloroplasts and peroxisomes, and those in the cytosol and chloroplasts [56]. This supported our results, which showed the separation of chloroplastic and cytosolic Cu/Zn-SODs. The separation may be affected by the *SOD* genes’ structures, including distinctive numbers and positions of exons (Fig. 2). Additionally, 4 *Gh Mn-SODs* and 4 *GhFnSOD*s formed groups IIa and IIb, respectively. This was consistent with our previous results for *GaSOD*s and *GrSOD*s in *G. arboretum* and *G. raimondii*, respectively [12].

To further confirm that there were two major SOD groups and to study the evolutionary relationships of the *GhSOD*s and *SOD*s of other plants, we selected a dataset of 70 SOD sequences from 9 flagship species, including 6 from the alga *C. reinhardtii*, 6 from the moss *P. patens*, 6 from *A. trichopoda*, 6 from *B. distachyon*, 7 from *S. bicolor*, 7 from *O. sativa*, 8 from *A. thaliana*, 13 from *G. max* and 11 from *P. trichocarpa* (Additional file 8) that were used as model organisms for studies on plant evolution, and constructed a phylogenetic tree based on their encoded amino acid sequences (Additional file 9). As shown in Additional file 2, the two major groups of the 88 SODs were well supported in this phylogenetic tree. All of the Cu/Zn-SODs formed a large clade comprising three subgroups (Groups Ia, Ib and Ic). Mn-SODs were clustered with Fe-SODs into another large clade, indicating, as previously reported [6], that these two subgroups (Groups IIa and IIb) originated from a common ancestor. The *Chlamydomonas reinhardtii* SODs clustered in an independent clade and had no Cu/Zn-SOD members. Additionally, most GhSODs showed closer relationships to SOD proteins from dicotyledonous plants than to those from monocotyledonous plants. These results indicated that land plants may be highly conserved and derived from a common ancestor that may have diverged prior to the split between bryophytes and vascular plants. It also suggested that Mn- and Fe-SODs were older than Cu/Zn-SODs, which evolved separately in bryophytes. Our results corroborated the findings of Smith and Doolittle [57].

### Gene structure analysis

To obtain further insights into the possible structural evolution of *SOD* genes in the upland cotton genome, diverse exon-intron structures of these *SOD* genes were generated by the GSDS server as shown in Fig. 2b. A gene structural analysis revealed that the ORF lengths of the *SOD* genes in *G. hirsutum* ranged from 457 to 1,402 bp (Table 1), and the numbers and positions of introns varied between four and eight (Fig. 2b), with the highest numbers of introns in *GhFSD1* and *GhFSD2*, and the lowest numbers in *GhCSD3* and *GhCSD4*. Fink and Scandalios [56] reported that all of the cytosolic and chloroplastic SODs contained seven introns, except one that had eight introns. The extra intron in the chloroplastic *SODs* corresponded in location to the second exon of the cytosolic genes. These results were not similar to our findings. In our analysis, only *GhCSD7* had seven introns among the 10 chloroplastic and cytosolic *SOD*s, and the location did not correspond to the second exon of the cytosolic genes. *Cu/Zn-SOD*s containing different intron numbers and positions showed no exon–intron structural similarities in related species [56], and the *Cu/Zn-SOD*s of *G. hirsutum* had various intron patterns (Fig. 2b). Thus, our findings were consistent with the data from previous studies. Divergences in exon–intron structures are shaped by three main mechanisms: exon/intron gain/loss, exonization/pseudoexonization and insertion/deletion [58]. Comparing the gene structure of *GhCSD1*/*GhCSD2* and *GhCSD7*/*GhCSD8*, the exon/intron gain/loss may occur in *SOD* genes during the evolution of the *G. hirsutum* genome, resulting in different intron patterns in *G. hirsutum*. Furthermore, the sizes and numbers of introns in genes varied depending on the gene and organism types, and it may be related to the functional constraint on the introns [27]. Thus, the differences in intron patterns in *G. hirsutum* could be explained by functional constraints on the introns of *SOD* genes [2]. These structural divergences may be related to an enzyme function that responds to various biotic and abiotic stress conditions with expression pattern divergences.

In addition, Mn- and Fe-SODs showed high similarities in sequences and gene structures, especially the former (Fig. 2b). Mn-SOD was the only SOD form essential for the survival of aerobic life and plants, compared with Fe-SODs and Cu/Zn-SODs. The results supported Hirsh’s hypothesis that the more important the gene/protein, the more conserved [59].

### Chromosomal locations and syntenic analysis

**Fig. 3:**
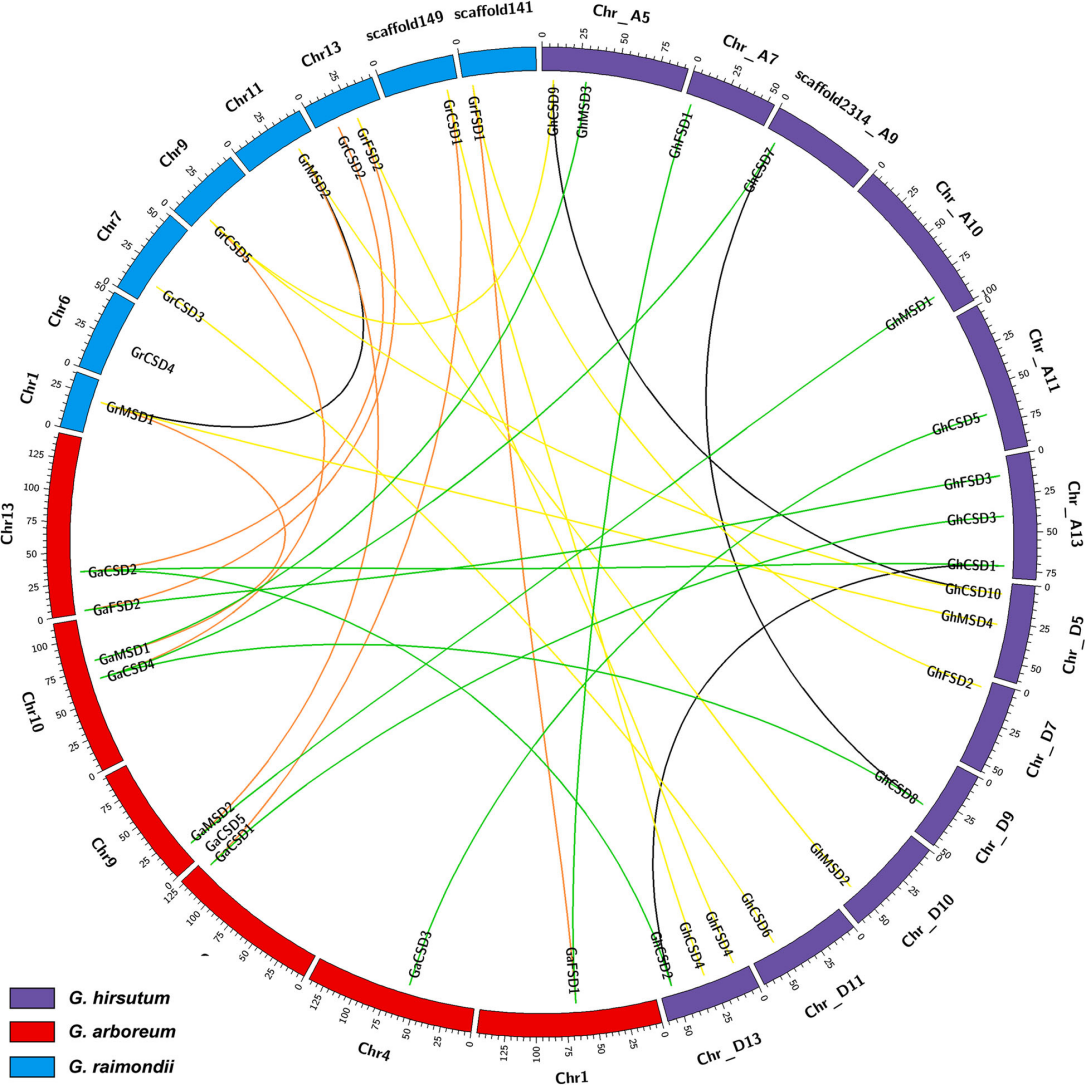
Syntenic relationships among *SOD* genes from *G. hirsutum*, *G. raimondii* and *G. arboretum. G. hirsutum*, *G. raimondii* and *G. arboretum* chromosomes are indicated in *purple*, *blue* and *red*, respectively. The putative orthologous SOD genes between *G. hirsutum* and *G. raimondii*, *G. hirsutum* and *G. arboretum*, and *G. raimondii* and *G. arboretum* are connected by *yellow*, *red* and *orange lines*, respectively. The segmental duplication genes are connected by *black lines*

The chromosomal locations of *GhSOD* genes were determined, and then a chromosomal location map was constructed (Fig. 3). Twelve out of the 26 chromosomes harbored *GhSOD* genes, with 8 (chromosomes D7, D9, D10, D11, A7, scaffold2314_A09, A10 and A11) possessing one *GhSOD* genes and 2 (chromosomes D5 and A5) possessing two *GhSOD* genes, while the others (chromosomes D13 and A13) contained three. Additionally, half of the 18 *SOD* genes were evenly distributed among the D- and A-sub-genomes.

Gene duplications, including segmental and tandem duplications, are primary driving forces in genomic evolution, and paralogous genes are the products of gene duplication events. Duplicate genes playing roles in stress response, development, signaling and transcriptional regulation tended to be retained and formed gene families that were found in almost every genome [60]. To analyze the relationships between the *SOD* genes and gene duplications, combined with our prior results, we identified the syntenic blocks of *SOD* genes among *G. hirsutum*, *G. raimondii* and *G. arboreum* (Fig. 3). An intra-genome syntenic analysis revealed that three pairs of paralogous genes (*GhCSD1* and *GhCSD2*, *GhCSD7* and *GhCSD8*, and *GhCSD9* and *GhCSD10*) were segmental duplication events and clustered together in *G. hirsutum*. They are linked together by lines in Fig. 3. Segmental duplications may have played important roles in the expansion of *SOD* genes in the upland cotton genome. The segmental duplication events may provide support for the finer regulation of SOD activities by functional divergences under various stress conditions, and for the temporal- and spatial-specific expressions of *SOD* genes [61]. Additionally, tandem duplications were not detected. The cross-genome syntenic analysis indicated, excluding *GrCSD2*, *GrCSD4* and *GaCSD5*, that another eight *GaSOD* and seven *GrSOD* genes had orthologous genes in the genome of *G. hirsutum* (Fig. 3, Additional file 10), suggesting that the other duplicate *SOD* genes, and the orthologous genes of *GrCSD2*, *GrCSD4* and *GaCSD5*, in the upland genome were lost after the *Gossypium* evolutionary event reuniting divergent cotton A and D genomes approximately 1–2 million years ago (Mya) [18]. The syntenic blocks of the *SOD* gene family between the *G. hirsutum* genome and the two diploid genomes corroborated the results in the genome sequence of allotetraploid cotton (Fig. 3) [19].

### Gene ontology (GO) annotations of *SOD*s

**Fig. 4:**
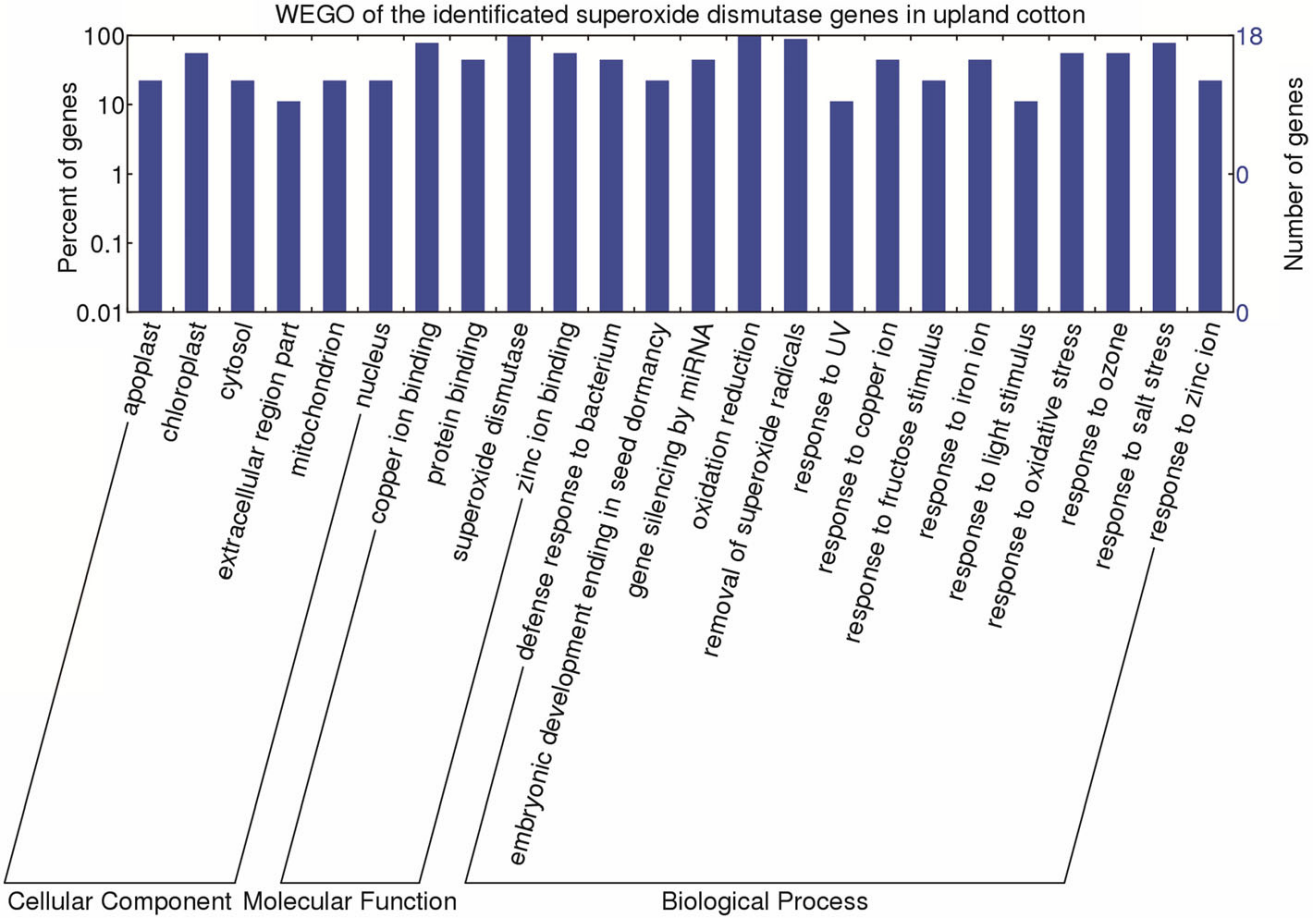
Functional classifications of the *GhSOD* genes, according to Gene Ontology Consortium predicted by considering the orthology and/or homology of *A. thaliana SOD* genes. In this ontology, ‘biological process’, ‘cellular location’ and ‘molecular function’ were treated as independent attributes

**Fig. 5:**
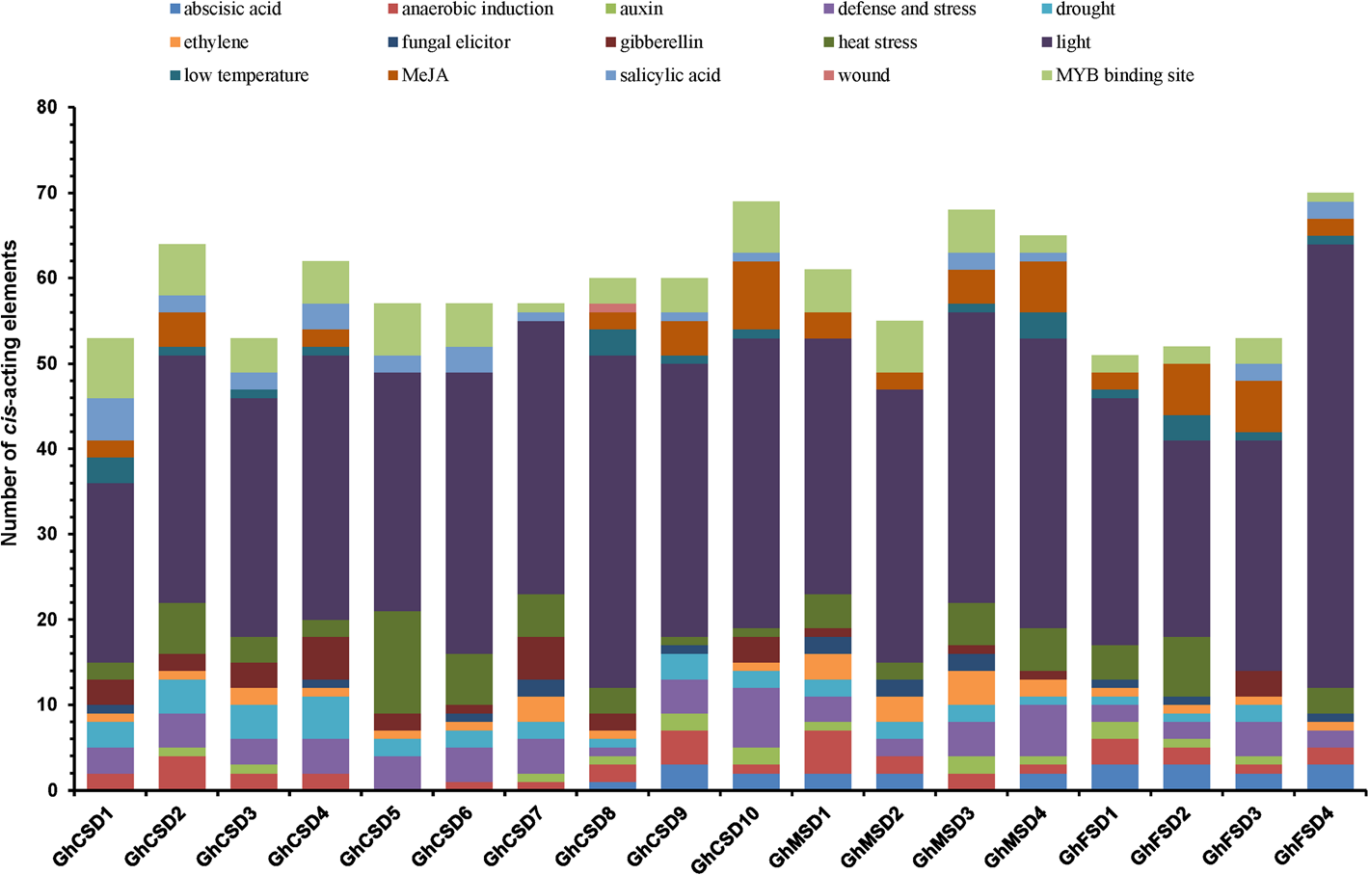
*Cis*-element analysis of putative *GhSOD* promoters related to stress responses. Different *cis*-elements with the same or similar functions are shown in the same color

‘Biological processes’ , ‘molecular functions’ , and ‘cellular components’ are characteristics of genes or gene products that enable us to understand the diverse molecular functions of proteins [39]. GO annotations of 18 upland cotton *SOD* genes were predicted by considering the orthology and/or homology of *A. thaliana SOD* genes (Fig. 4, Additional file 11). The ‘cellular components’ data were not in good agreement with the subcellular predictions of some SODs (Table 1). These results may be related to protein sequence similarities caused by genomic events. According to ‘molecular functions’ , all of the *GhSOD* genes were involved in “superoxide dismutase activity” (GO:0004784), all of the *Cu/Zn-SOD* genes had “copper ion binding” (GO:0005507) and “zinc ion binding” (GO:0008270), and all of the *Mn/Fe-SOD* genes had “metal ion binding” (GO:0046872), while the *Mn-SOD* genes belonged to the “copper ion binding” (GO:0005507) group and the *Fe-SOD* genes were in the “protein binding” (GO:0005515) group. ‘Biological processes’ annotation results indicated that all of the *GhSOD* genes contained “removal of superoxide radicals” (GO:0019430) and “oxidation-reduction process” (GO:0055114), except *GhFSD1*/*GhFSD2* only had the latter. Furthermore, the upland cotton *SOD* genes may be involved in the biological processes responding to biotic stimulus and abiotic stimulus, such as bacterium (GO:0042742), light (GO:0071484), UV-B (GO:0071493), salt (GO:0009651), ozone (GO:0010193), and sucrose metabolism process (GO:0071329). The results corroborated the putative *GhSOD* promoter analysis (Fig. 5). Particularly, the annotation demonstrated that some *GhSOD* genes’ expressions were regulated by microRNA (miRNA) at the posttranscriptional level (GO:0035195). There were some investigations revealed that miR398 targeted and regulated the expression of plant *SOD* genes in response to biotic stresses, such as salt, drought, heat, high light, ABA, methylation, paraquat and heavy metals [62–64]. The regulation of cotton *SOD* genes’ expression by miRNA needs to be studied further. In addition, *GhSOD* genes may be involved in reproductive developmental process, such as the embryonic development ending in seed dormancy (GO:0009793).

### Bioinformatics analysis of putative *GhSOD* promoters

To further understand and determine the regulatory roles of *GhSOD*s under various stresses, we gathered the *GhSOD* promoters coupled with 3-kb upstream regions of the ATG start codons from the *G. hirsutum* genome data downloaded from CottonGen and predicted the transcriptional response elements of the *GhSOD*s’ promoters using the PlantCARE tool. All 18 putative *GhSOD* promoters possessed typical TATA and CAAT boxes, which were the core *cis*-acting elements in promoter and enhancer regions. Potential regulatory *cis*-acting elements that were related to stress responses and transcription factor (TF)-binding sites are shown in Fig. 5.

As shown in Fig. 5, 11 kinds of hormone-responsive regulatory elements, ABRE, AuxRR-core, TGA-box, TGA-element, ERE, GARE-motif, TATC-box, P-box, CGTAC-motif, TGACG-motif and TCA-element which were associated with ABA, auxin (IAA), ethylene, gibberellin (GA), methyl jasmonate (MeJA) and salicylic acid (SA) responses, respectively, were found in the *GhSOD* promoters. And, seven types of stress-responsive regulatory elements, ARE, MBS, Box-W1, HSE, LTR, WUN-motif and TC-rich repeats, with responses to anaerobic induction, drought inducibility, fungal elicitors, heat stress, cold stress, wound stress, and defense and stress, respectively, were identified in the *GhSOD* promoter regions. In addition, many light-responsive elements existed in all of the *GhSOD* promoters. There were 29 different types of light-responsive elements present in the 18 upland cotton *SOD* promoters, and every promoter possessed 4 to 11 types. Increased expression levels of both Cu/Zn-SOD and Fe-SOD transcripts exist in *Arabidopsis*, tobacco and rice when exposed to light stress [65]. The constitutive overexpression of the *Cu/Zn-SOD* gene from pea in tobacco plants conferred a greater resistance to high light levels [66]. Therefore, we proposed that *GhSOD*s may be differentially regulated when subjected to light.

Moreover, all 18 *GhSOD* promoters possessed myeloblastosis (MYB)-binding sites, including CCAAT-boxes (Fig. 5). MYB-type TFs are widely distributed in all eukaryotic organisms and play important roles in regulating plant biological processes [67]. MYB is widely involved in mediating hormone signaling, cell growth, cell cycle control, primary and secondary metabolism, and cellular morphogenesis in plants [68], and in regulating plant responses to diverse environmental stimuli [69, 70]. Different types and numbers of regulatory elements were present in the distinct *GhSOD* promoters, indicating that *GhSOD* genes should be involved in cotton fiber development and have different regulatory mechanisms in response to various stress and hormone treatments.

### Predicting miRNA target sites

**Fig. 6:**
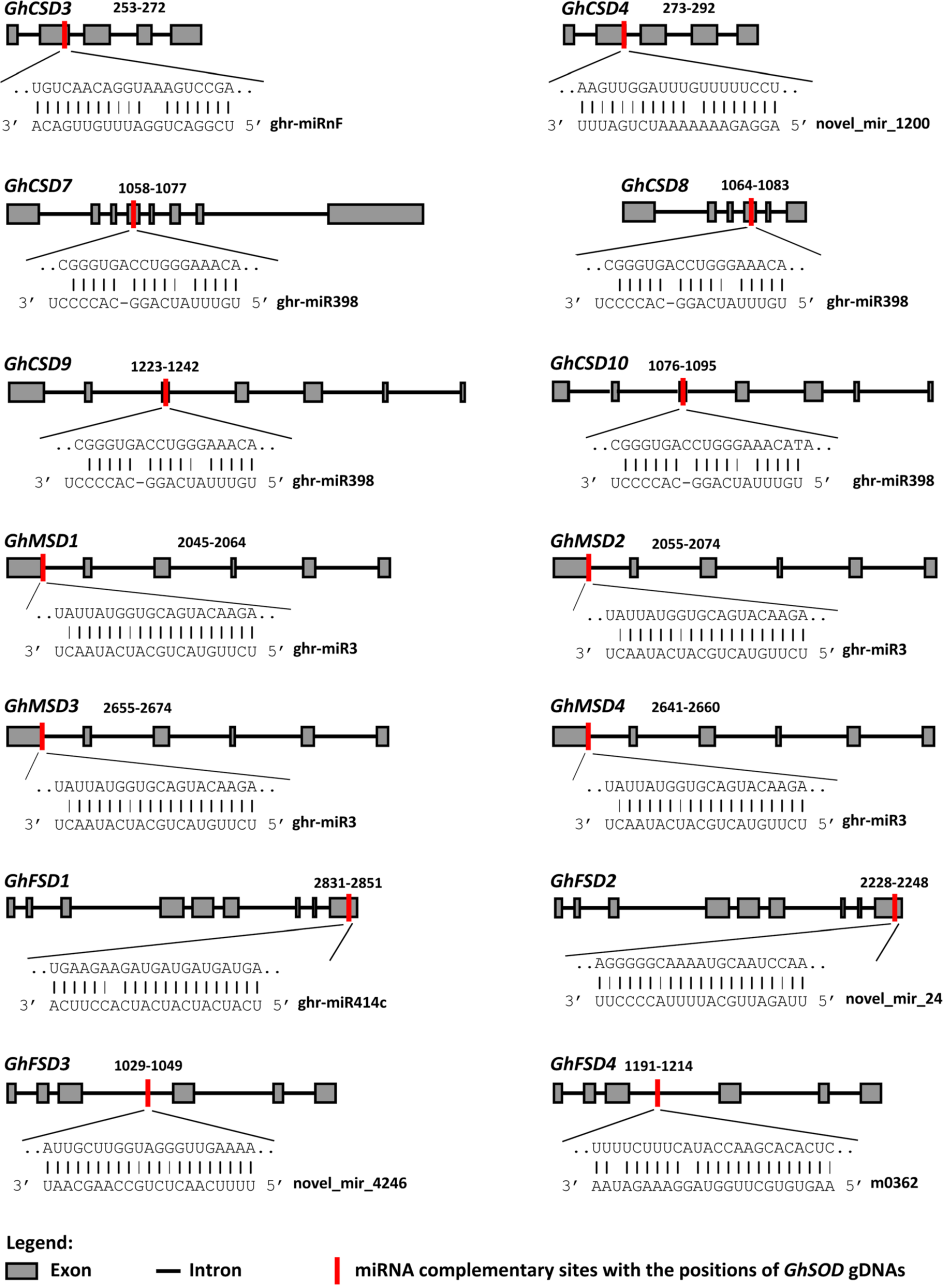
*GhSOD*s targeted by miRNAs of *Gossypium hirsutum*. The *black line* represents intron, the *rectangle filled grey* represents exon, and miRNA complementary sites (*red*) and the nucleotide positions of *GhSOD* gDNAs are shown. The RNA sequence of each complementary site from 5’ to 3’ and the predicted miRNA sequence from 3’ to 5’ are shown in the expanded regions

miR398 targeted two of three *Cu/Zn-SOD*s of Arabidopsis (*CSD1* and *CSD2*) by triggering the cleavage, or inhibiting translation, of their mRNAs [71]. To determine the miRNA-mediated posttranscriptional regulation of *GhSOD*s, we searched the 5’ and 3’ untranslated regions (UTRs), and the coding regions, of the *GhSOD*s for target sites of *G. hirsutum* miRNAs obtained from various databases and published articles on the psRNATarget server using default parameters. We obtained 20 miRNAs of *G. hirsutum* that targeted 14 *GhSOD*s at 33 prediction sites. Ghr-miR398 consistently targeted four *GhSOD* genes (*GhCSD7*, *GhCSD8*, *GhCSD9* and *GhCSD10*), and all of the targeted sites located in the coding (CDS) regions. In addition to the *Cu/Zn-SOD*s targeted by ghr-miR398, other miRNAs of upland cotton targeted *GhSOD*s. *GhCSD3* and *GhCSD4* were targeted by ghr-miRnF and/or novel_mir_1200 with sites in the second exon; *GhCSD7* and *GhCSD8* were targeted by m0166 and novel_mir_2733, respectively; *GhFSD1* and *GhFSD2* were targeted by nine and seven miRNAs with continuously distributed sites, respectively; m0362 and novel_mir_4246 targeted *GhFSD3* and *GhFSD4*, respectively, with sites in the conserved SOD domain; and ghr-miR3 targeted all four *Mn-SOD* genes in upland cotton (Fig. 6, Additional file 2). miRNA-mediated posttranscriptional regulation of *SODs* may possibly be conserved in *G. hirsutum*. These miRNAs resulted from computational predictions and deep sequencing, and they are involved in some biological processes reported in plants, including responses to environmental stresses [63, 72, 73] and regulating cell growth, development and metabolism in association with cotton fiber development [74, 75]. The expression profiles of these miRNAs and their targets needed to detect and verify in further experiments to determine their biological functions in upland cotton.

### Tissue/organ-specific and stage-specific expression profiles of *GhSODs*

**Fig. 7:**
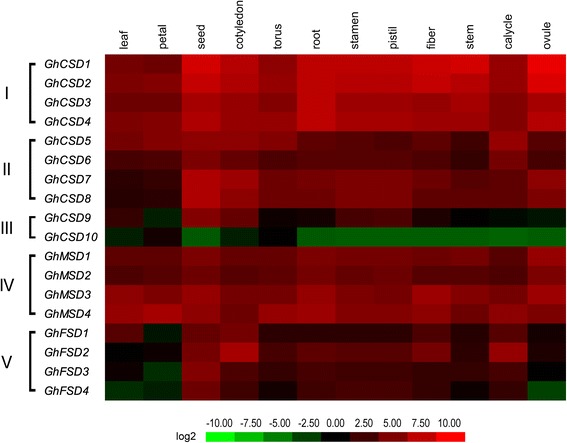
Tissue/organ-specific expression profiles of *GhSOD*s. Expressions of 18 *GhSOD*s in leaf, petal, seed, cotyledon, torus, root, stamen, pistil, fiber, calycle, stem and ovule were tested. The expression levels of the *GhSODs* were divided into five groups (I-V). The fragments per kilobase per million reads (FPKMs) calculated by RNA-seq data are shown as a heat map. The colors vary from *green* to *red*, representing the scale of the relative expression level

A strong link between gene expression and function has been suggested. The *SOD* gene family is primarily involved in plant growth, development and stress responses. To determine the biological functions in upland cotton, the expression profiles of the 18 *GhSOD* genes were analyzed in 12 tissues (leaf, petal, seed, cotyledon, torus, root, stamen, pistil, fiber, calycle, stem and ovule) using RNA-seq data recently published by Zhang et al. using the *G. hirsutum* ‘TM-1’ plant (Fig. 7) [19]. Not all of the predicted genes in the upland cotton *SOD* family were expressed in plants grown under normal conditions. Among the 18 candidate genes, 17 *GhSOD*s showed detectable expression levels in at least one of the 12 tissues. Additionally, the results enabled the classification of the upland cotton *SOD* gene family into different groups (I-V) (Fig. 7). The first group was expressed at high levels in almost all of the tested tissues and included *GhCSD1*, *GhCSD2*, *GhCSD3* and *GhCSD4*. The second group was expressed at a slightly lower level compared with the first group and included *GhSOD5*, *GhCSD6*, *GhCSD7* and *GhCSD8*. The third group, including *GhCSD9* and *GhCSD10*, had extremely low expression levels, which were almost zero in almost all of the tissues, except *GhCSD9* in seed. This suggested that *GhCSD10* may be either a pseudogene, or it was expressed at specific developmental stages or under special conditions. The fourth group had higher expression levels than the fifth and second groups and had lower expression levels than the first group, and included *GhMSD1*, *GhMSD2*, *GhMSD3* and *GhMSD4*. The fifth group had higher expression levels compared with *GhCSD9* and *GhCSD10*, and included *GhFSD1*, *GhFSD2*, *GhFSD3* and *GhFSD4*. Most of the 18 *GhSOD* genes showed higher expressions in seed and cotyledon but showed lower expressions in leaf, petal and stem, which indicated that the expression of *GhSOD* genes may be stage-specific or induced under special conditions.

**Fig. 8:**
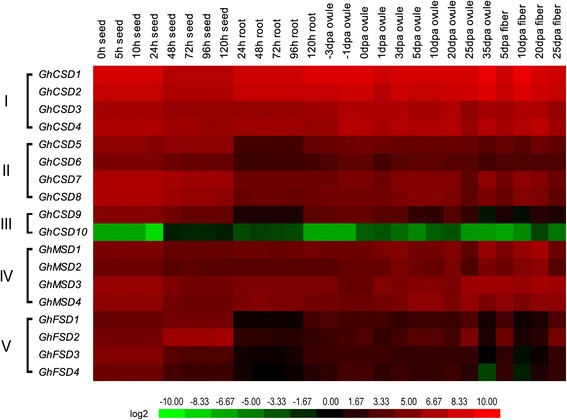
Stage-specific expression profiles of *GhSOD*s. Expressions of 18 *GhSOD*s in seed, cotyledon, root, ovule and fiber during different developmental stages. The expression levels of the *GhSODs* were divided into five groups (I-V). The FPKMs calculated by RNA-seq data are shown as a heat map. dpa, day post-anthesis. The colors vary from green to red, representing the scale of the relative expression level

To identify a stage-specific pattern for *GhSOD* expression, we used the RNA-seq data from seeds, roots, ovules and fibers at different development stages. As shown in Fig. 8, the *SOD* gene family was expressed at different development stages. Overall, the expression levels of the *GhSOD*s were divided into five groups (I-V), which was consistent with the different tissue/organ tests (Fig. 7). Among the 27 development stages of the 4 tissues/organs, *GhCSD1* and *GhCSD2* were expressed at a constitutively high level, indicating that these two genes are likely involved in basal metabolic or housekeeping functions in the seed, root, ovule and fiber development of upland cotton, and, remarkably, *GhCSD10* was expressed at an extremely low level of almost zero. These results were consistent with the corresponding results shown in Fig. 7. Fiber development has four overlapping stages (initiation, elongation, secondary cell wall biosynthesis and maturation), which were defined on the basis of the number of days post-anthesis [76]. We especially investigated the expression profiles of *GhSOD*s at different fiber development stages. The expression levels of *GhCSD1* and *GhCSD2* were always high, suggesting they were involved in fiber development. The expression levels of *GhCSD3* and *GhCSD4* were high during 10–20 and 35 days post-anthesis ovules and fibers, respectively, indicating that they participate in cell elongation, secondary cell wall biosynthesis and fiber maturation. The *GhSOD* gene family exhibited relatively high expression levels during seed development, except *GhCSD10*. These results indicated that the *SOD* gene family plays an important role in the developmental stages of upland cotton fiber.

**Fig. 9:**
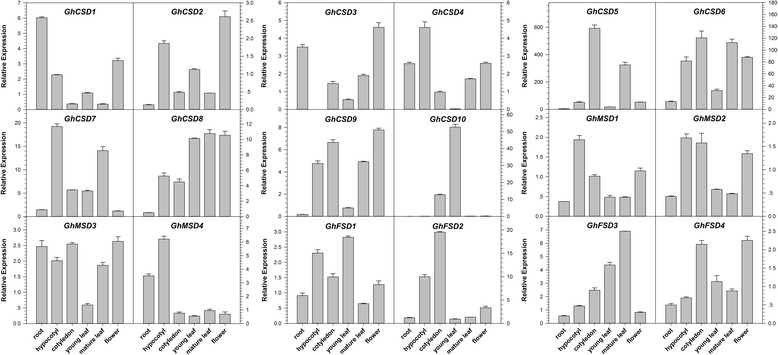
Relative transcriptional expression levels of *GhSOD*s in different tissues of *Gossypium hirsutum* ‘TM-1’ by qPCR. Different tissues are shown on the *x*-axis, and the relative expression levels on the *y*-axis. The absolute transcript levels of the respective genes in the stem were used as references and set to a value of 1

A qPCR analysis was performed to test and verify the expression patterns of *GhSOD* genes in different tissues or organs of ‘TM-1’ (Fig. 9). We used the absolute transcript levels of the genes in the stem as references and set the reference value to 1. According to the results, all of the 18 *SOD* genes in the upland cotton genome were differentially expressed in all of the tested tissues (root, hypocotyl, cotyledon, young leaf, mature leaf and flower) in non-stressed ‘TM-1’ plants. Additionally, many *GhSOD* genes were highly expressed in the cotyledon and mature leaf, followed by in the flower. Cotyledon and mature leaf are the main organs for photosynthesis at the corresponding developmental stages. The photosynthetic electron transport chain, which functions throughout photosynthesis, operates in an aerobic environment. Thus, the high expression level of the upland cotton *SOD* gene family in cotyledons and mature leaves were related to its active photosynthesis, producing a large amount of ROS. Compared with other tested tissues or organs, *GhCSD10* possessed relatively high expression levels in cotyledons and young leaf tissues, but the levels were lower than those of other genes, which corroborated the results shown in Fig. 7. We also detected the different expression levels of homologous *SOD* gene pairs located on the A- and D-subgenomes of upland cotton. Each homologous *GhSOD* gene pair showed complementary expression patterns in the same tissue or organ, whereas *GhMSD1* and *GhMSD2* showed almost the same expression levels. For instance, *GhFSD1* was expressed strongly in hypocotyl and young leaf, and weakly in root, while *GhFSD2* was expressed weakly in hypocotyl and young leaf, and strongly in root. The complementary expression patterns of the homologous *GhSOD* gene pairs indicated that the expression of one gene in the pair was enough to maintain normal physical activity in a non-stressed environment, while the other may be involved in responses to various stresses, together with other genes, or plays important roles in some other growth and development processes. Thus, the *SOD* gene family members possessing temporal- and spatial-expression specificity may be involved in the growth and development of different tissues or organs of *G. hirsutum* ‘TM-1’ plants.

### Stress-induced expression profiles of *GhSOD*s

**Fig. 10:**
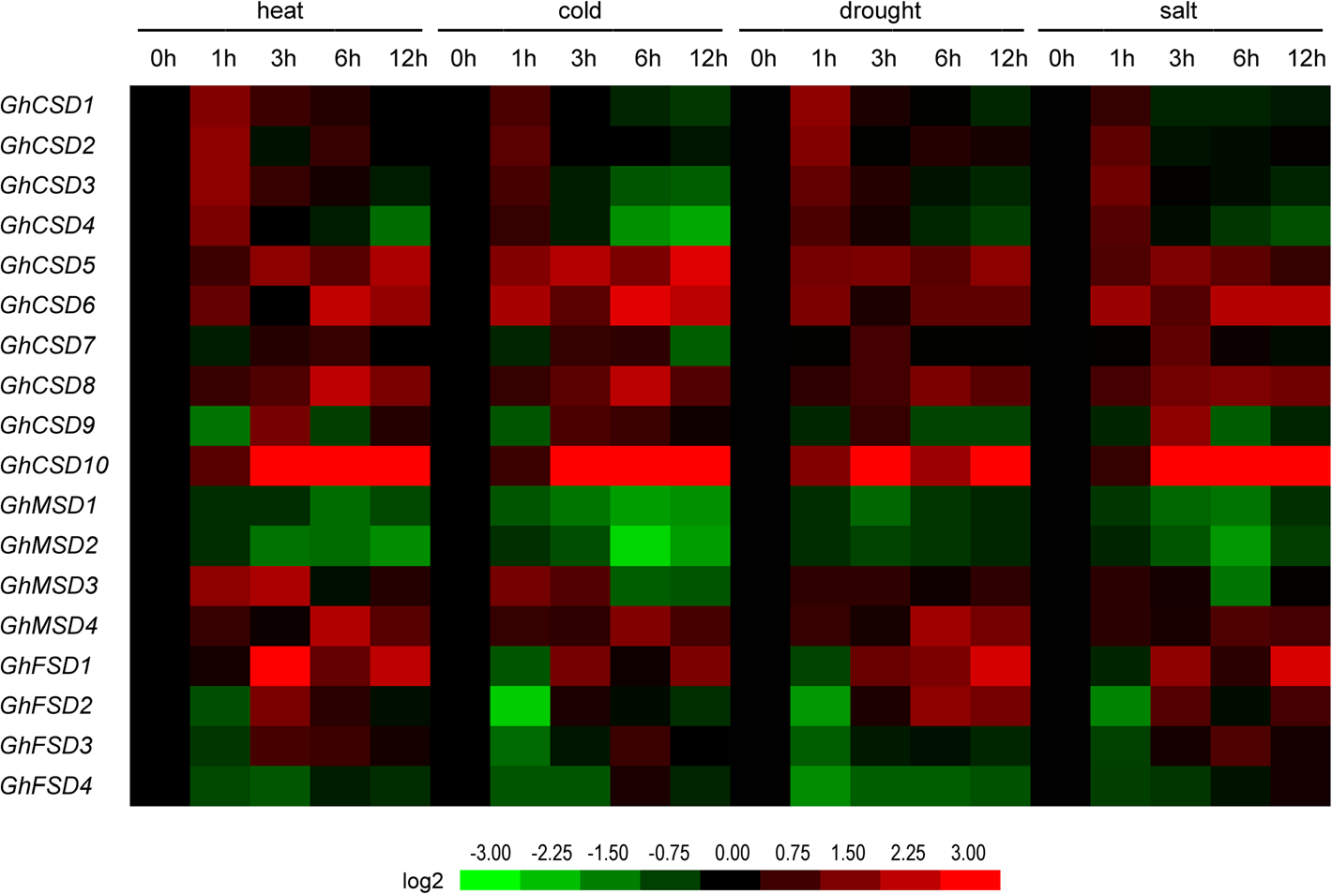
Stress-induced expression profiles of *GhSOD*s. Expressions of 18 *GhSOD*s under heat, cold, drought and salt stresses were tested. The FPKMs calculated by RNA-seq data are shown as a heat map. The colors vary from green to red, representing the scale of the relative expression levels

**Fig. 11:**
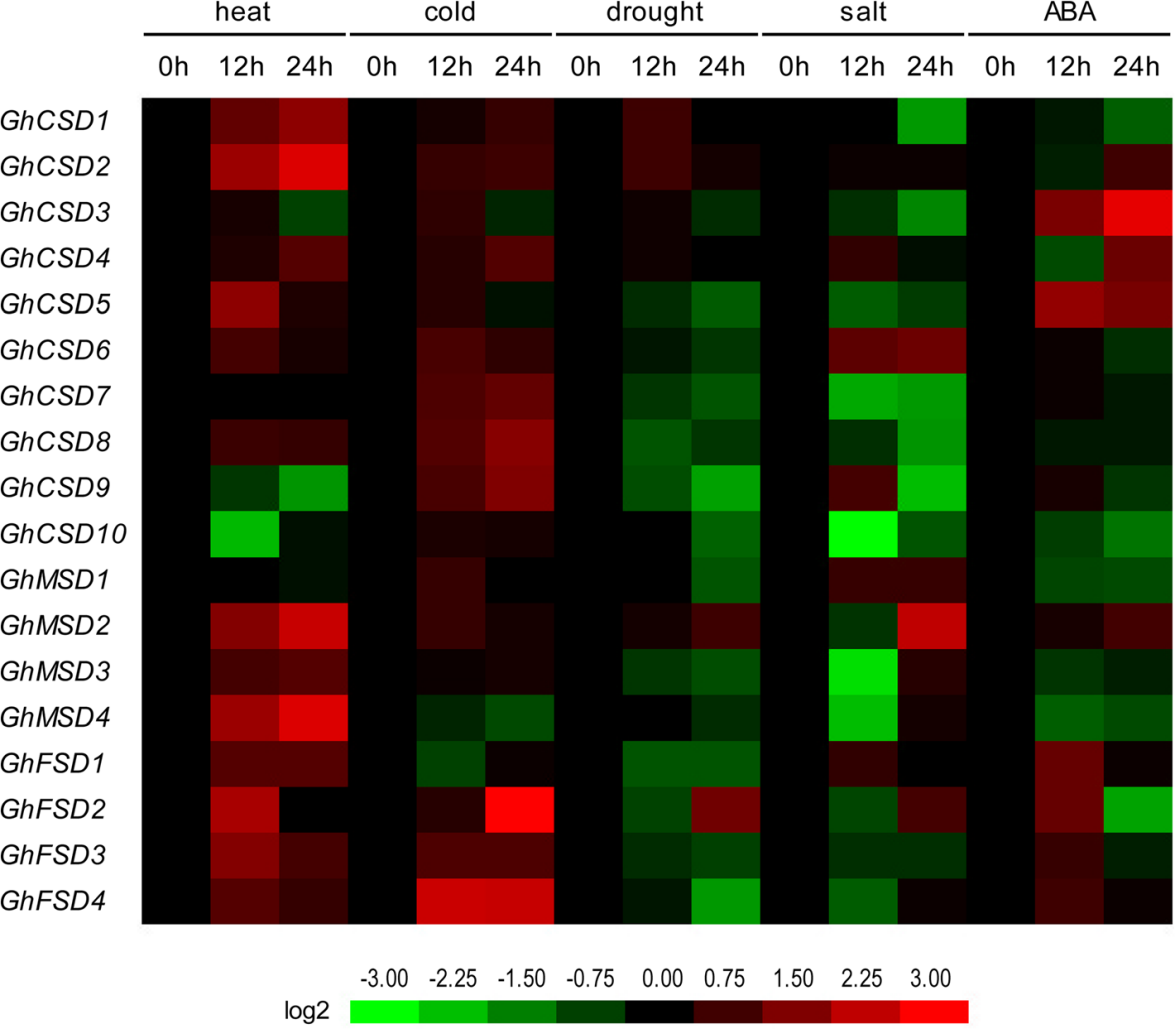
Relative transcriptional expression levels of *GhSOD*s under different abiotic treatments of *Gossypium hirsutum* ‘TM-1’ as assessed by qPCR. Each column indicates a sampling time point, and each row indicates a *GhSOD* member. The expression level of the control (at 0 h; marked in *black*) in every treatment for each gene was used as the rescaled value when calculating the relative expression levels. The colors vary from green to red representing the scale of the relative expression levels

Cotton is an important economic crop that is widely cultivated in more than 100 countries/regions. Upland cotton contributes 90% of the yield as the major cultivated cotton species, but its growth, yield and fiber quality are constantly impacted by various abiotic and biotic stresses. Previous studies reported that the plant *SOD* gene family is widely involved in biotic and abiotic stress responses [3]. High temperatures, extremely low temperatures, drought and salt stresses are the major abiotic stresses that exert detrimental effects on cotton growth and development, causing heavy losses in quantity and fiber quality. To determine the mechanisms involved in the responses of the upland cotton *SOD* gene family to heat, cold, drought and salt, we detected the expression patterns of all 18 predicted *SOD* genes in upland cotton under each of the four stresses mentioned above using RNA-seq data (Fig. 10) and qPCR (Fig. 11).

The heat map revealed that the expressions of most *SOD* genes in upland cotton were induced in the leaves under heat, cold, drought or salt stress conditions (Fig. 10). Under these stress treatments, a total of 5, 5, 4 and 5 genes, respectively, were up-regulated at early treatment time points and down-regulated after experiencing a longer stress treatment. A total of 4, 6, 2 and 2 genes, respectively, were down-regulated at early treatment time points and up-regulated after experiencing a longer stress treatment, indicating the possible existence of a feedback regulatory mechanism. A total of 5, 4 and 1 genes under heat, cold and salt stresses, respectively, were up-regulated throughout the stress treatments, while none of the genes were up-regulated throughout the drought treatment. A total of 3, 2, 4 and 3 genes under heat, cold, drought and salt stresses, respectively, were down-regulated throughout these stress treatments. Thus, the genes underlying the comprehensive expression profiles may indicate their vital functions with complex regulatory mechanisms in response to the stress treatments, acting as positive or negative regulators. In addition, there were six (*GhCSD5*/*6*/*7*/*8* and *GhMSD3*/*4*) and three (*GhCSD5*/*8 and GhMSD3*) genes that had expression levels that did not clearly change when compared with the control under drought and salt stress conditions, respectively. Thus, *GhCSD5*/*6*/*7*/*8* and *GhMSD3*/*4* may not be involved in drought and/or salt stress responses or respond to the two stresses after a long treatment time (> 12 h). Additionally, the relative expression of *GhCSD10* was up-regulated continually under heat, cold, drought and salt stresses, but it was also scarcely expressed in the leaf of the control and specific organs (Figs. 7 and 8).

The expression profiles of the 18 *GhSOD* genes, as assessed by qPCR, were detected independently under heat, cold, drought and salt stresses to validate the varying trends from 0 to 12 h and provide preliminarily information from 12 to 24 h. The expression patterns were complex (Fig. 11). All 18 *SOD* genes of upland cotton were heat-treatment responsive. The expression levels of 13 *GhSOD* genes were up-regulated at 12 h, which corroborated previous results as shown in Fig. 10. And the 13 members (*GhCSD1*/*2*/*4*/*5*/*6*/*8*, *GhMSD2*/*3*/*4* and the *GhFSDs*) were continuously up-regulated at 24 h. In contrast, five genes (*GhCSD3*/*7*/9/*10* and *GhMSD1*) had reduced expression levels at 12 h and continued to decrease at 24 h. During cold stress, 16 *GhSOD* genes were up-regulated in response to low temperature stress at 12 h. Among them, 13 genes (*GhCSD1*/*2*/*4*/*6*/*7*/*8*/*9*/*10*, *GhMSD2*/*3* and *GhFSD2*/*3*/*4*) were continuously up-regulated at 24 h, except *GhCSD3*/*5* and *GhMSD1*, which showed no differential expression and were down-regulated at 24 h. The reduced expression of *GhMSD4* at 12 h was increasingly depressed at 24 h, but *GhFSD1* showed no differential expression at 24 h. Under the drought treatment, using polyethylene glycol, most of the drought-treatment responsive genes were down-regulated in response to the stress. The transcriptional levels of eight genes (*GhCSD5*/*6*/*7*/*8*/*9*, *GhMSD3* and *GhFSD1*/*3*) were always decreased from 0 to 24 h, except *GhFSD2*, which was up-regulated at 24 h. Moreover, their expression levels of *GhCSD1*/*2* and *GhMSD2* were always increased from 0 to 24 h, and *GhCSD4* showed differential expression in response to drought stress. Under salt stress, 12 *GhSOD* genes were down-regulated at 12 h. Among them, seven genes (*GhCSD1*/*3*/*5*/*7*/*8*/*10* and *GhFSD3*) were continuously decreased at 24 h, except *GhMSD2*/*3* and *GhFSD2*, which were up-regulated at 24 h. *GhCSD6* and *GhMSD1* exhibited similar expression profiles, which increased gradually to high levels as the NaCl treatment continued. The expressions of *GhCSD4* and *GhFSD1* were dynamic, increasing quickly by 12 h, then decreasing gradually to their original levels by 24 h. However, *GhMSD4* and *GhFSD4* decreased at 12 h and increased gradually to their original levels by 24 h. In addition, *GhCSD9* was first up-regulated at 12 h, and then down-regulated from 12 h to 24 h, and *GhCSD2* showed differential expression in response to drought stress.

To analyze the potential functions of *GhSOD* genes involved in phytohormone signaling pathways, we investigated their expression levels in response to ABA (Fig. 11). Three genes (*GhCSD3*/*5* and *GhMSD2*) were up-regulated to different degrees, whereas another 6 out of the 18 *GhSOD* genes were down-regulated during the ABA treatment. Among the down-regulated genes, *GhCSD1*/*10* and *GhMSD1* showed a continuous high-level of transcript abundance over the 24 h-time-course, with peaks at 24 h. *GhFSD*s were strongly induced at 12 h, while *GhFSD1*/*4* and *GhFSD2*/3 were non-significantly induced or down-regulated at 24 h, respectively. Moreover, *GhCSD2*/*4* first showed down-regulation at 12 h, and then were up-regulated from 12 to 24 h. The expression levels of the *GhCSD6*/*7*/*9* genes exhibited slight variations at 12 h, and then were dramatically down-regulated in response to ABA treatment. The qPCR results corroborated the digital expression profiles of the transcriptome data and suggested that the *SOD* gene family of upland cotton may be important both for stress responses and developmental processes. Candidate genes from the *SOD* gene family should be selected for functional analyses of their roles in response to heat, cold, drought and salt stresses.

## Discussion

Recently, with whole-genome sequencing being completed in various plants, studies of related gene families have rapidly progressed. The *SOD*s are a critically important gene family encoding SOD proteins, which act as the first line in antioxidant systems and play important roles in responding to various environmental stimuli in plants, such as drought, salinity, cold, heat and auxin [3]. The *SOD* responses to stresses are dramatically different, depending on the different *SOD* members present, the stress and the plant species. Considering the potential functional significance of the *SOD* gene family and that several of its members have been identified in *G. raimondii* and *G. arboretum* [12], characterizing the *SOD* gene family in upland cotton is important.

### Expanded *SOD* genes family in *G. hirsutum* (AD1) genome

In this study, we identified 18 upland cotton *SOD* genes clustering into two major groups (*Cu/Zn-SODs* and *Mn/Fe-SODs*), having 9 members (5 *CSD*s, 2 *MSD*s and 2 *FSD*s) in the A-subgenome and 9 members (5 *CSD*s, 2 *MSD*s and 2 *FSD*s) in the D-subgenome. The number of *SOD* genes varies among plants, as shown in Additional file 1 [6]. Excluding the *SOD* gene family of hexaploid bread wheat, the number of *SOD* genes in diploid cottons was greater than that in Algae and Bryophyte, was similar to that in Poaceae and Cruciferae, and was less than that in banana, poplar and soybean. Whole-genome duplication (WGD) or polyploidy events occurred throughout the evolutionary history of many flowering plants [77]. For instance, one WGD event occurred at the root of the seed plants ~310 Mya, and another paleohexaploidization event at the outset of eudicots 130–190 Mya. Two WGD events occurred in monocots and may pre-date the diversification of Poaceae, and one whole genome triplication (WGT) event was probably shared by all of the core eudicots. Two recent WGDs occurred within the crucifer lineage and other lineage-specific WGD or polyploidy events occurred during the evolutionary progress of some plants, such as maize, bread wheat, banana, diploid cottons, poplar and soybean (Addition file 1) [78, 79]. These common and lineage-specific WGD or polyploidy events in plants, which generated duplicate copies of every gene, are the major factor responsible for the *SOD* gene family expansion. However, the *SOD* gene family members are not in exact proportion with the WGD or polyploidy events. Based on the phylogenetic and syntenic analyses of the upland cotton *SOD* gene family, we hypothesize that this was most likely caused by considerable gene loss, resulting from the deletions or insertions of genes, sequence divergence that occurred through point mutations, and the rearrangement or loss of chromosomes, after WGD or polyploidy events during upland cotton’s evolution. *GhCSD1*/*GhCSD2*, *GhCSD7*/*GhCSD8* and *GhCSD9*/*GhCSD10* clustered together in groups Ia, and Ic, and had first exons of identical size and high sequence similarities (Figs. 1 and 2). Hence, they may be potentially derived from segmental duplications and originated from *GrCSD2*, *GaCSD4* and *GrCSD5*, respectively.

By further analyzing the *SOD*s in the *G. raimondii* genome, we obtained more than one kind of transcript. Specifically, the number of transcripts was two for *GrCSD3*, and three each for *GrCSD1*, *GrFSD1* and *GrFSD2*. An analysis of alternative splicing (AS) suggested that *GrCSD1*, *GrFSD1* and *GrFSD2* probably underwent three basic types of alternative RNA splicing events, alternative 3’ splice site, intron retention and 5’ splice site (Additional file 12). Like AS, alternative polyadenylation (APA) and alternative transcription sites (ATSS) are other regulatory mechanisms that form transcript variants from a single gene. APA and ATSS transcripts have been detected in *SOD* genes from *Musa acuminate* [6], *Dimocarpus longan* [4], *Larix gmelinii* [80], *O. sativa* [81] and *T. aestivum* [82]. In this study, because of the absence of access to transcript variants of the upland cotton *SOD* gene family, we did not continue the analysis. However, various previous reports in plants suggested that *SOD* variants generated by AS, APA or ATSS were linked to regulating spatial- and temporal-gene expression [83], and may play a crucial role in responding to some abiotic stresses [81]. Thus, a future research project will be the cloning of the full-length cDNAs of the ‘TM-1’ *SOD* gene family, and analyzing and confirming the formation and functional mechanisms of *SOD* variants using a next-generation sequencing technique, such as RNA-seq.

Overall, we hypothesize that WGD or polyploidy events and segmental duplications contributed to the expansion of the upland cotton *SOD* gene family. Additionally, the complex regulation at the transcriptional level, such as through the AS of the pre-mRNA, generated two or more protein isoforms from single genes, contributing to the SOD protein diversity. The viable variations in SOD caused by AS, facilitated by an increasing abundance of SOD protein disorder in *Gossypium* would have provided an avenue for natural selection, which may have even facilitated the functional diversification of the upland cotton *SOD* gene family over relatively short periods on the geological time scale.

### *SOD* gene family evolution and genomes evolution in plants

During the evolution of *Gossypium*, many WGD or polyploidization events that caused species differentiation through polyploid formation occurred, including an ancestral seed plant WGD, an ancestral angiosperm WGD and one WGT that was probably shared by all of the core eudicots. Shortly after divergence from cacao, the Gossypium lineage experienced a five- to six-fold ploidy increase and a subsequent diversification into eight diploid genome groups, including A–G and K. A-genome diploids native to Africa and Mexican D-genome diploids diverged approximately 5–10 Mya. These two species were reunited geographically approximately 1–2 Mya by the transoceanic dispersal of an A-genome ancestor resembling *G. arboreum* to the New World. Then, hybridization with a native D-genome species resembling *G. raimondii* and chromosome doubling occurred, forming the common ancestor of *G. hirsutum* (Upland) and *G. barbadense* (Egyptian, Sea Island and Pima) cottons [18, 84]. Combined with the analyses of phylogenetic and syntenic relationships of *SOD* gene families from *G. hirsutum*, *G. arboreum* and *G. raimondii* (Figs. 1 and 2), we showed that the overall gene order and the co-linearity of the *SOD* gene family in upland cotton were largely conserved between the A- and D-subgenomes and the extant D-progenitor genome (*G. raimondii*). Thus, we believe that, during the evolution of the *Gossypium SOD* gene family, one gene was native to the D-genome ancestor and the other one was native to the A-genome ancestor for the alleles of the *GhMSD*s, *GhFSD*s, and *GhCSD3*/*GhCSD4* and *GhCSD5*/*GhCSD6*. Meanwhile, *GhCSD1*/*GhCSD2*, *GhCSD7*/*GhCSD8* and *GhCSD9*/*GhCSD10* separately originated from *GaCSD2*, *GaCSD4* and *GrCSD5* and were formed by gene duplication. However, *GrCSD2*, *GrCSD4* and *GaCSD5* were lost after the reuniting of the A- and D-genome ancestors, which may be caused by the biased fractionation of the genome [85] and/or subgenome dominance [86]. This is just one of our hypotheses, because *G. arboreum* and *G. raimondii* are the only two extant progenitor relatives, and the exact donor species that led to the formation of the tetraploid cotton species 1–2 Mya no longer exists [84].

The *SOD* genes are an important gene family encoding SOD proteins, which play a major role in the defense system against oxidative stress in plants and are ubiquitous in every cell of all plant types. Several *SOD* homologs have been discovered in many plant species, including green algae, moss, Amborella, Arabidopsis, rice, Brachypodium, durra, soybean and poplar. Based on the phylogenetic relationships of the *SOD* gene family from upland cotton and the nine flagship species considered pivotal references for understanding plant evolution, the *SOD* gene family of plants was divided into two major groups (*Cu*/*Zn*- and *Mn*/*Fe*-). *Cu*/*Zn*-*SOD*s contained three subgroups (Ia, Ib and Ic) and *Mn*/*Fe*-*SODs* contained two subgroups (IIa and IIb) (Additional file 9). This grouping agreed with the results shown in Fig. 1. Additionally, the phylogenetic relationships of *SOD* genes in each subgroup were consistent with the evolution of plants (Additional files 9). Remarkably, the green algae *C. reinhardtii* had 5 *Mn*-*SOD*s, 1 *Fe*-*SOD* and no *Cu*/*Zn*-*SOD*s, and the 5 *Mn*-*SOD*s possessed high sequence similarity. This suggested that the *Mn*- and *Fe*-*SOD*s were older than the *Cu*/*Zn*-*SOD*s because they are thought to have been generated from the same ancestral enzyme, while the *Cu*/*Zn*-*SOD*s evolved separately and did not possess such a similarity [57]. Meanwhile, the number of *Mn*-*SOD*s in green algae was greater than in all of the 18 plants, and the number of *Cu*/*Zn*-*SOD*s was greater than that of the *Mn*- and *Fe*-*SOD*s in plant evolution (Additional file 1). On the early earth, the primitive levels of oxygen could not exceed that of molecular oxygen (~0.001%) present at the atmospheric level, and metal ions, such as Mn and Fe, were relatively easy to obtain [87]. Accordingly, Mn/Fe-SODs were in a dominant position, especially Mn-SODs. With the evolution of earth, the rise of oxygen from the primitive levels could only be associated with oxygenic photosynthesis that was well-established on the earth at least 3.5 thousand Mya, thereby contributing to the concentration of ROS. Furthermore, Mn and Fe ions participated in the electron transport chain as an indispensable part of the two photosystems’ reaction centers. Meanwhile, plants were facing a more complicated and uncertain [88]. Therefore, Cu/Zn-SODs separated from the family and occupied the dominant position during plant evolution to respond to the various abiotic and biotic stresses. Thus, we hypothesize that with the ever-changing earth environment and the plant evolution, the *SOD* gene family expanded and experienced biased fractionation after multiple rounds of polyploidization events in plant genomes, particularly massive *Mn*-*SOD* gene losses. Moreover, *Cu*/*Zn*-*SOD*s evolved separately in bryophyta with the functional differentiation, caused by including neofunctionalization and/or epigenetic modifications of transposable elements, of *Mn*-*SOD*s to response to various stresses [89].

Thus, comparative sequence and phylogenetic analyses of the three plant *SOD* isoforms suggested that *Mn*- and *Fe*-*SOD*s may have arisen from common ancestral enzymes, whereas *Cu*/*Zn*-*SOD*s evolved separately in bryophyta. The two major groups must have evolved independently.

### Gene expression patterns of *GhSODs*

The spatiotemporal expression patterns of *SOD*s have been determined in many species, such as *Arabidopsis*, longan and banana. For banana, 11 of 12 *MaSOD*s were expressed at relatively high levels in the leaf, pseudostem and root [6]. All *DlSOD* types with moderate and stable expression, such as *DlCSDs*, *DlMSD* and *DlFSD*, mediated development during the four primary developmental stages of longan early somatic embryogenesis [4]. In this study, we analyzed the transcript levels of all of the *GhSOD*s in 12 different organs/tissues and some different development stages. Most of the *GhSOD*s are expressed predominantly in cotyledon, leaf, flower and seed, with relatively weak expressions in the stem, root and fiber. Cotyledon and leaf are major vegetative organs that play fundamental roles in the maintenance of plants life through photosynthesis. Additionally, flower and seed undergo organogenesis, germination and other metabolic processes as common reproductive organs. Large amounts of ROS are often generated during these vital processes and, therefore, the expression levels of the *GhSOD*s were relatively high in these organs/tissues. Our data indicated that *GhCSD1* and *GhCSD2* were involved in whole-plant development, and that most of the other *GhSOD*s may play important roles in seed, root, ovule and fiber development (Fig. 8). Additionally, the tissue/organ-specific and stage-specific expression profiles of the GhSODs suggested that the expression yields were in accord with their phylogenetic clustering results (Figs. 1 and 2a), namely, that the genes that were grouped in one of the Ia, Ib, Ic, IIa or IIb classes shared similar expression patterns in different tissues/organs and developmental stages. Thus, the *SOD* genes of upland cotton were expressed with a certain degree of temporal- and spatial-specificity and played important roles in different tissues/organs and developmental stages.

Previous studies reported that plants could maintain the ROS balance by ROS-scavenging systems caused by abiotic stress that mainly involved three types of SODs, Cu/Zn-SOD, Mn-SOD and Fe-SOD [90]. The expression analysis suggested that every *GhSOD* gene responded to at least one abiotic stress performed in this study (heat, cold, drought or salt) (Figs. 10 and 11), which coincided with the GO annotations of the SODs (Fig. 4 and Additional file 11) and bioinformatics analysis of putative *GhSOD* promoters (Fig. 5 and Additional file 2). The results of the GO annotations revealed that the *SOD* gene family of upland cotton may be involved in biological processes, including responses to biotic and abiotic stimuli, such as bacterium, light, UV-B, salt, ozone and sucrose metabolic processes. Moreover, according to the analysis of putative *GhSOD* promoters, the promoters of the *GhSOD* gene family harbored more kinds and numbers of *cis*-elements involved in abiotic stresses, including the *cis*-elements involved in low-temperature responsiveness, *cis*-elements involved in heat stress responsiveness, MYB-binding sites involved in drought-inducibility and *cis*-elements involved in the ABA responsiveness, which could explain why *GhSOD* exhibited obvious responses to the four abiotic stresses. Notably, compared with *GhMSD*s and *GhFSD*s, *GhCSD*s showed obvious expression changes under all four abiotic stresses. This indicated that *GhCSD*s may play a predominant antioxidant role in upland cotton. Similar observations were also detected in other plant *Cu/Zn-SOD* genes [6, 91].

Hormone-responsive TFs regulated the expression of target genes by combining with their corresponding *cis*-elements in the promoters during various stresses. The analysis of putative GhSOD promoters predicted that 10 *GhSOD* promoters (*GhCSD8*/*9*/*10*, *GhMSD1*/*2*/*4* and *GhFSD*s) harbored one to three ABREs, a *cis*-acting element involved in the ABA responsiveness, indicating that these genes probably participate in ABA responses. Moreover, the expression of eight other *GhSOD*s, which had no ABREs, showed differential expression inductions during the ABA treatment, suggesting that there were other regulatory mechanisms responding to ABA, such as miRNA. Tang et al. reported that miR398 negatively regulated the expression of its target genes (*Cu/Zn-SOD*s) in response to ABA [63]. In this study, we detected that ghr-miR398 targeted *GhCSD7*, *GhCSD8*, *GhCSD9* and *GhCSD10*, and that all of the targeted sites were located in CDS regions, which may explain why some of the *GhSOD*s responded to the ABA treatment. We, therefore, proposed that the *GhSOD*s expression in response to ABA may work in coordination with ABREs and miRNAs. In addition, there were 10 other hormone-responsive cis-elements (TGA-element, ERE, CGTA-motif, CGTCA-motif, TGACG-motif, GARE-motif, P-box, TATC-box, AuxRR-core and TCA-element) located in the putative promoter regions of the *GhSOD*s. Their transcriptional regulation under IAA, GA3, methyl jasmonate and SA treatments needs to be investigated in further experiments. We hypothesize that the *SOD* genes of upland cotton probably participate in phytohormone signaling pathways.

### Regulation of *GhSOD* genes expression

Plants utilized a complex regulatory network of transcriptional, posttranscriptional, translational and posttranslational gene expression programs to respond to various abiotic and biotic stresses. Characterization of TFs and stress-responsive regulatory *cis*-elements offers insights into the upstream regulation of *GhSOD*s. *Cis*-acting elements involved in stress-induced gene expression were predicted in the promoter regions of the *GhSOD* genes. Additionally, MYB-type TF-binding sites were obtained in all 18 *GhSOD*s (Fig. 5). The various functions for the MYBs has been investigated in numerous plant species using both genetic and molecular analyses. MYB plays crucial roles in different processes, including primary and secondary metabolisms, such as the regulation of various phytochemical biosynthesis pathways, regulation of several developmental processes, such as cell fate determination in root hairs, secondary cell wall biosynthesis, establishment of the axillary branch patterning, leaf proximodistal axis and anther development, and responses to environmental stimuli [92, 93]. For instance, in upland cotton, *GhMYB*s may be involved in regulating specialized outgrowths of epidermal cells, including cotton fiber development during different developmental stages [93], and several *MYB* genes in cotton were differentially expressed under salt and drought stress treatments [92]. Additionally, many *MYB* genes in other plant species were involved in regulating responses to biotic and abiotic stresses, and enhanced the tolerance to stresses in transgenic plants [94, 95]. *GhSOD*s may also be involved in cell wall growth and development processes, including cotton fiber development [96], and in responding to environment stresses and enhancing the tolerance of transgenic plants expressing *SOD* genes against oxidative stress [3]. Using bioinformatics analyses of putative *GhSOD* promoters, we obtained several binding sites of MYB TFs. The expressions of *SOD* genes may be regulated by MYB-type TFs. Hence, we hypothesized that MYB could control the expression of upland cotton *SOD* genes in response to environmental stresses and regulate the process of cotton fiber development at the transcriptional level. The functions of most MYBs in higher plants remains unclear, and the roles of MYB in *SOD* regulatory networks, as well as their inferred functions, remains to be fully elucidated through the use of inducible systems in high-throughput expression and interaction studies, combined with bioinformatics and systems analyses in *G. hirsutum*.

miRNAs are a diverse category of nuclear-encoded small RNAs that play multiple, central functions in plant development, stress responses, and many other biological processes. Mature miRNAs facilitate the cleavage of bound target genes and/or trigger translational repression by binding to the 5’ UTR, 3’ UTR or coding regions of the target mRNAs [97]. Currently, miRNA-mediated posttranscriptional gene regulation is particularly interesting because miRNAs can regulate several protein-coding genes implicated in the same pathway.

In our study, we obtained some predicting miRNA target sites locating at introns. For instance, novel_miR_47/50/51, identified in *G. hirsutum* inoculated with verticillium wilt [72], targeted *GhFSD1* and *GhFSD2* with sites in intron (Additional file 2), and, *GhFSD3* and *GhFSD4* were targeted by novel_mir_4246 and m0362 with sites in intron (Fig. 6, Additional file 2). Because we analyzed the alternative splicing of *SOD* genes in *G. raimondii* and found that there was a basic type of alternative RNA splicing event, intron retention (Additional file 12). So, we conjectured the alternative RNA splicing event existing in *SOD* genes of upland cotton. Then, the introns had the possibility of being exons erroneously remains in the mature mRNA and encoded amino acids in frame with the neighboring exons. Therefore, we exhibited the predicting miRNA target sites of *GhSODs*, located at introns (Fig. 6). It was, of course, investigated in further experiments.

In addition, other miRNAs that were predicted to target to *GhSOD*s were also involved in stress responses and biological processes. More concretely, ghr-miR414c, ghr-miR7267, m0081, m0166 and m0362 were involved in cotton fiber differentiation and development [74, 98], and ghr-miR3 regulated the expression of targeted genes during cotton somatic embryogenesis [99]. As a specific well-studied example, miR398 is involved in responses to diverse abiotic and biotic stresses, such as oxidative, salt, heat and ABA, as well as and water, Cu and phosphate deficiencies, and the additions of sucrose, paraquat, ozone or plant pathogen [63, 73, 100–103]. The mechanisms of miR398 and their targeted genes involved in biotic and abiotic stresses have been widely researched in *A. thaliana*. miR398 targets two *Cu/Zn-SOD*s (cytosolic *CSD1* and chloroplast-localized *CSD2*) and the Cu chaperone for SOD, *CCS1*, which is activated CSD by delivering a Cu cofactor to the three Cu/Zn-SODs. Additionally, miR398 was transcriptionally down-regulated in *Arabidopsis* under other oxidative stress conditions, and the decreased miR398 level resulted in the accumulation of *CSD1* and *CSD2* mRNAs, which were important for oxidative stress tolerance [104]. In contrast to the down-regulation of miR398 during oxidative stress, an increase in miR398 levels, regulated by the upstream TFs, was observed during Cu deficiency, leading to the down-regulation of *CSD1*, *CSD2* and *CCS1* mRNA expression levels. CCS1 was necessary for the activation of both CSD1 and CSD2 in *Arabidopsis*; thus, the miR398-guided cleavage of *CCS1* mRNA decreased the delivery of Cu to CSD and further down-regulated CSD activities [71].

To provide a comprehensive model for the role of ghr-miR398 in oxidative stress and nutrient homeostasis, we identified the *CCS* genes and predicted the targeted sites of miRNAs in the genome of upland cotton (Additional files 2 and 13). Two *CCS* genes were identified, named *GhCCS1* and *GhCCS2*, which were located in chromosomes A8 and D8, respectively. Both ghr-miR398 and novel_mir_1205 targeting *GhCCS1* and *GhCCS2*, respectively, were obtained and the complementary sites were located in the last exon and the 3’ UTR. Our results were consistent with corresponding reports in *Arabidopsis* [71], suggesting that the regulation of the *CCS* gene by miR398 was conserved in *Arabidopsis* and upland cotton. However, because of differences in species, growth conditions and treatment methods, the relationships between differential biotic and abiotic stresses, as well as miRNA-mediated stress tolerance in cotton, needs further investigation.

## Conclusions

We identified 18 *SOD* genes, including three types of plant *SODs* (*Cu/Zn-SODs*, *Mn-SODs* and *Fe-SODs*), that were divided into five subgroups and distributed on 12 of the 26 chromosomes in the upland cotton genome. WGD and polyploidy events, as well as segmental and tandem duplications, contributed to the expansion of the *SOD* family. Additionally, the complex regulation at the transcriptional level, such as the AS of the pre-mRNA, was a contributory cause of SOD protein diversity. We analyzed the *SOD* gene family’s evolution during the *Gossypium* and genomic evolution in plants. Based on the results of GO annotations, the *SOD*s of upland cotton were involved in abiotic/biotic stimuli and reproductive development processes, and were regulated by miRNAs. A promoter sequence analysis identified many abiotic/biotic stresses and hormonal-responsive *cis*-acting elements in the promoter regions of the *GhSOD*s, but different members harbored distinct types and numbers, which indicated that the 18 *GhSOD*s were differentially regulated. Additionally, the expression profiles of the upland cotton *SOD* gene family were detected using RNA-seq and qPCR data, and suggested that distinct members of the family exhibited different expression patterns in response to abiotic and hormonal stresses, which revealed that *GhSOD*s play roles in different aspects of upland cotton abiotic stress tolerance and hormonal signaling. Moreover, we predicted and analyzed the miRNA-mediated posttranscriptional regulation of the gene family in this species.

Thus, our study helps lay the foundation for further cloning and functional verification of the *GhSOD* gene family by overexpression and knock down/out using RNAi or genome editing tools, such as CRISPR-Cas9, and provides new insights into the evolution and divergence of the *SOD* genes in plants. Moreover, these results may increase the understanding of the molecular basis of many important agronomic upland cotton traits, such as fiber development, verticillium and fusarium wilt resistance, and other physiological processes.

## Additional files

Additional file 1: Table S1. Gene numbers of *SOD* gene family in 18 plant genomes. (PDF 3335 kb)

Additional file 2: Table S6. The predictions of *GhSODs* and *GhCCSs* targeted by the miRNAs of *G. hirsutum*. (PDF 3305 kb)

Additional file 3: Table S7. The details of whole transcriptome sequencing data of *G. hirsutum*acc. TM. (PDF 3311 kb)

Additional file 4: Table S8. Gene-specific primers used for quantitative real-time PCR. (PDF 3302 kb)

Additional file 5: Figure S1. The alignment of the genome sequence and mRNA sequence of *GhCSD7* with *GhCSD8* and its downstream 5000 bp. (PDF 3302 kb)

Additional file 6: Table S2. Motif sequences of GhSOD proteins identified by MEME tools. (PDF 3302 kb)

Additional file 7: Figure S2. Conserved motifs analysis of GhSOD proteins. (PDF 3330 kb)

Additional file 8: Table S3. The details of sequences used in the phylogenetic relationship analysis. (PDF 3305 kb)

Additional file 9: Figure S3. Maximum-likelihood (ML) phylogenetic tree constructed for 70 *SOD* sequences from 9 flagship species and upland cotton. (PDF 3302 kb)

Additional file 10: Table S4. Orthologous *SOD* gene pairs of *G. hirsutum*, *G. arboreum*, and *G. raimondii*. (PDF 3451 kb)

Additional file 11: Table S5. Gene Ontology annotations of 18 upland cotton *SOD* genes. (PDF 3304 kb)

Additional file 12: Figure S4. Analysis of alternative splice in 4 *GrSOD* genes with at least two transcript variants. (PDF 3301 kb)

Additional file 13: The relevant information of upland cotton *CCS* genes. (PDF 3302 kb)

## Abbreviations

aa: Amino acid
ABA: Abscisic acid
*Amt*: *Amborella trichopoda*
*At*: *Arabidopsis thaliana*
*Bd*: *Brachypodium distachyon*
bp: base pair
CCS: A cooper chaperone for cooper-zinc superoxide dismutase
CDS: Coding sequence
*Cr*: *Chlamydomonas reinhardtii*
CRISPR: Clustered regularly interspaced short palindromic repeats
CSD: Cooper-zinc superoxide dismutase
CZ: Cu/Zn-superoxide dismutase domain
DOA: Day of anthesis
DPA: Days post-anthesis
FPKM: Fragments per kilobase of transcript per million mapped reads
FSD: Iron superoxide dismutase
*Ga*: *Gossypium arboretum*
*Gh*: *Gossypium hirsutum*
*Gm*: *Glycine max*
*Gr*: *Gossypium raimondii*
*Hv*: *Hordeum vulgare*
IMA: Fe/Mn-SODs, alpha-hairpin domain
IMC: Fe/Mn-SODs, C-terminal domain
kb: Kilobase
*Ma*: *Musa acuminate*
miRNA: MicroRNA
MSD: Manganese superoxide dismutase
MW: Theoretical molecular weight of proteins
Mya: Million years ago
NCBI: National Center for Biotechnology Information
nt: Nucleotide
ORF: Open reading frame
*Os*: *Oryza sativa*
PCR: Polymerase chain reaction
PCW: Primary cell wall
p*I*: Isoelectric point
*Pp*: *Physcomitrella patens*
*Pt*: *Populus trichocarpa*
qRT-PCR: Quantitative reverse-transcription polymerase chain reaction
RNAi: RNA interference
ROS: Reactive oxygen species
*Sb*: *Sorghum bicolor*
SCW: Secondary cell wall
*Si*: *Setaria italic*
SOD: Superoxide dismutases
*Ta*: *Triticum aestivum*
TF: Transcription factors
TFBS: Transcription factor binding sites
UBQ: Ubiquitin extension protein
UTR: Untranslated regions
WGD: Whole genome duplication
WGT: Whole genome triplication
ZF: C2H2-type zinc finger domain
*Zm*: *Zea mays*
