# Supplementary material for: Genome-wide characterization and expression analyses of superoxide dismutase (SOD) genes in Gossypium hirsutum
Source: BMC Genomics. 2017 May 12;18:376. doi: 10.1186/s12864-017-3768-5 (PMC5429560; doi:10.1186/s12864-017-3768-5)
Supplement: Supplementary file 1 — Gene numbers of SOD gene family in 18 plant genomes. (PDF 3335 kb) [file 12864_2017_3768_MOESM1_ESM.pdf]

Additional file 1: Table S1. Gene numbers of *SOD* gene family in 18 plant genomes.

	Plant speicies																
Gene group	Algae	Moss	Amborellaceae	Monocots								Edudicots					
	Cr	Pp	Amt[1,2]	Bd[1-3]	Hv[1-3]	Os[1-3]	Sb[1-3]	Si[1-3]	Zm[1-4]	Ta[1-5]	Ma[1-4,6]	At[1,2,7,8]	Ga[1,2,7,9]	Gr[1,2,7,9]	**Gh**[1,2,7,9,10]	Pt[1,2,7,11]	Gm[1,2,12,13]
Cu/Zn-SOD	0	3	3	3	3	4	4	4	5	10	6	3	5	5	**10**	6	6
Mn-SOD	5	1	1	1	1	1	1	1	2	2	4	2	2	2	**4**	2	2
Fe-SOD	1	2	2	2	2	2	2	2	3	6	2	3	2	2	**4**	3	5
**Total**	**6**	**6**	**6**	**6**	**6**	**7**	**7**	**7**	**10**	**18**	**12**	**8**	**9**	**9**	**18**	**11**	**13**

Cr, *Chlamydomonas reinhardtii*; **Pp**, *Physcomitrella patens*; **Amt**, *Amborella trichopoda*; **Bd**, *Brachypodium distachyon*; **Hv**, *Hordeum vulgare*; **Os**, *Oryza sativa*; **Sb**, *Sorghum bicolor*; **Si**, *Setaria italica*; **Zm**, *Zea mays*; **Ma**, *Musa acuminata*; **Ta**, *Triticum aestivum*; **At**, *Arabidopsis thaliana*; **Ga**, *Gossypium arboretum*; **Gr**, *Gossypium raimondii*; **Pt**, *Populus trichocarpa*; **Gm**, *Glycine max*; **WGD**, whole-genome duplication; **Mya**, million years ago.

[1], ancestral seed plant WGD (ζ) ~310 Mya; [2], the paleohexaploidization event (ε) common to all eudicots 130-190 Mya; [3], two WGD in monocots (σ and ρ) inferred to have pre-dated the diversification of Poaceae; [4], allotetraploid in maize ~13 Mya; [5], one lineage-specific polyploid event (hexaploid) in bread wheat; [6], three lineage-specific WGD rounds occurred during banana evolution; [7], one whole genome triplication (WGT) event (γ) probably shared by all core eudicots; [8], two recent WGDs (α and β) within the crucifer lineage; [9], one penta- or hexaploid duplication event in the *Gossypium* lineage after splitting from cacao lineage ~60 Mya; [10], neoallopolyploids reuniting divergent *Gossypium* genomes 1-2 Mya; [11], one lineage-specific WGD event occurred during poplar evolution; [12], the legume-specific WGD event 58 Mya; [13], the *Glycine* WGD event 13 Mya.
