# Supplementary material for: Genome-wide characterization and expression analyses of superoxide dismutase (SOD) genes in Gossypium hirsutum
Source: BMC Genomics. 2017 May 12;18:376. doi: 10.1186/s12864-017-3768-5 (PMC5429560; doi:10.1186/s12864-017-3768-5)
Supplement: Supplementary file 2 — The predictions of GhSODs and GhCCSs targeted by the miRNAs of G. hirsutum. (PDF 3305 kb) [file 12864_2017_3768_MOESM2_ESM.pdf]

Additional file 2: Table S6. The predictions of *GhSODs* and *GhCCSs* targeted by the miRNAs of *G. hirsutum* .

Target_Acc.	miRNA_Acc.	Expectation	UPE	miRNA		Target			miRNA_aligned_fragment	Target_aligned_fragment	Inhibition
				start	end	start	end	region			
GhCSD3	ghr-miRnF	3	17.469	1	20	253	272	CDS	UCGGACUGGAUUUGUUGACA	UGUCAACAGGUAAAGUCCGA	Translation
	novel_mir_1200	3	17.829	1	20	273	292	CDS	AGGAGAAAAAAAUCUGAUUU	AAGUUGGAUUUGUUUUUCCU	Translation
GhCSD4	novel_mir_1200	3	17.525	1	20	273	292	CDS	AGGAGAAAAAAAUCUGAUUU	AAGUUGGAUUUGUUUUUCCU	Translation
GhCSD7	m0166	2.5	10.736	1	23	5577	5599	CDS	CAGAGUUGGUGUGUAGAGGAUAG	UUGUCUUCUCCACACCAGCUUUG	Cleavage
	ghr-miR398	3	19.56	1	19	1058	1077	CDS	UGUUUAUCAGG-CACCCCU	CGGGUGACCUGGGAAACA	Cleavage
GhCSD8	novel_mir_2733	3	15.01	1	20	1257	1276	CDS	AAGAGAAAUGAUUGUAUGAA	UCCGUGCAUUCAUUUCUCUU	Cleavage
	ghr-miR398	3	19.56	1	19	1064	1083	CDS	UGUUUAUCAGG-CACCCCU	CGGGUGACCUGGGAAACA	Cleavage
GhCSD9	ghr-miR398	3	19.56	1	19	1223	1242	CDS	UGUUUAUCAGG-CACCCCU	CGGGUGACCUGGGAAACA	Cleavage
GhCSD10	ghr-miR398	3	19.85	1	20	1076	1095	CDS	GUGUUUAUCAGG-CACCCCU	CGGCTGACCTGGGAAACATA	Cleavage
GhFSD1	novel_mir_1290	0.5	19.488	1	23	2042	2064	CDS	UUUGAUCUAACGUACAGAGACUA	UAGUCUUUGUACGUUAGAUCAAA	Cleavage
	ghr-miR1324	3	9.176	1	21	2085	2105	CDS	UAGAAAUGGAUGGAAUUUUUA	UUAAAAUUUCAUCCAUUUAUA	Cleavage
	novel_miR_47	2	11.588	1	21	2089	2109	CDS	ACAGUAGAAAUGGAUGGAAUU	AAUUUCAUCCAUUUAUACUGU	Cleavage
	novel_mir_4051	1.5	17.119	1	21	2169	2189	CDS	UUGUUAAAAGUUUCAUCCAUU	AAUUGAUGAAAUUUUUAACAA	Cleavage
	novel_miR_51	2.5	12.88	1	21	2195	2215	CDS	UUAGAUCAAAGAGCAAACCGG	CCAGUUUACUCUUUGAUUUAA	Cleavage
	ghr-miR7267	2	14.857	1	20	2203	2222	CDS	UUGUACGUUAGAUUAAAGAG	CUCUUUGAUUUAACGUGCAG	Cleavage
	novel_mir_1378	2	18.99	1	22	2224	2245	CDS	UCAAAAAAUGGGCAAAGUAGUC	GACUAAUUUGCUUAUUUUUUGA	Cleavage
	novel_miR_50	2	21.463	1	21	2262	2282	CDS	UUGUACUAGGAGUCGGAUUGC	GCAAUCCAAUUCUUAGUACAA	Cleavage
	ghr-miR414c	2	6.78	1	21	2831	2851	CDS	UCAUCAUCAUCAUCACCUUCA	UGAAGAAGAUGAUGAUGAUGA	Cleavage
GhFSD2	m0081	3	13.201	1	24	186	209	CDS	CAAGAUUUGGGGUUUUGGGUUCGG	CCAUGUCUAAAACCCCAGGUUUUG	Cleavage
	novel_mir_1290	0.5	20.797	1	23	2021	2043	CDS	UUUGAUCUAACGUACAGAGACUA	UAGUCUUUGUACGUUAGAUCAAA	Cleavage
	novel_mir_5016	0.5	16.137	1	21	2035	2055	CDS	ACCGAUUUGCUCUUUGAUCUA	UAGAUCAAAGAGCAAAUUGGU	Cleavage
	novel_miR_47	3	11.628	1	21	2067	2087	CDS	ACAGUAGAAAUGGAUGGAAUU	AAUUUCAUCCAUUUAUACUGA	Cleavage
	novel_miR_51	1.5	13.577	1	21	2173	2193	CDS	UUAGAUCAAAGAGCAAACCGG	CCAGUUUGCUCUUUGAUUUAA	Cleavage
	novel_mir_1378	1	17.537	1	22	2202	2223	CDS	UCAAAAAAUGGGCAAAGUAGUC	GACUAAUUUGCCCAUUUUUUGA	Cleavage
	novel_miR_50	2	21.389	1	21	2240	2260	CDS	UUGUACUAGGAGUCGGAUUGC	GCAAUCCAAUUCUUAGUACAA	Cleavage
GhFSD3	novel_mir_4246	1	18.955	1	21	1029	1049	CDS	UUUUCAACUCUGCCAAGCAAU	AUUGCUUGGUAGGGUUGAAAA	Cleavage
	m0362	3	6.177	1	24	1191	1214	CDS	AAGUGUGCUUGGUAGGAAAGAUAA	UUUUCCUUCAUACCAAGCACACUC	Cleavage
GhFSD4	novel_mir_4246	1	19.404	1	21	1029	1049	CDS	UUUUCAACUCUGCCAAGCAAU	AUUGCUUGGUAGGGUUGAAAA	Cleavage
	m0362	2	6.266	1	24	1191	1214	CDS	AAGUGUGCUUGGUAGGAAAGAUAA	UUUUCUUUCAUACCAAGCACACUC	Cleavage
GhMSD1	ghr-miR3	2.5	16.915	1	20	2045	2064	CDS	UCUUGUACUGCAUCAUAACU	UAUUAUGGUGCAGUACAAGA	Cleavage
GhMSD2	ghr-miR3	2.5	15.124	1	20	2055	2074	CDS	UCUUGUACUGCAUCAUAACU	UAUUAUGGUGCAGUACAAGA	Cleavage
GhMSD3	ghr-miR3	2.5	15.687	1	20	2655	2674	CDS	UCUUGUACUGCAUCAUAACU	UUUUAUGGUGCAGUACAAGA	Cleavage
GhMSD4	ghr-miR3	2.5	18.554	1	20	2641	2660	CDS	UCUUGUACUGCAUCAUAACU	UUUUAUGGUGCAGUACAAGA	Cleavage
GhCCS1	ghr-miR398	3	14.26	1	20	2632	2650	CDS	UGUGUUCUCAGGUCACCCCU	AUUGGCGACCUGGGAACUCA	Cleavage
	novel_mir_1205	2	3.515	1	23	5001	5021	3' UTR	AGAGGGAGAAGCAGAAGAGAAUA	UUUUCUCUUCUUCUUCUUCUUCU	Cleavage
GhCCS2	ghr-miR398	3	14.34	1	20	2504	2523	CDS	UGUGUUCUCAGGUCACCC-CU	AGUUGGCGACCUGGGAACUCA	Cleavage
	novel_mir_1205	2	2.894	1	23	4734	4756	3' UTR	AGAGGGAGAAGCAGAAGAGAAUA	UUUUCUCUUCUUCUUCUUCUUCU	Cleavage
