# Supplementary material for: Genome-wide characterization and expression analyses of superoxide dismutase (SOD) genes in Gossypium hirsutum
Source: BMC Genomics. 2017 May 12;18:376. doi: 10.1186/s12864-017-3768-5 (PMC5429560; doi:10.1186/s12864-017-3768-5)
Supplement: Supplementary file 3 — The details of whole transcriptome sequencing data of G. hirsutumacc. TM. (PDF 3311 kb) [file 12864_2017_3768_MOESM3_ESM.pdf]

Additional file 3: Table S7. The details of whole transcriptome sequencing data of *G. hirsutum* acc. TM-1.

Classification	Accession	Description
stress-induced expression pattern	SRX849553	Gossypium hirsutum cv TM1 cotton RNA-seq, 1 h treated with PEG
	SRX849554	Gossypium hirsutum cv TM1 cotton RNA-seq, 3 h treated with PEG
	SRX849555	Gossypium hirsutum cv TM1 cotton RNA-seq, 6 h treated with PEG
	SRX849556	Gossypium hirsutum cv TM1 cotton RNA-seq, 12 h treated with PEG
	SRX849549	Gossypium hirsutum cv TM1 cotton RNA-seq, 1 h treated with salt
	SRX849550	Gossypium hirsutum cv TM1 cotton RNA-seq, 3 h treated with salt
	SRX849551	Gossypium hirsutum cv TM1 cotton RNA-seq, 6 h treated with salt
	SRX849552	Gossypium hirsutum cv TM1 cotton RNA-seq, 12 h treated with salt
	SRX849545	Gossypium hirsutum cv TM1 cotton RNA-seq, 1 h treated with hot
	SRX849546	Gossypium hirsutum cv TM1 cotton RNA-seq, 3 h treated with hot
	SRX849547	Gossypium hirsutum cv TM1 cotton RNA-seq, 6 h treated with hot
	SRX849548	Gossypium hirsutum cv TM1 cotton RNA-seq, 12 h treated with hot
	SRX849541	Gossypium hirsutum cv TM1 cotton RNA-seq, 1 h treated with cold
	SRX849542	Gossypium hirsutum cv TM1 cotton RNA-seq, 3 h treated with cold
	SRX849543	Gossypium hirsutum cv TM1 cotton RNA-seq, 6 h treated with cold
	SRX849544	Gossypium hirsutum cv TM1 cotton RNA-seq, 12 h treated with cold
	SRX849557	Gossypium hirsutum cv TM1 cotton RNA-seq, 0 h CK
	SRX849558	Gossypium hirsutum cv TM1 cotton RNA-seq, 1 h CK
	SRX849559	Gossypium hirsutum cv TM1 cotton RNA-seq, 3 h CK
	SRX849560	Gossypium hirsutum cv TM1 cotton RNA-seq, 6 h CK
	SRX849561	Gossypium hirsutum cv TM1 cotton RNA-seq, 12 h CK
tissue/organ-specific expression pattern	SRX797899	Gossypium hirsutum cv TM1 cotton RNA-seq, root
	SRX797900	Gossypium hirsutum cv TM1 cotton RNA-seq, stem
	SRX797901	Gossypium hirsutum cv TM1 cotton RNA-seq, leaf
	SRX797902	Gossypium hirsutum cv TM1 cotton RNA-seq, torus
	SRX797903	Gossypium hirsutum cv TM1 cotton RNA-seq, petal
	SRX797904	Gossypium hirsutum cv TM1 cotton RNA-seq, stamen
	SRX797905	Gossypium hirsutum cv TM1 cotton RNA-seq, pistil
	SRX797906	Gossypium hirsutum cv TM1 cotton RNA-seq, calycle

Classification	Accession	Description
stage-specific expression pattern	SRX797886	Gossypium hirsutum cv TM1 cotton RNA-seq, 0h seed
	SRX797887	Gossypium hirsutum cv TM1 cotton RNA-seq, 5h seed
	SRX797888	Gossypium hirsutum cv TM1 cotton RNA-seq, 10h seed
	SRX797889	Gossypium hirsutum cv TM1 cotton RNA-seq, 24h cotyledon
	SRX797890	Gossypium hirsutum cv TM1 cotton RNA-seq, 48h cotyledon
	SRX797891	Gossypium hirsutum cv TM1 cotton RNA-seq, 72h cotyledon
	SRX797892	Gossypium hirsutum cv TM1 cotton RNA-seq, 96h cotyledon
	SRX797893	Gossypium hirsutum cv TM1 cotton RNA-seq, 120h cotyledon
	SRX797894	Gossypium hirsutum cv TM1 cotton RNA-seq, 24h root
	SRX797895	Gossypium hirsutum cv TM1 cotton RNA-seq, 48h root
	SRX797896	Gossypium hirsutum cv TM1 cotton RNA-seq, 72h root
	SRX797897	Gossypium hirsutum cv TM1 cotton RNA-seq, 96h root
	SRX797898	Gossypium hirsutum cv TM1 cotton RNA-seq, 120h root
	SRX797907	Gossypium hirsutum cv TM1 cotton RNA-seq, -3dpa ovule
	SRX797908	Gossypium hirsutum cv TM1 cotton RNA-seq, -1dpa ovule
	SRX797909	Gossypium hirsutum cv TM1 cotton RNA-seq, 0dpa ovule
	SRX797910	Gossypium hirsutum cv TM1 cotton RNA-seq, 1dpa ovule
	SRX797911	Gossypium hirsutum cv TM1 cotton RNA-seq, 3dpa ovule
	SRX797912	Gossypium hirsutum cv TM1 cotton RNA-seq, 5dpa ovule
	SRX797913	Gossypium hirsutum cv TM1 cotton RNA-seq, 10dpa ovule
	SRX797914	Gossypium hirsutum cv TM1 cotton RNA-seq, 20dpa ovule
	SRX797915	Gossypium hirsutum cv TM1 cotton RNA-seq, 25dpa ovule
	SRX797916	Gossypium hirsutum cv TM1 cotton RNA-seq, 35dpa ovule
	SRX797917	Gossypium hirsutum cv TM1 cotton RNA-seq, 5dpa fiber
	SRX797918	Gossypium hirsutum cv TM1 cotton RNA-seq, 10dpa fiber
	SRX797919	Gossypium hirsutum cv TM1 cotton RNA-seq, 20dpa fiber
	SRX797920	Gossypium hirsutum cv TM1 cotton RNA-seq, 25dpa fiber

These data that the study accession was SRP044705 were obtained from the NCBI Sequence Read Archive (SRA) (http://www.ncbi.nlm.nih.gov/sra)
