# Supplementary material for: Genome-wide characterization and expression analyses of superoxide dismutase (SOD) genes in Gossypium hirsutum
Source: BMC Genomics. 2017 May 12;18:376. doi: 10.1186/s12864-017-3768-5 (PMC5429560; doi:10.1186/s12864-017-3768-5)
Supplement: Supplementary file 4 — Gene-specific primers used for quantitative real-time PCR. (PDF 3302 kb) [file 12864_2017_3768_MOESM4_ESM.pdf]

Additional file 4: Table S8. Gene-specific primers used for quantitative real-time PCR.

Gene name		Primer (5'-3')	Length	Tm /℃	Amplicon /bp
GhUBQ7	Forward	AGAGGTCGAGTCTTCGGACA	20	60.0	102
	Reverse	GCTTGATCTTCTTGGGCTTG	19		
Gh_A13G0817	Forward	ATGGCTCTGCGAGTTTC	17	60.0	90
	Reverse	CAGCAAGGACAACGACC	17		
Gh_D13G1062	Forward	ATGGCTCTGCGAGTTTCT	18	60.0	91
	Reverse	TCAGCAAGGACAACGACA	18		
Gh_A13G1450	Forward	TGGGGACACAACTAACGGGTA	21	60.0	129
	Reverse	CCATCAACACCAACAGTGACATTC	24		
Gh_D13G1747	Forward	GGGGACATAACTAACGGGTGC	21	60.0	89
	Reverse	TGGCGGTTCTCATCTTCG	18		
Gh_A09G2473	Forward	CTCCCTTGCTGCTACTATTC	20	60.0	142
	Reverse	ATTCACAGTTGTAAGACCATCAT	24		
Gh_D09G0858	Forward	GGAAGTTGAAGGCGTTG	17	60.0	167
	Reverse	TATTGTTAGGATTGAAATGTGG	22		
Gh_A05G0722	Forward	CTTACTCCAGGGCTTCACGG	20	60.0	157
	Reverse	TAGCAACTATGTTTCCCAGGTCAC	24		
Gh_D05G0857	Forward	TACTCCAGGGCTTCACGG	18	60.0	158
	Reverse	CAACTATGTTTCCCAGGTCAG	21		
Gh_A11G2096	Forward	CCACTTAGCGGAGAGCA	17	60.0	113
	Reverse	GCACCTTCATTCCCTGTC	18		
Gh_D11G2412	Forward	CCACTTAGCCGACAGCATT	19	60.0	127
	Reverse	CACATCCGACTCTTACACCG	20		
Gh_A10G1595	Forward	ACCTTGCCCCTATTCGTG	18	60.0	89
	Reverse	TGATTCCAATGAACCAAAGTGTG	23		
Gh_D10G1852	Forward	AAGAACCGTGCCCCTAT	17	60.0	92
	Reverse	GATTCCAATGAACCAAAGTGA	21		
Gh_A05G2383	Forward	AGCCTCGACATGGTTCTTTGGGTT	23	60.0	102
	Reverse	AGCCTTGTAAAGCAGCACCCTCA	22		
Gh_D05G2648	Forward	CTCCATAACGCTATCCAGAAAGG	23	60.0	139
	Reverse	GCTCACCTCCTCCTTCACTAATA	23		
Gh_A07G0392	Forward	GCCATCAGAGAAGGATAAAA	20	60.0	105
	Reverse	TGCTCCCAGACATCAATAGTA	21		
Gh_D07G0457	Forward	GCCATCAGAGAAGGCTAAAG	20	60.0	103
	Reverse	CTCCCAGACATCAATCGTG	19		
Gh_A13G0530	Forward	AGGTGTGGAACCGTGACTT	19	60.0	107
	Reverse	TGTGAATGACCCAAAATCC	19		
Gh_D13G0600	Forward	GCTCTGGCTGGGTTTGG	17	60.0	157
	Reverse	CTCGCTTTGTCGTTCTGC	18		
