# Supplementary material for: Genome-wide characterization and expression analyses of superoxide dismutase (SOD) genes in Gossypium hirsutum
Source: BMC Genomics. 2017 May 12;18:376. doi: 10.1186/s12864-017-3768-5 (PMC5429560; doi:10.1186/s12864-017-3768-5)
Supplement: Supplementary file 5 — The alignment of the genome sequence and mRNA sequence of GhCSD7 with GhCSD8 and its downstream 5000 bp. (PDF 3302 kb) [file 12864_2017_3768_MOESM5_ESM.pdf]

Additional file 5: Figure S1. The alignment of the genome sequence and mRNA sequence of *GhCSD7* (Gh_A09G2473) with *GhCSD8* (Gh_D09G0858) and its downstream 5000bp (D09: 33604208.. 33609208 -). The multiple alignment was obtained using ClustalW and conserved amino acids were shaded using Boxshade sever (v.3.21) (http://sourceforge.net/projects/boxshade/). Dashes (−) indicated gaps in the alignment. The rectangle filled blue represented the GhCSD7, the rectangle filled red represented the C2H2-type zinc finger protein.

```
Gh_D09G0858          1 TGGTCTTACTCCTGGGCCTCATGGCTTCCACCTGATGGCTGCCCATATTTTCATGACAAC
Gh_A09G2473_mRNA     1 ----------------------------------ATGGCTGCCCATATTTTCACGACAAC
Gh_A09G2473          1 TGGTCTTACTCCTGGGCCTCATGGCTTCCACCTAATGGCTGCCCATATTTTCACGACAAC

Gh_D09G0858         61 CCCATCCCACCTCGCTCTCTCTTTTCCATCCTCAACTAACCCTTCAAACCCACCGGTTCT
Gh_A09G2473_mRNA    27 CCCGTCCCACCTCGCTCTCTCTTTTCCATCCTCAACTAACCCTTCAAACCCACCGGTTCT
Gh_A09G2473         61 CCCGTCCCACCTCGCTCTCTCTTTTCCATCCTCAACTAACCCTTCAAACCCACCGGTTCT

Gh_D09G0858        121 TCTCTCCTCATTTCGTGGGGTCTCTCTTAAGCTCCCTCGTCAATCCCTCTCCCTTGCTGC
Gh_A09G2473_mRNA    87 TCTCTCCTCATTTCGTGGGGTCTCTCTTAAGCTCCCTCGTCAATCCCTCTCCCTTGCTGC
Gh_A09G2473        121 TCTCTCCTCATTTCGTGGGGTCTCTCTTAAGCTCCCTCGTCAATCCCTCTCCCTTGCTGC

Gh_D09G0858        181 CACTATTCCCAAGAAGCCCTTTTCTGTGTTTGCTGTTACCAAGAAAGCTGTCGCTGTTCT
Gh_A09G2473_mRNA   147 TACTATTCCCAAGAAGCCCTTTTCTGTGTTTGCTGTTACCAAGAAAGCTGTCGCTGTTCT
Gh_A09G2473        181 TACTATTCCCAAGAAGCCCTTTTCTGTGTTTGCTGTTACCAAGAAAGCTGTCGCTGTTCT

Gh_D09G0858        241 TAAAGGCAATTCGGAAGTTGAAGGCGTTGTCACGTTGACCCAGGAAACTGATGGTTGACC
Gh_A09G2473_mRNA   207 TAAAGGTAATTCGGAAGTTGAAGGCGTTGTCACGTTGACCCAGGAAAATGATGGTCCG--
Gh_A09G2473        241 TAAAGGTAATTCGGAAGTTGAAGGCGTTGTCACGTTGACCCAGGAAAATGATGGGGAA--

Gh_D09G0858        301 CAGGAAACTGATGGTTTCTGTTTTTGCCCCCCCCCCCTCTTTTTATCATTTTATTTACTT
Gh_A09G2473_mRNA   265 ------------------TGTTTTTGCCCCCC-----TCTTTTCATCATTTTATTTACTT
Gh_A09G2473        299 -------------------GGGGTTGATATGC-----AAGCTCAAGCTGTATTTGTGGGG

Gh_D09G0858        361 TCTAATGCATTTTGTGATTTGGGTTTTTTTT-CTTCTAGTGGGGAAATTTTGATTCTTTG
Gh_A09G2473_mRNA   302 TCTAATGCATTTTGTGATTTGGGTTTTTTTTTCTTCTAGTGGGGAAATTTTGATTCTTTG
Gh_A09G2473        335 TCTGAT---CATCATCATCAAGAAGCCTTGATCATGAAAGGCAAGCGTACCAAGCGCCAA

Gh_D09G0858        420 GGATTTCCTTGTTTGTTTTTGAAGGATAAGAAGCCAAAGAAATGAAATGGGTACATTTTG
Gh_A09G2473_mRNA   362 GGATTTCCTTGTTTGTTTTAGAAGGATAAGAAGCCAAAGAAATGAAATGGGTACATTTTG
Gh_A09G2473        392 AGATCGCCATCCCTGTTTGGTGTCGCGGTGACTTCGAGCTCATCAAATGG--------TG

Gh_D09G0858        480 CTTTTTGGGGTTTTTCTTATTGACGAATTTAATTCCAAGTTTGATACTTTAAGATTTGAT
Gh_A09G2473_mRNA   422 CTGTTTGGGGTTTTTCTTATTGACGAATTCAATTCCAAGTTTGATACTTTAAGATTTGAT
Gh_A09G2473        444 GTGATGGAG-------TGGTGGAGGAATATAACTCGATTTCTTCTCCGATGACATCCGAT

Gh_D09G0858        540 TGAATCTTGTACAATTAGGGTGTATTAATTTTTTAATTTTGATGCCCAGAACATATTTAA
Gh_A09G2473_mRNA   482 TGAATCTTGTACAATTAGGGTGTATTAATTTTTGAATTTTGATGCCCAGAACATATTTAA
Gh_A09G2473        497 -GAGATTTACGAGAGCACCGAGGAAGAAGAGGAGATGGCTAATTGCCTAATCATGTT---

Gh_D09G0858        600 GGAAATAAAGTCATTGTGGAACTGTACTTAACATGTTCTTTCTTGGCTGTTTAACATGGA
Gh_A09G2473_mRNA   542 GGAAATAAAGTCATTGTGGAACTGTACTTAACATGTTCTTTCTTTGCTGTTTAACATGGA
Gh_A09G2473        553 -GGCACAAAGTGATGGT-----CCCAAAAGGAGTACTGTTGAAGCGAAATTCAAGATAGC
```

```
Gh_D09G0858        660  AACTAA-ATGAAAAAATGTTAGCTTGTAGTTCTCTTATCTTTTTGGCAATCAAATGTTAG
Gh_A09G2473_mRNA   602  AACTAA-ATGAAAAAATGTTAGCTTGTAGTTCTCTTATCTTTTTGGCAATCAAATTTT-G
Gh_A09G2473        607  TACTAAGACAAACAAGGGTGAGCTT----------TATGTCCACGAGTGTAAAACTT---

Gh_D09G0858        719  TATCATCTGGGAATGCTTATTTGTTCTGTGGATGAATAGGTCCTACAACTGTGAATGTTC
Gh_A09G2473_mRNA   660  TATCATCTGGGAATGCTTATTTGTTCTGTGGATGAATAGGTCCTACAACTGTGAATGTTC
Gh_A09G2473        654  -GTAACC--GGTCTTTCCCTTCGTTCCAAGCACTAGGAGGCCATATGGCAAGCCACAAGA

Gh_D09G0858        779  GAATTACTGGTCTTACTCCTGGGCCTCATGGCTTCCACCTGGTGAGTGTTTTCGGCTTTA
Gh_A09G2473_mRNA   720  GAATTACTGGTCTTACTCCTGGGCCTCATGGCTTCCACCTAGTGAGTGTTTTCGGCTT-A
Gh_A09G2473        711  AGCCTAAAGGCGCCATTGCAGAGC----------------AGAAAAGACCACTAGTTT--

Gh_D09G0858        839  GCTGCTTGTTGTTAGTGGATTTGAGTGCATAAAAGTTCTCCGAACAATATGATTTTAATC
Gh_A09G2473_mRNA   779  GCTGCTTGTTGTTAGTGGATTTGAGCGCATAAAAGTTCTCCGAACAATATGATTTTAATC
Gh_A09G2473        753  --TGGCAGTTAATGATGCAGTC-ATTGCTTTACGAGTAAGCAACAAAAGGGTAATAAAA

Gh_D09G0858        899  TTCCTTTTCGTTTCTTGTGTAGCATGAGTATGGTGACACAACAAATGGGTGCATGTCAAC
Gh_A09G2473_mRNA   839  TTCCTTTTCGTTTCTTGTGTAGCATGAGTATGGTGACACAACGAATGGGTGCATGTCTAC
Gh_A09G2473        810  TTCACGAGTGCTCAATCTGTGGCTCGGAATTCACGTCCGGTCAAGCCCTCGGAGGTCACA

Gh_D09G0858        959  AGGTATAACTTAGTGCATTTGAATCAAATGACGTGCGTTCTTATTGCGTTTCAGTTCATG
Gh_A09G2473_mRNA   899  AGGTATAACTTAGTGCGTTTGAATCAAATGATGTGCGTTCTTATTGCGATTTAGGTCATG
Gh_A09G2473        870  TGAGAAGGCATAGGACAGCATCAGCAGCTGCTGCAAGCAACCAAGATG-------CCATG

Gh_D09G0858       1019  TATGCACAAGATCCTTATTTTTAAAATTTTAAATGACAGGACCACATTTCAATCCTAACA
Gh_A09G2473_mRNA   959  TATGTATAAGTTCTTTATTTTAAAATTTTTAAATGACAGGAGCACATTTCAATCCTAACA
Gh_A09G2473        923  AATGGCAATGAT----GACATCAAGCCTAGGAATATTA----CGCCTTTGGATCTTAATC

Gh_D09G0858       1079  ATATGACACATGGTGCTCCTGAGGATGAAGTACGCCATGCGGGTGACCTGGGAAACATAA
Gh_A09G2473_mRNA  1019  ATATGACACATGGTGCTCCTGAGGATGAAGTACGCCATGCGGGTGACCTGGGAAACATAA
Gh_A09G2473        975  TTCCTGCACCTGAAGAT---GATGTTAGAG-ATTCCATGTTCG-AATTTGGAACACC---

Gh_D09G0858       1139  TTGCTAATGCTGATGGTAATACATTCTTAATTTTTTTTTTTCCTTTTTTAAATTTTTCTGT
Gh_A09G2473_mRNA  1079  TTGCTAATGCTGATGGTAATACATTCTTAATTTTTTTTTTTCCTATTTTAAATTTGTCTAT
Gh_A09G2473       1027  ---CCAAC----AAGCTATTGTCTTCTCCACACCAGCTTTGGTAGATTGCCATTA-CTAA

Gh_D09G0858       1199  GCACCAATTAACTTTATTCATTGCCTATGTTTGGTAATGCAGGAGTGGCAGAGGCAACAA
Gh_A09G2473_mRNA  1139  GCACCAATTAACTTTATTCATTGCCTATGTCTGGTAATGCAGGAGTGGCAGAGGCAACAA
Gh_A09G2473       1079  GTGGACATGAACTTAGTCTAACCACTGGGAATGCTGGCGGAAGATTGGCATGTGATACCA

Gh_D09G0858       1259  TTGTG-GACAATCAGGTGATGTTTAATAACTCGTGTTTCTTTCTGTGCAAATCTCCGTGC
Gh_A09G2473_mRNA  1199  TTGTG-GACAATCAGGTGATGTTCAATAACTCGTGTTTCTTTCTGTGCAAATCTCTGTGC
Gh_A09G2473       1139  CTAAGTGGCCCCAATGCGGTTGTTGGAAGAGCATTTGTGGTTC---ATGAGCTTGAGGAT

Gh_D09G0858       1318  ATTCATTT-CTCTTATGTGTCTGCATGAATAATTTCCCTTTTCAATGTGATCCTGTATAC
Gh_A09G2473_mRNA  1258  ATTCATTTTCTCTTATGTGTCTGCATGAATAATTTCCCTTTTCAATGTGATCCAGTATAC
Gh_A09G2473       1196  GATC-TTGGAAAGGGAGTGGCAGAGGCAACAATTGT--------GGACAATCAGGAGCAC

Gh_D09G0858       1377  TTTCTCAGTTGCTAAACTATGACTCTCTTGTGCAGATACCACTAAGTGGCCCCAATGCGG
Gh_A09G2473_mRNA  1318  TTTCTCAGTTGCTAAACTATGACTCTCTTGTGCAGATACCACTAAGTGGCCCCAATGCGG
Gh_A09G2473       1247  ATT-TCAATCCTAACAATATGACACA-TGGTGCTCCTGAGGATGAAGTACGCCA-TGCGG
```

```
Gh_D09G0858      1437 TTG---TTGGAAGAGCATTTGTGGTTCATGA-GCTTGAGGATGATCTTGGAAAGGGTAAT
Gh_A09G2473_mRNA 1378 TTG---TTGGAAGAGCATTTGTGGTTCATGA-GCTTGAGGATGATCTTGGAAAGGGTAAT
Gh_A09G2473      1304 GTGACCTGGGAAACATAATTGCTAATGCTGATGCATGAGTATGGTGACACAACGAATGGG

Gh_D09G0858      1493 TATTTGGCTTGCAATTCTAGCAGCTGCTTTAGTAGGAAACTTACCTCAAAAGTTGCAGAG
Gh_A09G2473_mRNA 1434 TATTTGGCTTGCAATTCTAGCAGTTGCTTTAGTAGGAAACTTCCCTCAAAAGTTGCAGAG
Gh_A09G2473      1364 TGCATGTCT-ACAGGTCCTACAACTGTGAATGTTCGAATTACTGGTCTTACTCCTGGGCC

Gh_D09G0858      1553 GGAAGAGTGATGTG-AGTGGCTATTTACTTCTTAGTTTTATTTTTCTTATATTTTTGTTT
Gh_A09G2473_mRNA 1494 GGAAGAGTGACGTGTAGTGGCTATTTACTTCTTAGTTTTATTTTTCTTATATTTTTGTTT
Gh_A09G2473      1423 TCATGGCTTCCACCTAATGGCTGCCCATATTTTCACGACAACCCCGTCCCACCTC-----

Gh_D09G0858      1612 GGTCATGCATTTGCAGGTGGACATGAACTTAGTCTAACCACTGGGAATGCTGGCGGAAGA
Gh_A09G2473_mRNA 1554 GGTCATGCATTTGCAGGTGGACATGAACTTAGTCTAACCACTGGGAATGCTGGCGGAAGA
Gh_A09G2473      1478 GCTCTCTCTTTTCCATCCTCA-ACTAACCCTTCAAACCCACCGGTTCTTCTCTCCTCATT

Gh_D09G0858      1672 TTGGCATGTGGTATGTACATGTGTACTTTTATCTACATGCT-------------------
Gh_A09G2473_mRNA 1614 TTGGCATGTGGTATGTACATGTGTACTTTTATCTACATGCTTATATGTTTCCAGAACAGA
Gh_A09G2473      1537 TCG---TGGGGTCTCT-CTTAAGCTCCCTCGTCAATCCCTC-------------------

Gh_D09G0858      1713 -------------ACATTTTCCAGACTCTCATCTCTGGTTTCCTTTTCTTGCATCTA-TG
Gh_A09G2473_mRNA 1674 CATGAAATTCATTACATTTTCCAGACTC--ATCTCTGGTTGC-TTTTCTTGCATCTA-TG
Gh_A09G2473      1574 --------------TCCCTTGCTGCTACTATTCCCAAGAAGCCCTTTTCTGTGTTTGCTG

Gh_D09G0858      1759 TTTTGAGCAGAATCATCTTTAG-CTTGATTATTATAGTTAAGCAAAAAGCACATTAGTAA
Gh_A09G2473_mRNA 1730 TTTTGAGCAGAATCATCTTTAG-CTTGATTATTATAGTTAAGCAAAAAGCACATTAGTAA
Gh_A09G2473      1620 TTACCAAGAAAGCTGTCGCTGTTCTTAAAGGTAATTCGGAAGTTGAAGGCG--TTGTCAC

Gh_D09G0858      1818 CTTGAAGAATGCTGCAGCTTGTTGTACCAGTTTTCTATAGCCTTCAGTTTGTTTGTTTGT
Gh_A09G2473_mRNA 1789 CTTGAAGAATGCTGCAGCTTGTTGTACCATTTTTCTATAGCCTTCAGTTTGTTTGTTTGT
Gh_A09G2473      1678 GTTGACCCAGGAAAATGATGGGGAAGGGGTTGATATGCAAGCTCAAGCT-GTATTTGTGG

Gh_D09G0858      1878 TTTTTGTTCACTTCATTGTCTCAGCTCAAGCCTTGATTTTGCTTGCGGGGATGATTCTGT
Gh_A09G2473_mRNA 1849 TTTTTGTTCACTTCATTGGCTCAGCTCAAGCCTTGAATTTGCTCACGGGGATGATTCTGT
Gh_A09G2473      1737 GGTCTGATCA--TCATCATCAAG----AAGCCTTGATCATGAAAGGCAAGCGTACCAAGC

Gh_D09G0858      1938 GTTTGAT----TCAAACTTTTCTCGAAACCTGACAAAGTTAAATCTTTAGTATATTAGAT
Gh_A09G2473_mRNA 1909 GTTTGAT----TCAAACTTTTCTCGAGACCTCACAAAATTAAATCTGTAGTATATTAGAT
Gh_A09G2473      1791 GCCAAAGATCGCCATCCCTGTTTGGTGTCGCGGTGACTTCGAGCTCATCAAATGGTGG--

Gh_D09G0858      1994 CCTTCAGCTTGATGCAATTCTATTCATTCATTTGTATGTATGTTTGGAGGGAGGGGATTT
Gh_A09G2473_mRNA 1965 CCTTCAGCTTGATGCAATTCTATTTATTCATTTGTATGTATGTTTGGAGGGAGGGGATTT
Gh_A09G2473      1849 -TGATGGAGTGGTGGAGGAATATAACTCGATTTCTTCTCCGATGACATCCGATGAGATTT

Gh_D09G0858      2054 AAAAGGCCATGGGTAGGACAAGGACAGATAGGTTTAAGGTTTTGATATATTTGCAGCCTC
Gh_A09G2473_mRNA 2025 AAAAGGCCATGGGTAGGACAGGGACAGATAGGTTTAAGGTTTCGATATATTTGCAGCCTC
Gh_A09G2473      1908 ACGAGAGCACCGA-GGAAGAAGAGGAGAT-GGCTAATTGCCTAATCATGTTGGCAC----

Gh_D09G0858      2114 TTAAGACTGATAACCCATCTCAGATGGACCCTTGAACTG---TTTGAAATACTTG-TGCA
Gh_A09G2473_mRNA 2085 TTAGGACTGATAATCCATCTCAGATGGACCCTTGAACTG---TTTGAAATACTTG-TGCA
Gh_A09G2473      1962 ---AAAGTGATGGTCC-CAAAAGGAGTACTGTTGAAGCGAAATTCAAGATAGCTACTAAG
```

```
Gh_D09G0858        2170 TTGACTATGGCTTGGCAATGAACATGGTGGTGTACGTATCTATCTTCTTTTTGTTGGACA
Gh_A09G2473_mRNA   2141 TTGACTATGGCTTGGCAATGATCATGGTGGTGTACGTATCTATCTTCTTTTTGTTGGACA
Gh_A09G2473        2018 ACAAACAAGGGTGAGCTTTATGTCCACGAGTGTA-AAACTTGTAACCGGTCTTTC--CCT

Gh_D09G0858        2230 TAGTTTTGTCAATTCTGCGGTTCCGTTTGAGACGGACTAAGTTATCTTTAGTCCCAGATC
Gh_A09G2473_mRNA   2201 TAGTTTTGTCAATTCTGCTGTTCCGTTTGAGACGGACTAAGTTATCTTTAGTCACAGATC
Gh_A09G2473        2075 TCGTTCCA--AGCACTAGGAGGCCATATGGCAAGCCACAAGAAGCCTAAAGGCGCCATTG

Gh_D09G0858        2290 TTTGATGTCTATGCGAATGTTCTTTCCCACATAAACATCCTATTCATATCAAAGCTAGTC
Gh_A09G2473_mRNA   2261 TTTGATGTCTATGCGAATGTTCTTTCCCACATAAACATATTATTCATATCAAAGCTAGTC
Gh_A09G2473        2133 CAGAGCAGAAAAGACCACTAGTTTTGGCAGTTAATGATGC-AGTCATTGCTTTACGAGTA

Gh_D09G0858        2350 TACATCAAACATTATACTGAATTTGTAACTTCCTCTCATCTTCTTCTGATAATGTCTGTG
Gh_A09G2473_mRNA   2321 TACATCAAACATTATACTGAATTTGTAAGTTCCTCTCATCTTCTTCTGATAATGTCTGTG
Gh_A09G2473        2192 AGCAACAAAAAGGGTAATAAAATTCACGAGTGCTCA-ATCTGTGGCTCGGAATTCACGTC

Gh_D09G0858        2410 CAGGTGTTGTTGGCTTGACTCCGGTATAAATCTTATCGATGAAACCAGTTTGGTGTTGC-
Gh_A09G2473_mRNA   2381 CAGGTGTTGTTGGCTTGACTCCGGTATAAATCTTATCGATGAAACCAGTTTGGTGTTGT-
Gh_A09G2473        2251 C-GGTCAAGCCCTCGGAGGTCACATGAGAAGGCATAGGACAGCATCAGCA-GCTGCTGCA

Gh_D09G0858        2469 -GCTATTTGTTGGTTGGATTTGGCAGCTTCTCTTATAGAGCCGCATGTGATGTTGCTTTC
Gh_A09G2473_mRNA   2440 -GCTATTTGTTGGTTGGATTTGGCAGCTTCTTTTATAGAGCCGCATGTGATGTTGCTTTC
Gh_A09G2473        2309 AGCAACCAAGATGCCATGAATGGCAATGATGACATCAAGCCTAGGAATATTACGCCTTTG

Gh_D09G0858        2528 AATGTTGATAGCGTGTTATATGAGATTGTGCAGACAAGTATTTAGTCGAATAACATTTGT
Gh_A09G2473_mRNA   2499 AATGTTGATAGCGTGTTATATGAGATTGTGCAGACAAGTATTTAGTCGAATAACATTTGT
Gh_A09G2473        2369 GATCTTAAT--CTTCCTGCACCTGAAGATGATGTTAGAGATTCCAT--GTTCGAATTTGG

Gh_D09G0858        2588 AGACCTTTGTTATGTCATTATTATCAACCCAATAAAAAATTAGCACATGGCTTGTGAACC
Gh_A09G2473_mRNA   2559 AGACCTTTGTTATGTCATTATTCTCAACCCAATAAAAAATTAGCACATGGCTTGTGAACC
Gh_A09G2473        2425 AACACCCCAACAAGCTATTGTCTTC--TCCACACCAGCTTTGGTAGATTGCC-ATTACTA

Gh_D09G0858        2648 TGCAGACTGTAAAACTTGCCTTTTTGAGCTTATGAAATTTTATGGTGCTGCTGGTCATTA
Gh_A09G2473_mRNA   2619 TGTAGACTGTAAAACTTGCCTTTTTGAGCTTATGAAATTTTATGGTGCTGCTGGTCATCA
Gh_A09G2473        2482 AGTGGAC-ATGAACTTAGTCT-----AACCACTGGGA-------ATGCTGGCGGAAGATT

Gh_D09G0858        2708 GGAAATTATGATGCCTTGTTGTAGTCAGTTATATCTCTGCTTCTCCTGGGAGTTGAGATC
Gh_A09G2473_mRNA   2679 GGAAATTATGATGCCTTCTTGTAGTCAGTTATATCTCTGCTTCTCCTGGTTGTTGAGATC
Gh_A09G2473        2529 GGCA--TGTGATACC-------ACTAAGTGGCCCCAATGCGGTTGTTGGAAG--AGCATT

Gh_D09G0858        2768 TGTAATTTGGCCGACCAAACTCTCAATCCCTACCTGTGCCGGGTGGAAGTATAAAATTAC
Gh_A09G2473_mRNA   2739 TGTAATTTGGCCGACCAAACTCTCAATCCCTACCAGTGATGGGTGGAAGTATAAAATTGC
Gh_A09G2473        2578 TGTGGTTCA--TGAGCTTGAGGATGATCTTGGAAAGGGAGTGGCAGAGGCA-ACAATTGT

Gh_D09G0858        2828 ACAAAAATTATAATCATTAGTTATCTTATATAATATTATACTTAAGGAAGGGGGATTAGA
Gh_A09G2473_mRNA   2799 ACAAAAATTATAATCATTAGTTATCTTATATAATATTAGACTTCAGGAAGGGGGATTAGA
Gh_A09G2473        2635 G-GACAATCAGGAGCACATTTCAATCCTAACAATATGACACAT------GGTGCTCCTGA

Gh_D09G0858        2888 ATCTCTAGCAACTCCACCCCTTACCTCTCATAATGAGAAAAAAAAAAAAAGAGGTTTGCT
Gh_A09G2473_mRNA   2859 ATCTCTAGCAACTCCACCCCCTACCTCTCATAATGAGAAAAAAAAAA-----GGTTTGCA
Gh_A09G2473        2688 GGATGAAGTA------CGCCATGCGGGTGACC-TGGGAAACATAA---------TTGCTA
```

```
Gh_D09G0858       2948 TTGCTGACTAGCGAGATCGGATTGGCTAGATGAAGAATTGGTCATGGTATCGGTTCTAAA
Gh_A09G2473_mRNA  2914 ATGCTGACTAGCGAGATTGGATTGGCTGGAGGAAGAATTGGTCATGGTATCGGTTCTAAA
Gh_A09G2473       2732 ATGCTGATGCATGAGTATGG--TGACACAACGAATGGGTGCATGTC-TACAGGTCCTACA

Gh_D09G0858       3008 ----TAAAGGGTTGGAGTGGTTGACTTGTAAATATGTTCAAATTGATTAATCAGATCGAT
Gh_A09G2473_mRNA  2974 ----TAAAGGATTGGAGTGGTTGATTTGTGAATAAGTTCAAATTGATTAGTCGGAACGAT
Gh_A09G2473       2789 ACTGTGAATGTTCGAATTACTGGTCTTACTCCTGGGCCTCA-TGGCTTCCACCTAATGGC

Gh_D09G0858       3064 CAAATTAATTTTTTAAAAATATTTTTAAATTTTTTAATAATCTATTCATTTAAAGTAAAC
Gh_A09G2473_mRNA  3030 TAAATTGATTTTTTAAAAATATTTTTAAATTTTTTAATAATTTATTCATTTGAACTAAAC
Gh_A09G2473       2848 TG--CCCATATTTTCACGACAACCCCGTCCCACCTCGCTCTCTCTTT-TCCATCCTCAAC

Gh_D09G0858       3124 AAATTCATCAGATTAGTTGAACTGACTGAAAAGAAATAGTTTAATTGGTTCCACCACCGA
Gh_A09G2473_mRNA  3090 AAATTCATCAGAT--------------GAAAATAAATAGTTTAATTGGTTTCACCACCGA
Gh_A09G2473       2905 TAACCCTTCAAAC----------------------------CCACCGGTTCTTCTCTCCT

Gh_D09G0858       3184 CTTGCTTTGCTACCTCTCACAGCCTCTT--------------------------------
Gh_A09G2473_mRNA  3136 CTTGCTTTGCTACCTCTCACAGCCTGCTTTCGTGGTAAGAGTTCAACAACGGCAGGAAAC
Gh_A09G2473       2937 CATTTCGTGGGGTCTCTCTTAAGCTCCCTCGTCA--------------------------

Gh_D09G0858       3212 -----------GCGGCTGTGCGATGTTCTTACCTCCCACAAACTGACATCATTTATTTAT
Gh_A09G2473_mRNA  3196 GGCAAAGAAATGCGGCTGTGCCATGTTCTTACCTCCCACAAACCAACATCATTTATTTAT
Gh_A09G2473       2971 -----------ATCCCTCTCCCTTGCTGCTACTATTCCCAAG--AAGCCCTTTTCTGTGT

Gh_D09G0858       3261 TGATTAATTTCCTGTACATTCAACCTTCATTTTTTTTATCACAACTTTTTTTAATAAAAT
Gh_A09G2473_mRNA  3256 TGATTGATTGCCTGTACATTGAACCTTCATTTTTTTA--TACAACTTTTTTTAATAAAAT
Gh_A09G2473       3018 TTGCTG-TTACCAAGA----AAGCTGTCGCTGTTCTTA----AAGGTAATTCGGAAGTTG

Gh_D09G0858       3321 GGGACATA--ATATTCAATTGAGATAGAAAGTCTTTAAATCTTGAATTAATTTTTTTACG
Gh_A09G2473_mRNA  3314 GGGACATATAATATTCAATTGAGATAGAAAGTGTTTAAATCTTGAATTAATTTTTTTACC
Gh_A09G2473       3069 AAGGCGT----TGTCACGTTGACCCAGGAA-------AATGATGGGGAAG----------

Gh_D09G0858       3379 TAGTTTAATATCGCAATATATCTTATTATTTATTCAATATGTTGACTAATATTAATTTTT
Gh_A09G2473_mRNA  3374 TAGTTTAATATCGCAATATATCTTATTATTTATTCAATATGTTGACTAATATTAATTTTT
Gh_A09G2473       3108 -GGGTTGATAT-GCAAGCTCAAGCTGTATTTGTGGGGTCTGATCATCATCATCAAGAAGC

Gh_D09G0858       3439 GTTAAAATCATGGATCACAAATCCTATACCACACCAATTAATCAAAAAAACGAATC-TGT
Gh_A09G2473_mRNA  3434 GTTAAAATCATGGATCACAAATCCTTTACCACACCAATTAATCAAAAAAACGAATC-TGT
Gh_A09G2473       3166 CTTGA--TCATGAAAGGCAAG--------CGTACCAAGCGCCAAAGATCGCCATCCCTGT

Gh_D09G0858       3498 TGGGTGATGCATGAACCCTTCAGAAGGAGCTTCGTTGGAAACAATGAACTTTGCTGCGTA
Gh_A09G2473_mRNA  3493 TGGGTGATGCATGAACCCTTCAGAAGGAGCTTCGTTGGAAACAATGAACTTTGCTGCGTA
Gh_A09G2473       3216 TTGGTGTCGCGGTGACTTC-------GAGCT-CATC--AAATGGTG------GTGATGGA

Gh_D09G0858       3558 TTTGTGGATAAA-ATAAACTACTGCTTTTAACATTGTTATTTCAGAATCTTTCTTAGATT
Gh_A09G2473_mRNA  3553 TTTGTGGATAAA-ATAAACTACTGCTTTTAACATTGTTATTTCAGAATCTTTCTTAGATT
Gh_A09G2473       3260 GTGGTGGAGGAATATAACTCGATTTCTTCTCCGATGACATCCGATGAGATTTACGAGAGC

Gh_D09G0858       3617 TGCCAAAACCTGTCATGCATGCCACTTTGATCTCAACTGCTGTTGTTGTTGGCTCTTTTT
Gh_A09G2473_mRNA  3612 TGCCAAAACCTGTCATGCATGCCACTTTGATCTCAACTGCTGTTGTTGTTGGCTCTTTTT
Gh_A09G2473       3320 ACCGAGGAAGAAGAGGAGATGGCTAATTG--CCTAATCA-------TGTTGGCACAAAGT
```

```
Gh_D09G0858       3677 TATGGCTCGGGCAAAGCCAATGCTAAATTGCTACAGTGAAATGGCTAGTTTGGGAAACTA
Gh_A09G2473_mRNA  3672 TATGGCTCGGGGAAAGCCAATCCTAAATTGCTACAGTGAAATGGCTAGTTTGGGAAACTA
Gh_A09G2473       3371 GATGGTCCCAAAAGGAGTACTGTTGAAGCGAAATT-CAAGATAGCTACTAAGACAAACAA

Gh_D09G0858       3737 AGAACTGGGAACTTAAGCATAATGGAGTTGATATTTTAATCGTTTAGCATGCATACCAAG
Gh_A09G2473_mRNA  3732 AGAACTTGGAACTTAAGCATAATGGAGTTGATATTTTAATCGTTTAGCATGCATAACAAG
Gh_A09G2473       3430 GGG----------TGAGCTTTATG----------TCCACGAGTGTAAAACTTGTAACCGG

Gh_D09G0858       3797 ATTATAGACTTGTCAGCTTTTCATTGAAGAACCCTTTTTGTAGTATCACTTAATCAGTTG
Gh_A09G2473_mRNA  3792 ATTATAGACTTGTCAGCTTTTCATTCAAGAACCCTTTTTGTAGTATCACTTAATCAGTTG
Gh_A09G2473       3470 TCTTTCCCTTCGTT--CCAAGCACT-AGGAGGCCATATGGCAA-GCCAC--AAGAAGCCT

Gh_D09G0858       3857 ATTCTATGATGGCANCCCC-CCCCCCCCCGCCCAAAAAAAAATACCATACTTGATTTGAT
Gh_A09G2473_mRNA  3852 ATTCTATGATGGCATCTCAATGCGAGACCCCCCCCCCCCCNNNNNNNNNNNNNNNNNNNN
Gh_A09G2473       3524 A----AAGGCGCCATTGCAGAGCAGAAAAGACCACTAGTTTTGGCAGTTAATGATGCAGT

Gh_D09G0858       3916 CAAATTATGATGACAAAAGAGAAGGGGGGGAGCTGGCGTGAAAGTAACCAGAATCTATAT
Gh_A09G2473_mRNA  3912 NNNNNNNNNNNNNNNNNNGGGGGGGGGGGGAGCTGGCGTGAAAGTAACCAGAATCTAT--
Gh_A09G2473       3580 CATTGCTTTACGAGTAAGCAACAAAAAGGGTAATAAAATTCACGAGTGCTCAATCTGTGG

Gh_D09G0858       3976 GAGGGAACTCAAATT-GATTAGGGTGTGGTATTTTGCTTTAAGAAGACATGGAGTGAATG
Gh_A09G2473_mRNA  3970 GAGGGAACTCAAATT-GATTGGAGTGTGGTATTTTGCTTTAAGAACACATGGAGTGAATG
Gh_A09G2473       3640 CTCGGAATTCACGTCCGGTCAAGCCCTCGGAGGTCACAT-GAGAAGGCATAGGAC-AGCA

Gh_D09G0858       4035 AAGGTAGCATCCCATGCTTTTTAACGTGCAGTTCCTCATTCCTTTTGCTTTAATTTAATA
Gh_A09G2473_mRNA  4029 AAGGTAGCATCCCATGCTTTTTAACGTGCACTTCCTCATTCCTTTTGCTTTAATTTAATA
Gh_A09G2473       3698 TCAGCAGCTGCTGCAAGCAACCAAGATGCCATGAATGGCAATGATGACATCAAGCCTAGG

Gh_D09G0858       4095 ATAAGTATTGTTATTATATATGGAGTGACTTGGGTGGGTGTGTGTGAACTCTGAAACTCT
Gh_A09G2473_mRNA  4089 ATAAATATTGTTATTATATATGGAGTGACTTGGGTGGGTGTGTGTGAACTCTGAAACTCT
Gh_A09G2473       3758 AATATTAC-GCCTTTGGATCTTAATCTTCCTGCACC--TGAAGATGATGTTAGAGATTCC

Gh_D09G0858       4155 CTCTTGACGTTAAGAACAACTCAATTGAACTGTCCTAACTCCCATTTTTGCCCCTTTTCG
Gh_A09G2473_mRNA  4149 CTCTTGACGTTAAGAACAACTCAATTGAACTGTCCTAACTCCCATTTTTGCCCCTTTTCG
Gh_A09G2473       3815 ATGTTCGAATTTGGAACACCCCAAC-AAGCTATTGTCTTCTCCACACCAGCTTTGGTAGA

Gh_D09G0858       4215 CCACC-CTATCTTTTCCACCTAATGCTCAACTCATATCTTTACCTAC--ACTTATTGTAG
Gh_A09G2473_mRNA  4209 CCACC-CTATCTTTTCCACCTAATGCTCAACTCATATCTTTACCTAC--ACTTATTGTAG
Gh_A09G2473       3874 TTGCCATTACTAAGTGGACATGAACTTAGTCTAACCACTGGGAATGCTGGCGGAAGATTG

Gh_D09G0858       4272 TCCTATCATCTCCCTAAGATGCTTGTTAACCAACCCTAAATACGTAGTT-TATTTGATTG
Gh_A09G2473_mRNA  4266 TCCTATCATCTCCCTAAGATGCTTGTTAACCAATCCTAAATACATAGTT-TATTTGATTG
Gh_A09G2473       3934 GCATGTGATACCACTAAGTGGCCCCAATGCGGTTGTTGGAAGAGCATTTGTGGTTCATGA

Gh_D09G0858       4331 TCTTAAATAATTGTCCTAATTCTAAATCAAATATTCACTCCATTTCTTTGTAATTAGAGA
Gh_A09G2473_mRNA  4325 TCTTAAATAATTATCCTAATTCTAGACCAAATATTCACTCCATTTCTTTGTAATTAGAGA
Gh_A09G2473       3994 GCTTGAG-GATGATCTTGGAAAGGGAGTGG-CAGAGGCAACAATTGTGGACAATCAGG--

Gh_D09G0858       4391 AATACATAATTATACCAACAATAAAAAAATAACAATAAATCTCTGTGTCGAGTAACTCTT
Gh_A09G2473_mRNA  4385 AATACATAATTATACCAACAATAAATAAATAATAATAAATCTCTGTGTCGAGTAACTCTT
Gh_A09G2473       4050 AGCACATTTCAATCCTAACAATA--TGACACATGGTGCTCCTGAGGATGAAGTACGCCAT
```

```
Gh_D09G0858       4451 ACTCCTAAAAAACCCCTAAATT-TGCACACAAGGAATTAAGGGGGAGTAATTTTTTATGA
Gh_A09G2473_mRNA  4445 ACTACTAAGAAACCCCTAAATCACGCACACAAGGAATTAAGGGA-AGTAATTTTG-ATGA
Gh_A09G2473       4108 GC----GGGTGACCTGGGAAACATAATTGCTAATGCTGATGCATGAGTATGGTGACACAA

Gh_D09G0858       4510 TGAAAGTAAAAACTTTTCTTTCTACCAGTAGCTTTAAAAGTTGGAAAT---GGAAAGAAA
Gh_A09G2473_mRNA  4503 TGAAAGTAAAAACTTTTCTTTCTACTAGTAGCTTTAAAAGTTGGAAAT---GAAAAGAAA
Gh_A09G2473       4164 CGAATGGGTGCATGTCTACAGGTCCTA-CAACTGTGAATGTTCGAATTACTGGTCTTACT

Gh_D09G0858       4567 AAAGAAGTGGGTGTTTTATAAGCAACAATAGTTATACTATTCATGGGCGGCTTTTCTGAG
Gh_A09G2473_mRNA  4560 AAAGAAGTGGGTGTTTTATAAGCAACAATAGTTATACTATTCATGGGCGGCTTTTCTGAG
Gh_A09G2473       4223 CCTGGGCCTCATGGCTTCCACCTAATGGCTGCCCATATTTTCACGACAACCCCGTCCCAC

Gh_D09G0858       4627 TTTTGACTCCAAAAATAACTGTCACCCTTCTTGTCA--TTTACTATATATAAGAACCCCA
Gh_A09G2473_mRNA  4620 TTTTGACTCCAAAAATAACTGTCACCCTTCTTGTCA--TTCGCTATATATAAGAACCCCG
Gh_A09G2473       4283 CTCGCTCTCTCTTTTCCATCCTCAACTAACCCTTCAAACCCACCGGTTCTTCTCTCCTCA

Gh_D09G0858       4685 TTTC-------TCCATCTATGCTTCCTCCGTGATCATTTTTTGCTCTTTCTAGCTGTTAC
Gh_A09G2473_mRNA  4678 TTTC-------TCCATCTATGCTTCCTCCGTGATCATTTTTTGCTCTTTCTAGCTGTTAC
Gh_A09G2473       4343 TTTCGTGGGGTCTCTCTTAAGCTCCCTCGTCAATCCCTCTCCCTTGCTGCTA-CTATTCC

Gh_D09G0858       4738 TCATTTTCTTTTGGTTAGGTCACACTACTTGCTTGTTTGTTGAGTACAATTTTATGTCTT
Gh_A09G2473_mRNA  4731 TCATTTTCTTTTGGTTAGGTCACACTACTTGCTTGTTTGTTGAGTACAATCTTATGTCTT
Gh_A09G2473       4402 CAAGAAGCCCTTTTCTGTGTT-TGCTGTTACCAAGAAAGCTGTCGCTGTTCTTAAAGGTA

Gh_D09G0858       4798 TTCTAAGTCTTTCATACTATTTTTGACTGGGTTTTGG---TTTAGGGGAAGGGGTGGATA
Gh_A09G2473_mRNA  4791 TTCTAAGTCTTTCATACTATTTTTGACTGGCTTTTGG---TTTAGGGGAAGGGGTTGATA
Gh_A09G2473       4461 ATTCGGAAGTTGAAGGCGTTGTCACGTTGACCCAGGAAAATGATGGGGAAGGGGTTGATA

Gh_D09G0858       4855 TGCAAGCTCAAGCTGTATTTGTGGGGTCTGATCATCATCTTCATCAAGCCTTGATCATGA
Gh_A09G2473_mRNA  4848 TGCAAGCTCAAGCTGTATTTGTGGGGTCTGATCATCATCATCAAGAAGCCTTGATCATGA
Gh_A09G2473       4521 TGCAAGCTCAAGCTGTATTTGTGGGGTCTGATCATCATCATCAAGAAGCCTTGATCATGA

Gh_D09G0858       4915 AAGGCAAGCGTACCAAGCGCCAAAGATCGCCATCCCTGTTTGGTGTCGCGGTGACTTCGA
Gh_A09G2473_mRNA  4908 AAGGCAAGCGTACCAAGCGCCAAAGATCGCCATCCCTGTTTGGTGTCGCGGTGACTTCGA
Gh_A09G2473       4581 AAGGCAAGCGTACCAAGCGCCAAAGATCGCCATCCCTGTTTGGTGTCGCGGTGACTTCGA

Gh_D09G0858       4975 GCTCATCAAATGGTGGTGATGGAGTGGTGGAGGAATATAACTCGATTTCTTCTCCGACGA
Gh_A09G2473_mRNA  4968 GCTCATCAAATGGTGGTGATGGAGTGGTGGAGGAATATAACTCGATTTCTTCTCCGATGA
Gh_A09G2473       4641 GCTCATCAAATGGTGGTGATGGAGTGGTGGAGGAATATAACTCGATTTCTTCTCCGATGA

Gh_D09G0858       5035 CATCCGACGAGATTTACGGGAGCACCGAGGAAGAAGAGGAGATGGCTAATTGCCTAATCA
Gh_A09G2473_mRNA  5028 CATCCGATGAGATTTACGAGAGCACCGAGGAAGAAGAGGAGATGGCTAATTGCCTAATCA
Gh_A09G2473       4701 CATCCGATGAGATTTACGAGAGCACCGAGGAAGAAGAGGAGATGGCTAATTGCCTAATCA

Gh_D09G0858       5095 TGTTGGCACAAAGTGATGGTCCCAAAAGGAGTACTGTTGAAGCGAAATTCAAGATAGCTA
Gh_A09G2473_mRNA  5088 TGTTGGCACAAAGTGATGGTCCCAAAAGGAGTACTGTTGAAGCGAAATTCAAGATAGCTA
Gh_A09G2473       4761 TGTTGGCACAAAGTGATGGTCCCAAAAGGAGTACTGTTGAAGCGAAATTCAAGATAGCTA

Gh_D09G0858       5155 CTAAGACAAACAAGGGTGAGCTTTATGTCCACGAGTGTAAAACTTGTAACCGGTCTTTCC
Gh_A09G2473_mRNA  5148 CTAAGACAAACAAGGGTGAGCTTTATGTCCACGAGTGTAAAACTTGTAACCGGTCTTTCC
Gh_A09G2473       4821 CTAAGACAAACAAGGGTGAGCTTTATGTCCACGAGTGTAAAACTTGTAACCGGTCTTTCC
```

```
Gh_D09G0858       5215 CTTCGTTCCAAGCACTAGGAGGCCATATGGCAAGCCACAAGAAGCCTAAAGGCGCCATTG
Gh_A09G2473_mRNA  5208 CTTCGTTCCAAGCACTAGGAGGCCATATGGCAAGCCACAAGAAGCCTAAAGGCGCCATTG
Gh_A09G2473       4881 CTTCGTTCCAAGCACTAGGAGGCCATATGGCAAGCCACAAGAAGCCTAAAGGCGCCATTG

Gh_D09G0858       5275 CAGAGCAGAAAAGACCACTAGTTTTGGCAGTTAATGATGCAGTCATTGCTTTACGAGTAA
Gh_A09G2473_mRNA  5268 CAGAGCAGAAAAGACCACTAGTTTTGGCAGTTAATGATGCAGTCATTGCTTTACGAGTAA
Gh_A09G2473       4941 CAGAGCAGAAAAGACCACTAGTTTTGGCAGTTAATGATGCAGTCATTGCTTTACGAGTAA

Gh_D09G0858       5335 GCAACAAAAAGGGTAATAAAATTCACGAGTGCTCAATCTGTGGTTCGGAATTCACGTCCG
Gh_A09G2473_mRNA  5328 GCAACAAAAAGGGTAATAAAATTCACGAGTGCTCAATCTGTGGCTCGGAATTCACGTCCG
Gh_A09G2473       5001 GCAACAAAAAGGGTAATAAAATTCACGAGTGCTCAATCTGTGGCTCGGAATTCACGTCCG

Gh_D09G0858       5395 GTCAAGCCCTCGGAGGTCACATGAGAAGGCATAGGACAGCATCAGCAGCTGCTGCAAGCA
Gh_A09G2473_mRNA  5388 GTCAAGCCCTCGGAGGTCACATGAGAAGGCATAGGACAGCATCAGCAGCTGCTGCAAGCA
Gh_A09G2473       5061 GTCAAGCCCTCGGAGGTCACATGAGAAGGCATAGGACAGCATCAGCAGCTGCTGCAAGCA

Gh_D09G0858       5455 ACCAAGATGCCATGAATGGCGATGATGACATCAAGCCTAGGAATATTACGCCTTTGGATC
Gh_A09G2473_mRNA  5448 ACCAAGATGCCATGAATGGCAATGATGACATCAAGCCTAGGAATATTACGCCTTTGGATC
Gh_A09G2473       5121 ACCAAGATGCCATGAATGGCAATGATGACATCAAGCCTAGGAATATTACGCCTTTGGATC

Gh_D09G0858       5515 TTAATCTTCCTGCACCTGAAGATGATGTTAGAGATTCCATGTTCGAATTTGGAACACCCC
Gh_A09G2473_mRNA  5508 TTAATCTTCCTGCACCTGAAGATGATGTTAGAGATTCCATGTTCGAATTTGGAACACCCC
Gh_A09G2473       5181 TTAATCTTCCTGCACCTGAAGATGATGTTAGAGATTCCATGTTCGAATTTGGAACACCCC

Gh_D09G0858       5575 AACAAGCTATTGTCTTCTCCACACCAGCTTTGGTAGATTGCCATTACTAAACAAGGAAAA
Gh_A09G2473_mRNA  5568 AACAAGCTATTGTCTTCTCCACACCAGCTTTGGTAGATTGCCATTACTAA----------
Gh_A09G2473       5241 AACAAGCTATTGTCTTCTCCACACCAGCTTTGGTAGATTGCCATTACTAAGTGGACATGA

Gh_D09G0858       5635 TCCTGAACCAA
Gh_A09G2473_mRNA       -----------
Gh_A09G2473       5301 ACTTAGTCTAA
```
