# Supplementary material for: Genome-wide characterization and expression analyses of superoxide dismutase (SOD) genes in Gossypium hirsutum
Source: BMC Genomics. 2017 May 12;18:376. doi: 10.1186/s12864-017-3768-5 (PMC5429560; doi:10.1186/s12864-017-3768-5)
Supplement: Supplementary file 6 — Motif sequences of GhSOD proteins identified by MEME tools. (PDF 3302 kb) [file 12864_2017_3768_MOESM6_ESM.pdf]

Additional file 6: Table S2. Motif sequences of GhSOD proteins identified by MEME tools. Cu ion binding sites and Zn ion binding sites are in box and green box, respectively. The conserved metal-binding domain "DVWEHAYY" for Mn-SODs or Fe-SODs are in red box, and Cu/Zn-SOD signatures (GFH[VL]H[AE][LY]GDTTN and GNAGGR[VL][AG]CG) are underline. **Sod_Cu** (PF00080), copper/zinc superoxide dismutase; **Sod_Fe_C** (PF02777), iron/manganese superoxide dismutases, C-terminal domain; **Sod_Fe_N** (PF00081), iron/manganese superoxide dismutases, alpha-hairpin domain.

Motif number	Width (aa)	Consensus sequence	Pfam domain
**1**	62	[SE]G[VT]V[TF][FL]TQE[GT]DGPTTV[TN][GV][RN] ITGL[TK]PGLHGFH[VL]H[AE][LY]GDTTNGCMST GPHFNP[NA][GN][KM][TE]HGAPED[EV]	Sod_Cu (PF00080)
**2**	20	[VN]RHAGDLGN[IV][TI][AV][GN][AD]DG[VC]A[SE] [FA]	Sod_Cu (PF00080)
**3**	47	QIPLSGP[NH]S[IV]IGRA[VF]VVH[AE][DL][PE]DDL GKGGHELS[KL][TS]TGNAGGR[VL][AG]CGII	Sod_Cu (PF00080)
**4**	28	[TR][DH]FGS[LF]ESLI[QE]K[MF]N[AS][EA][GA]AA L[QF]GSGWVWL	Sod_Fe_N (PF00081)
**5**	40	TY[SE]LK[DA]PPY[DP]L[GN]ALEP[AH]MS[GR]E[IT] [ML][QE][LY]H[WH][QG]KHHRTYV[TE]N[YL]NK	Sod_Fe_C (PF02777)
**6**	33	L[VN][TP][NL][VG][WP][DN][LY][VF]PLL[GN] [IL]DVWEHAYYL[QS][YT][QK]N[VD]RPDYL[LI]	Sod_Fe_C (PF02777)

Significant motifs (p-value<1e-100) of more than 20 aa lengths are predicted by MEME earch.
