# Supplementary material for: Genome-wide characterization and expression analyses of superoxide dismutase (SOD) genes in Gossypium hirsutum
Source: BMC Genomics. 2017 May 12;18:376. doi: 10.1186/s12864-017-3768-5 (PMC5429560; doi:10.1186/s12864-017-3768-5)
Supplement: Supplementary file 7 — Conserved motifs analysis of GhSOD proteins. (PDF 3330 kb) [file 12864_2017_3768_MOESM7_ESM.pdf]

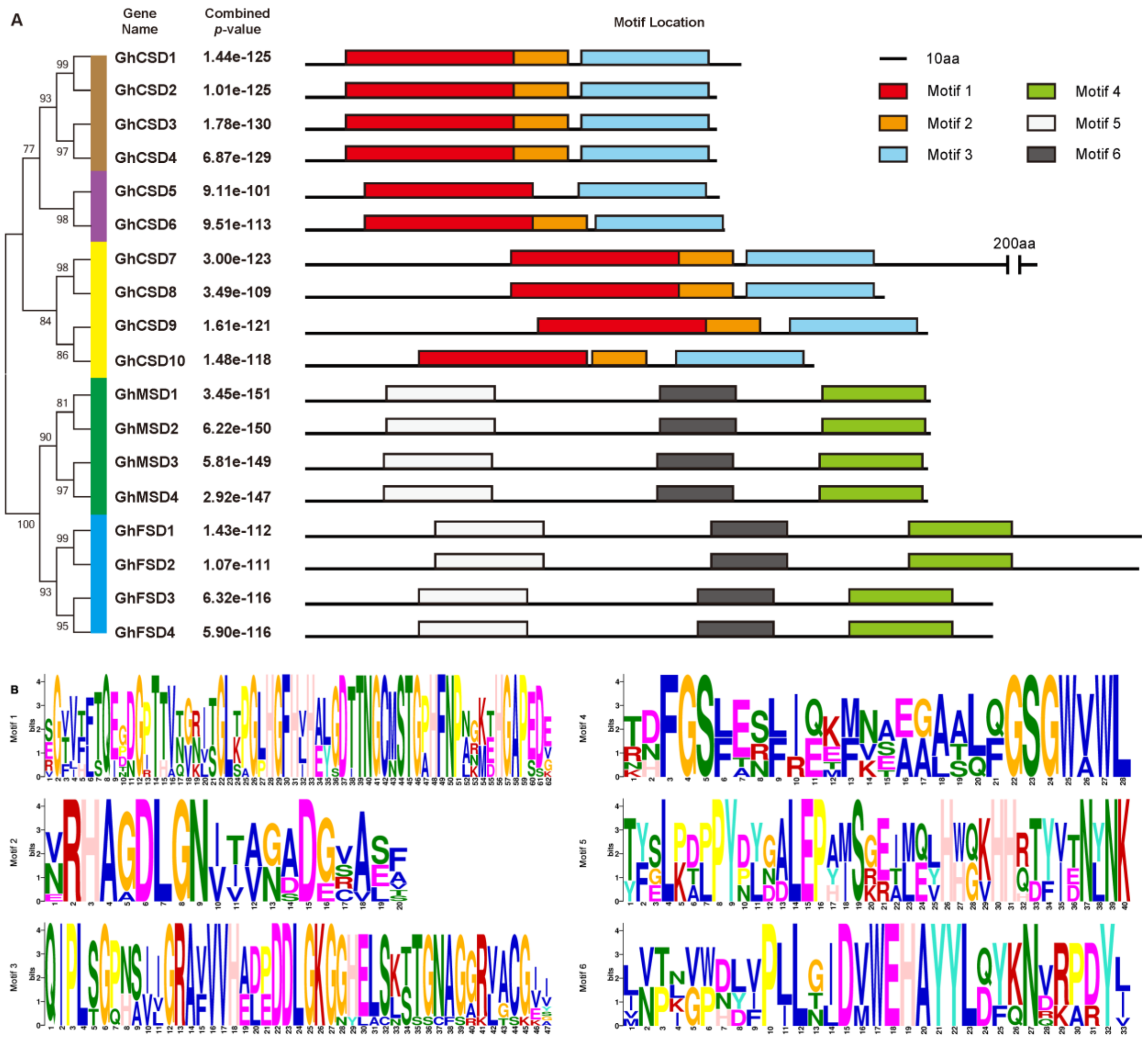

Additional file 7: Figure S2. Conserved motifs analysis of GhSOD proteins. (A) Clustering tree resulting from the amino acid alignments of GhSODs was shown on the left of the figure. The distribution of conserved motifs among the deduced GhSOD proteins was shown on the right of the figure. Different motif types were marked by different color blocks as indicated at the top right of the figure. Group Ia Ib and Ic are copper/zinc superoxide dismutases, Group Ia, Ib, Ic, IIa and IIb colored in brown, purple, yellow, green and blue, respectively; (B) The conserved motif logos in the GhSOD proteins.
