# Supplementary material for: Genome-wide characterization and expression analyses of superoxide dismutase (SOD) genes in Gossypium hirsutum
Source: BMC Genomics. 2017 May 12;18:376. doi: 10.1186/s12864-017-3768-5 (PMC5429560; doi:10.1186/s12864-017-3768-5)
Supplement: Supplementary file 8 — The details of sequences used in the phylogenetic relationship analysis. (PDF 3305 kb) [file 12864_2017_3768_MOESM8_ESM.pdf]

Additional file 8: Table S3. The details of sequences used in the phylogenetic relationship analysis.

Species	Gene name	Locus ID	Transcript name	Protein NCBI ID
Arabidopsis thaliana [1]	*AtCSD1*	AT1G08830	AT1G08830.1	NP_172360
	AtCSD2	AT2G28190	AT2G28190.1	NP_565666
	AtCSD3	AT5G18100	AT5G18100.1	NP_197311
	AtMSD1	AT3G10920	AT3G10920.1	NP_187703
	AtMSD2	AT3G56350	AT3G56350.1	NP_191194
	AtFSD1	AT4G25100	AT4G25100.1	NP_194240
	AtFSD2	AT5G51100	AT5G51100.1	NP_199923
	AtFSD3	AT5G23310	AT5G23310.1	NP_197722
Amborella trichopoda [2]	*AmtCSD1*	LOC18445195	XM_011629099.1	XP_011627401
	AmtCSD2	LOC18448821	XM_011630490.1	XP_011628792
	AmtCSD3	LOC18428583	XM_006837899.2	XP_006837962
	AmtMSD1	LOC18432270	XM_011624339.1	XP_011622641
	AmtFSD1	LOC18429084	XM_011623380.1	XP_011621682
	AmtFSD2	LOC18445372	XM_011629184.1	XP_011627486
Brachypodium distachyon [1]	*BdCSD1*	Bradi1g18340	Bradi1g18340.1	XP_003562484
	BdCSD2	Bradi1g69680	Bradi1g69680.1	XP_003558478
	BdCSD3	Bradi3g43070	Bradi3g43070.1	XP_003574947
	BdMSD1	Bradi2g30580	Bradi2g30580.1	XP_003568674
	BdFSD1	Bradi1g50550	Bradi1g50550.1	XP_003561161
	BdFSD2	Bradi1g51140	Bradi1g51140.1	XP_003557205
Chlamydomonas reinhardtii [2]	*CrMSD1*	MSD1	XM_001700006.1	XP_001700058
	CrMSD2	MSD2	XM_001699025.1	XP_001699077
	CrMSD3	MSD3	XM_001695895.1	XP_001695947
	CrMSD4	MSD4	XM_001690884.1	XP_001690936
	CrMSD5	MSD5	XM_001691696.1	XP_001691748
	CrFSD1	FSD1	XM_001690539.1	XP_001690591
Gossypium arboreum [3]	*GaCSD1*	Cotton_A_21978	FJ222550.1	ACI46676
	GaCSD2	Cotton_A_24238	JRRC01475755.1	KHG07500
	GaCSD3	Cotton_A_30467	JRRC01403715.1	KHG04013
	GaCSD4	Cotton_A_32487	EF123067.1	ABL63518
	GaCSD5	Cotton_A_36793	KN396107.1	KHG11568
	GaMSD1	Cotton_A_04050	JRRC01430423.1	KHG05444
	GaMSD2	Cotton_A_21263	KN395990.1	KHG11516
	GaFSD1	Cotton_A_03623	KN429884.1	KHG24725
	GaFSD2	Cotton_A_26478	KN444093.1	KHG28218

Gossypium raimondii [2]	*GrCSD1*	LOC105782588	XM_012607416.1	XP_012462870
			XM_012607417.1	XP_012462871
			XM_012607418.1	XP_012462872
	GrCSD2	LOC105783575	XM_012609115.1	XP_012464569
	GrCSD3	LOC105804461	XM_012637105.1	XP_012492559
			XM_012637104.1	XP_012492558
	GrCSD4	LOC105799144	XM_012629546.1	XP_012485000
	GrCSD5	LOC105768981	XM_012589298.1	XP_012444752
	GrMSD1	LOC105770420	XM_012591617.1	XP_012447071
	GrMSD2	LOC105777435	XM_012600706.1	XP_012456160
	GrFSD1	LOC105785559	XM_012611674.1	XP_012467128
			XM_012611682.1	XP_012467136
			XM_012611667.1	XP_012467121
	GrFSD2	LOC105782623	XM_012607482.1	XP_012462936
			XM_012607481.1	XP_012462935
			XM_012607480.1	XP_012462934
Glycine max [1]	*GmCSD1*	Glyma.19G240400	Glyma.19G240400.1	NP_001235298
	GmCSD2	Glyma.03G242900	Glyma.03G242900.1	NP_001235936
	GmCSD3	Glyma.16G153900	Glyma.16G153900.1	NP_001242811
	GmCSD4	Glyma.11G192700	Glyma.11G192700.1	XP_003538169
	GmCSD5	Glyma.12G081300	Glyma.12G081300.1	XP_003539793
	GmCSD6	Glyma.12G178800	Glyma.12G178800.1	NP_001238038
	GmMSD1	Glyma.06G144500	Glyma.06G144500.1	XP_003526813
	GmMSD2	Glyma.04G221300	Glyma.04G221300.1	NP_001235066
	GmFSD1	Glyma.02G087700	Glyma.02G087700.1	XP_003518624
	GmFSD2	Glyma.20G196900	Glyma.20G196900.1	NP_001238486
	GmFSD3	Glyma.10G193500	Glyma.10G193500.1	NP_001237901
	GmFSD4	Glyma.20G050800	Glyma.20G050800.1	XP_003555663
	GmFSD5	Glyma.10G117100	Glyma.10G117100.1	XP_003535933
Oryza sativa [1]	*OsCSD1*	LOC_Os03g22810	LOC_Os03g22810.1	ABF95937
	OsCSD2	LOC_Os03g11960	LOC_Os03g11960.1	ABF94673
	OsCSD3	LOC_Os07g46990	LOC_Os07g46990.1	P28757
	OsCSD4	LOC_Os08g44770	LOC_Os08g44770.1	P93407
	OsMSD1	LOC_Os05g25850	LOC_Os05g25850.1	Q43008
	OsFSD1	LOC_Os06g02500	LOC_Os06g02500.1	Q5VRL3
	OsFSD2	LOC_Os06g05110	LOC_Os06g05110.1	Q5VSB7

Physcomitrella patens [1]	*PpCSD1*	Pp3c24_4100	Pp3c24_4100.1	XP_001755582
	PpCSD2	Pp3c9_25690	Pp3c9_25690.1	XP_001770912
	PpCSD3	Pp3c9_24840	Pp3c9_24840.1	XP_001770943
	PpMSD1	Pp3c19_10240	Pp3c19_10240.1	XP_001774291
	PpFSD1	Pp3c7_19790	Pp3c7_19790.1	XP_001751975
	PpFSD2	Pp3c2_27440	Pp3c2_27440.1	XP_001756362
Populus trichocarpa [1]	*PtCSD1*	Potri.004G216700	Potri.004G216700.1	XP_006384962
	PtCSD2	Potri.009G005100	Potri.009G005100.1	XP_006378938
	PtCSD3	Potri.013G056900	Potri.013G056900.1	XP_002319157
	PtCSD4	Potri.019G035800	Potri.019G035800.1	XP_002325843
	PtCSD5	Potri.005G044400	Potri.005G044400.1	XP_006382702
	PtCSD6	Potri.013G031100	Potri.013G031100.1	XP_002319589
	PtMSD1	Potri.019G057300	Potri.019G057300.1	XP_002325924
	PtMSD2	Potri.013G092600	Potri.013G092600.1	XP_002319332
	PtFSD1	Potri.005G089600	Potri.005G089600.1	XP_002307154
	PtFSD2	Potri.012G112300	Potri.012G112300.1	XP_006376943
	PtFSD3	Potri.015G110400	Potri.015G110400.1	XP_002322315
Sorghum bicolor [1]	*SbCSD1*	Sobic.007G166600	Sobic.007G166600.1	XP_002445671
	SbCSD2	Sobic.001G453800	Sobic.001G453800.1	XP_002465634
	SbCSD3	Sobic.001G371900	Sobic.001G371900.1	
	SbCSD4	Sobic.002G407900	Sobic.002G407900.1	
	SbMSD1	Sobic.009G093200	Sobic.009G093200.1	XP_002439542
	SbFSD1	Sobic.010G012900	Sobic.010G012900.1	XP_002437717
	SbFSD2	Sobic.010G033000	Sobic.010G033000.1	XP_002436456

[1], protein sequences were retrieved from the Joint Genome Institute (JGI) (http://www.phytozome.net)

[2], protein sequences were retrieved from the NCBI protein database (http://www.ncbi.nlm.nih.gov/protein/)

[3], protein sequences were retrieved from CottonGen (https://www.cottongen.org/data/genome)
