# Supplementary material for: Genome-wide characterization and expression analyses of superoxide dismutase (SOD) genes in Gossypium hirsutum
Source: BMC Genomics. 2017 May 12;18:376. doi: 10.1186/s12864-017-3768-5 (PMC5429560; doi:10.1186/s12864-017-3768-5)
Supplement: Supplementary file 9 — Maximum-likelihood (ML) phylogenetic tree constructed for 70 SOD sequences from 9 flagship species and upland cotton. (PDF 3302 kb) [file 12864_2017_3768_MOESM9_ESM.pdf]

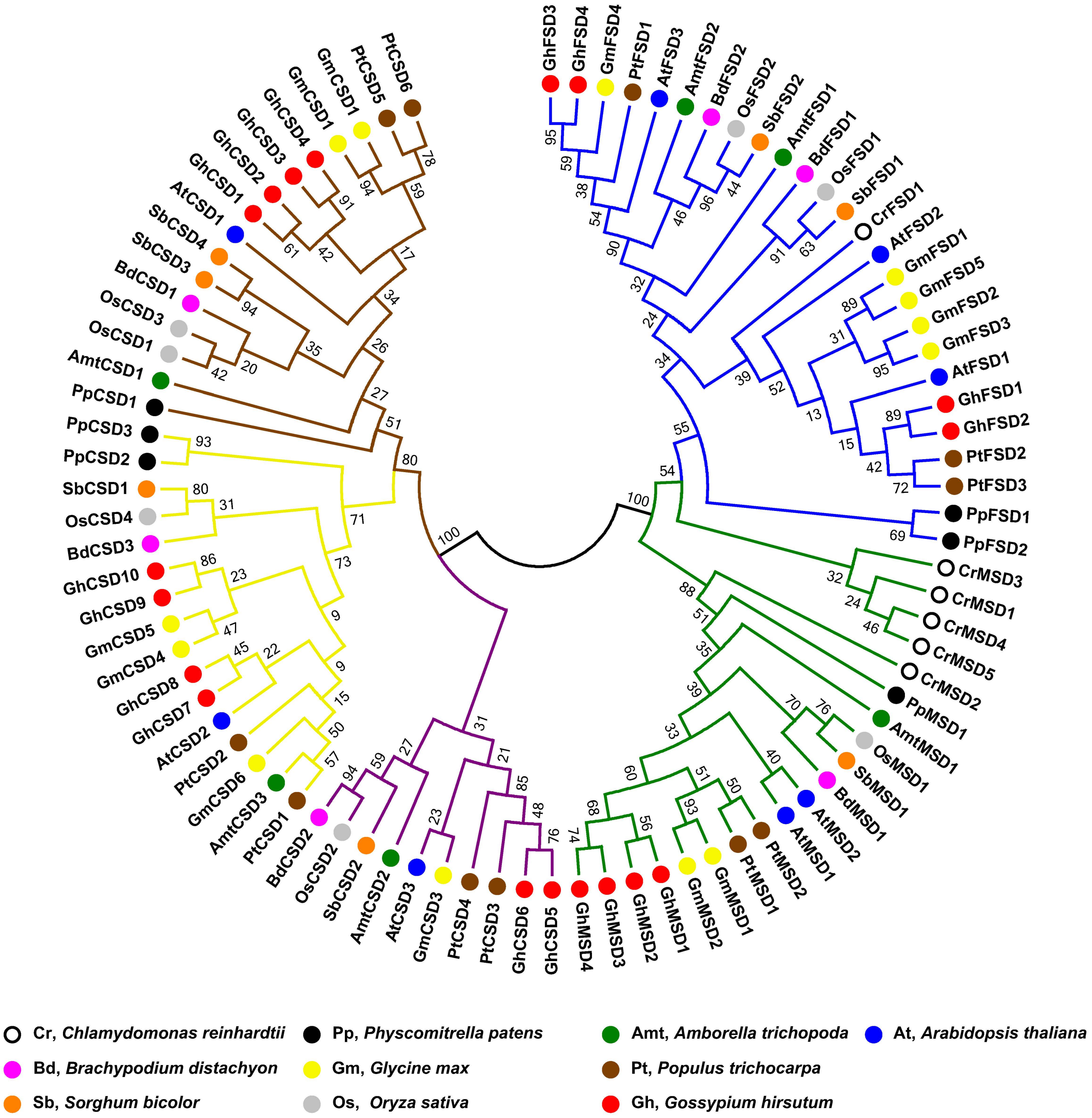

Additional file 9: Figure S3. Maximum-likelihood (ML) phylogenetic tree constructed for 70 *SOD* sequences from 9 flagship species and upland cotton. A maximum-likelihood tree was constructed using the MEGA6.0 program with 1000 bootstrap analyses using the full length amino acid sequences of 70 SODs from 9 flagship species (details as shown in **Additional file 6**). JTT+G was chosen as the most suitable substitution model based on the result of ModelGenerator before the phylogenetic tree reconstruction. The *SOD* genes were classified into two major groups and five subfamilies (groups Ia, Ib, Ic, IIa and IIb II, which branches were colored in brown, purple, yellow, green and blue, respectively). Bootstrap values are shown below nodes.
