# Supplementary material for: Genome-wide characterization and expression analyses of superoxide dismutase (SOD) genes in Gossypium hirsutum
Source: BMC Genomics. 2017 May 12;18:376. doi: 10.1186/s12864-017-3768-5 (PMC5429560; doi:10.1186/s12864-017-3768-5)
Supplement: Supplementary file 10 — Orthologous SOD gene pairs of G. hirsutum, G. arboreum, and G. raimondii. (PDF 3451 kb) [file 12864_2017_3768_MOESM10_ESM.pdf]

Additional file 10: Table S4. Orthologous *SOD* gene pairs of *G. hirsutum, G. arboreum, and G. raimondii* .

**Orthologous genes in *G. arboreum***		**Genes of At subgenome**		**Genes of Dt subgenome**		**Orthologous genes of *G. raimondii***	
**ID**	**Name**	**Name**	**ID**	**ID**	**Name**	**Name**	**ID**
Cotton_A_21978	***GaCSD1***	***GhCSD3***	Gh_A13G0817	Gh_D13G1062	***GhCSD4***	***GrCSD1***	Cotton_D_10024020
Cotton_A_24238	***GaCSD2***	***GhCSD1***	Gh_A13G1450	Gh_D11G2412	***GhCSD6***	***GrCSD3***	Cotton_D_10039927
Cotton_A_30467	***GaCSD3***	***GhCSD5***	Gh_A11G2096	Gh_D05G0857	***GhCSD10***	***GrCSD5***	Cotton_D_10006544
Cotton_A_32487	***GaCSD4***	***GhCSD7***	Gh_A09G2473	Gh_D05G2648	***GhMSD4***	***GrMSD1***	Cotton_D_10016246
Cotton_A_04050	***GaMSD1***	***GhMSD3***	Gh_A05G2383	Gh_D10G1852	***GhMSD2***	***GrMSD2***	Cotton_D_10018648
Cotton_A_21263	***GaMSD2***	***GhMSD1***	Gh_A10G1595	Gh_D07G0457	***GhFSD2***	***GrFSD1***	Cotton_D_10009356
Cotton_A_03623	***GaFSD1***	***GhFSD1***	Gh_A07G0392	Gh_D13G0600	***GhFSD4***	***GrFSD2***	Cotton_D_10030063
Cotton_A_26478	***GaFSD2***	***GhFSD3***	Gh_A13G0530				
