# Supplementary material for: Genome-wide characterization and expression analyses of superoxide dismutase (SOD) genes in Gossypium hirsutum
Source: BMC Genomics. 2017 May 12;18:376. doi: 10.1186/s12864-017-3768-5 (PMC5429560; doi:10.1186/s12864-017-3768-5)
Supplement: Supplementary file 11 — Gene Ontology annotations of 18 upland cotton SOD genes. (PDF 3304 kb) [file 12864_2017_3768_MOESM11_ESM.pdf]

**Additional file 11: Table S5.** Gene Ontology annotations of 18 upland cotton *SOD* genes.

| Gene name | Sequence ID | GO: Molecular function | GO: Biological process | GO: Cellular component |
|---|---|---|---|---|
| *GhCSD1* | Gh_A13G1450 | GO:0005507 : copper ion binding | GO:0042742 : defense response to bacterium | GO:0005737 : cytoplasm |
| | | GO:0005515 : protein binding | GO:0035195 : gene silencing by miRNA | GO:0005829 : cytosol |
| | | GO:0004784 : superoxide dismutase activity | GO:0055114 : oxidation-reduction process | GO:0005576 : extracellular region |
| | | GO:0008270 : zinc ion binding | GO:0019430 : removal of superoxide radicals | GO:0005634 : nucleus |
| | | | GO:0046688 : response to copper ion | |
| | | | GO:0010039 : response to iron ion | |
| | | | GO:0006979 : response to oxidative stress | |
| | | | GO:0010193 : response to ozone | |
| | | | GO:0009651 : response to salt stress | |
| | | | GO:0071484 : cellular response to light intensity | |
| | | | GO:0071493 : cellular response to UV-B | |
| | | | GO:0071329 : cellular response to sucrose stimulus | |
| *GhCSD2* | Gh_D13G1747 | GO:0005507 : copper ion binding | GO:0042742 : defense response to bacterium | GO:0005737 : cytoplasm |
| | | GO:0005515 : protein binding | GO:0035195 : gene silencing by miRNA | GO:0005829 : cytosol |
| | | GO:0004784 : superoxide dismutase activity | GO:0055114 : oxidation-reduction process | GO:0005576 : extracellular region |
| | | GO:0008270 : zinc ion binding | GO:0019430 : removal of superoxide radicals | GO:0005634 : nucleus |
| | | | GO:0046688 : response to copper ion | |
| | | | GO:0010039 : response to iron ion | |
| | | | GO:0006979 : response to oxidative stress | |
| | | | GO:0010193 : response to ozone | |
| | | | GO:0009651 : response to salt stress | |
| | | | GO:0071484 : cellular response to light intensity | |
| | | | GO:0071493 : cellular response to UV-B | |
| | | | GO:0071329 : cellular response to sucrose stimulus | |
| *GhCSD3* | Gh_A13G0817 | GO:0005507 : copper ion binding | GO:0042742 : defense response to bacterium | GO:0005737 : cytoplasm |
| | | GO:0005515 : protein binding | GO:0035195 : gene silencing by miRNA | GO:0005829 : cytosol |
| | | GO:0004784 : superoxide dismutase activity | GO:0055114 : oxidation-reduction process | GO:0005576 : extracellular region |
| | | GO:0008270 : zinc ion binding | GO:0019430 : removal of superoxide radicals | GO:0005634 : nucleus |
| | | | GO:0046688 : response to copper ion | |
| | | | GO:0010039 : response to iron ion | |
| | | | GO:0006979 : response to oxidative stress | |
| | | | GO:0010193 : response to ozone | |
| | | | GO:0009651 : response to salt stress | |
| | | | GO:0071484 : cellular response to light intensity | |
| | | | GO:0071493 : cellular response to UV-B | |
| | | | GO:0071329 : cellular response to sucrose stimulus | |

| | | | | |
|---|---|---|---|---|
| *GhCSD4* | Gh_D13G1062 | GO:0005507 : copper ion binding<br>GO:0005515 : protein binding<br>GO:0004784 : superoxide dismutase activity<br>GO:0008270 : zinc ion binding | GO:0042742 : defense response to bacterium<br>GO:0035195 : gene silencing by miRNA<br>GO:0055114 : oxidation-reduction process<br>GO:0019430 : removal of superoxide radicals<br>GO:0046688 : response to copper ion<br>GO:0010039 : response to iron ion<br>GO:0006979 : response to oxidative stress<br>GO:0010193 : response to ozone<br>GO:0009651 : response to salt stress<br>GO:0071484 : cellular response to light intensity<br>GO:0071493 : cellular response to UV-B<br>GO:0071329 : cellular response to sucrose stimulus | GO:0005737 : cytoplasm<br>GO:0005829 : cytosol<br>GO:0005576 : extracellular region<br>GO:0005634 : nucleus |
| *GhCSD5* | Gh_A11G2096 | GO:0005507 : copper ion binding<br>GO:0004784 : superoxide dismutase activity<br>GO:0008270 : zinc ion binding | GO:0055114 : oxidation-reduction process<br>GO:0019430 : removal of superoxide radicals<br>GO:0006979 : response to oxidative stress<br>GO:0010193 : response to ozone<br>GO:0009651 : response to salt stress<br>GO:0071484 : cellular response to light intensity<br>GO:0071493 : cellular response to UV-B | GO:0009507： chloroplast<br>GO:0005576 : extracellular region<br>GO:0005573 : vacuole<br>GO:0005577 : peroxisome |
| *GhCSD6* | Gh_D11G2412 | GO:0005507 : copper ion binding<br>GO:0004784 : superoxide dismutase activity<br>GO:0008270 : zinc ion binding | GO:0055114 : oxidation-reduction process<br>GO:0019430 : removal of superoxide radicals<br>GO:0006979 : response to oxidative stress<br>GO:0010193 : response to ozone<br>GO:0009651 : response to salt stress<br>GO:0071484 : cellular response to light intensity<br>GO:0071493 : cellular response to UV-B | GO:0009507： chloroplast<br>GO:0005576 : extracellular region<br>GO:0005573 : vacuole<br>GO:0005577 : peroxisome |
| *GhCSD7* | Gh_A09G2473 | GO:0005507 : copper ion binding<br>GO:0004784 : superoxide dismutase activity<br>GO:0008270 : zinc ion binding | GO:0035195 : gene silencing by miRNA<br>GO:0055114 : oxidation-reduction process<br>GO:0019430 : removal of superoxide radicals<br>GO:0046688 : response to copper ion<br>GO:0010039 : response to iron ion<br>GO:0006979 : response to oxidative stress<br>GO:0010193 : response to ozone<br>GO:0009651 : response to salt stress<br>GO:0071484 : cellular response to light intensity<br>GO:0071493 : cellular response to UV-B<br>GO:0071329 : cellular response to sucrose stimulus | GO:0009507： chloroplast<br>GO:0009579： thylakoid<br>GO:0048046： apoplast<br>GO:0009750： chloroplast stroma |

| | | | | |
|---|---|---|---|---|
| *GhCSD8* | Gh_D09G0858 | GO:0005507 : copper ion binding<br>GO:0004784 : superoxide dismutase activity<br>GO:0008270 : zinc ion binding | GO:0035195 : gene silencing by miRNA<br>GO:0055114 : oxidation-reduction process<br>GO:0019430 : removal of superoxide radicals<br>GO:0046688 : response to copper ion<br>GO:0010039 : response to iron ion<br>GO:0006979 : response to oxidative stress<br>GO:0010193 : response to ozone<br>GO:0009651 : response to salt stress<br>GO:0071484 : cellular response to light intensity<br>GO:0071493 : cellular response to UV-B<br>GO:0071329 : cellular response to sucrose stimulus | GO:0009507 ： chloroplast<br>GO:0009579 ： thylakoid<br>GO:0048046 ： apoplast<br>GO:0009750 ： chloroplast stroma |
| *GhCSD9* | Gh_A05G0722 | GO:0005507 : copper ion binding<br>GO:0004784 : superoxide dismutase activity<br>GO:0008270 : zinc ion binding | GO:0035195 : gene silencing by miRNA<br>GO:0055114 : oxidation-reduction process<br>GO:0019430 : removal of superoxide radicals<br>GO:0046688 : response to copper ion<br>GO:0010039 : response to iron ion<br>GO:0006979 : response to oxidative stress<br>GO:0010193 : response to ozone<br>GO:0009651 : response to salt stress<br>GO:0071484 : cellular response to light intensity<br>GO:0071493 : cellular response to UV-B<br>GO:0071329 : cellular response to sucrose stimulus | GO:0009507 ： chloroplast<br>GO:0009579 ： thylakoid<br>GO:0048046 ： apoplast<br>GO:0009750 ： chloroplast stroma |
| *GhCSD10* | Gh_D05G0857 | GO:0005507 : copper ion binding<br>GO:0004784 : superoxide dismutase activity<br>GO:0008270 : zinc ion binding | GO:0035195 : gene silencing by miRNA<br>GO:0055114 : oxidation-reduction process<br>GO:0019430 : removal of superoxide radicals<br>GO:0046688 : response to copper ion<br>GO:0010039 : response to iron ion<br>GO:0006979 : response to oxidative stress<br>GO:0010193 : response to ozone<br>GO:0009651 : response to salt stress<br>GO:0071484 : cellular response to light intensity<br>GO:0071493 : cellular response to UV-B<br>GO:0071329 : cellular response to sucrose stimulus | GO:0009507 ： chloroplast<br>GO:0009579 ： thylakoid<br>GO:0048046 ： apoplast<br>GO:0009750 ： chloroplast stroma |
| *GhFSD4* | Gh_D13G0600 | GO:0046872 : metal ion binding<br>GO:0005515 : protein binding<br>GO:0004784 : superoxide dismutase activity | GO:0055114 : oxidation-reduction process<br>GO:0019430 : removal of superoxide radicals | GO:0009507 : chloroplast<br>GO:0009295 : nucleoid<br>GO:0009579 : thylakoid<br>GO:0042646 : plastid nucleoid |

| | | | | |
|---|---|---|---|---|
| *GhMSD1* | Gh_A10G1595 | GO:0005507 : copper ion binding<br>GO:0046872 : metal ion binding<br>GO:0004784 : superoxide dismutase activity | GO:0042742 : defense response to bacterium<br>GO:0009793 : embryo development ending in seed dormancy<br>GO:0055114 : oxidation-reduction process<br>GO:0019430 : removal of superoxide radicals<br>GO:0009651 : response to salt stress<br>GO:0010043 : response to zinc ion | GO:0005739 : mitochondrion |
| *GhMSD2* | Gh_D10G1852 | GO:0005507 : copper ion binding<br>GO:0046872 : metal ion binding<br>GO:0004784 : superoxide dismutase activity | GO:0042742 : defense response to bacterium<br>GO:0009793 : embryo development ending in seed dormancy<br>GO:0055114 : oxidation-reduction process<br>GO:0019430 : removal of superoxide radicals<br>GO:0009651 : response to salt stress<br>GO:0010043 : response to zinc ion | GO:0005739 : mitochondrion |
| *GhMSD3* | Gh_A05G2383 | GO:0005507 : copper ion binding<br>GO:0046872 : metal ion binding<br>GO:0004784 : superoxide dismutase activity | GO:0042742 : defense response to bacterium<br>GO:0009793 : embryo development ending in seed dormancy<br>GO:0055114 : oxidation-reduction process<br>GO:0019430 : removal of superoxide radicals<br>GO:0009651 : response to salt stress<br>GO:0010043 : response to zinc ion | GO:0005739 : mitochondrion |
| *GhMSD4* | Gh_D05G2648 | GO:0005507 : copper ion binding<br>GO:0046872 : metal ion binding<br>GO:0004784 : superoxide dismutase activity | GO:0042742 : defense response to bacterium<br>GO:0009793 : embryo development ending in seed dormancy<br>GO:0055114 : oxidation-reduction process<br>GO:0019430 : removal of superoxide radicals<br>GO:0009651 : response to salt stress<br>GO:0010043 : response to zinc ion | GO:0005739 : mitochondrion |
| *GhFSD1* | Gh_A07G0392 | GO:0046872 : metal ion binding<br>GO:0005515 : protein binding<br>GO:0004784 : superoxide dismutase activity | GO:0055114 : oxidation-reduction process<br>GO:0009411 : response to UV | GO:0009507 : chloroplast<br>GO:0009295 : nucleoid<br>GO:0009579 : thylakoid |
| *GhFSD2* | Gh_D07G0457 | GO:0046872 : metal ion binding<br>GO:0005515 : protein binding<br>GO:0004784 : superoxide dismutase activity | GO:0055114 : oxidation-reduction process<br>GO:0009411 : response to UV | GO:0009507 : chloroplast<br>GO:0009295 : nucleoid<br>GO:0009579 : thylakoid |
| *GhFSD3* | Gh_A13G0530 | GO:0046872 : metal ion binding<br>GO:0005515 : protein binding<br>GO:0004784 : superoxide dismutase activity | GO:0055114 : oxidation-reduction process<br>GO:0019430 : removal of superoxide radicals | GO:0009507 : chloroplast<br>GO:0009295 : nucleoid<br>GO:0009579 : thylakoid<br>GO:0042646 : plastid nucleoid |
