# Supplementary material for: Genome-wide characterization and expression analyses of superoxide dismutase (SOD) genes in Gossypium hirsutum
Source: BMC Genomics. 2017 May 12;18:376. doi: 10.1186/s12864-017-3768-5 (PMC5429560; doi:10.1186/s12864-017-3768-5)
Supplement: Supplementary file 12 — Analysis of alternative splice in 4 GrSOD genes with at least two transcript variants. (PDF 3301 kb) [file 12864_2017_3768_MOESM12_ESM.pdf]

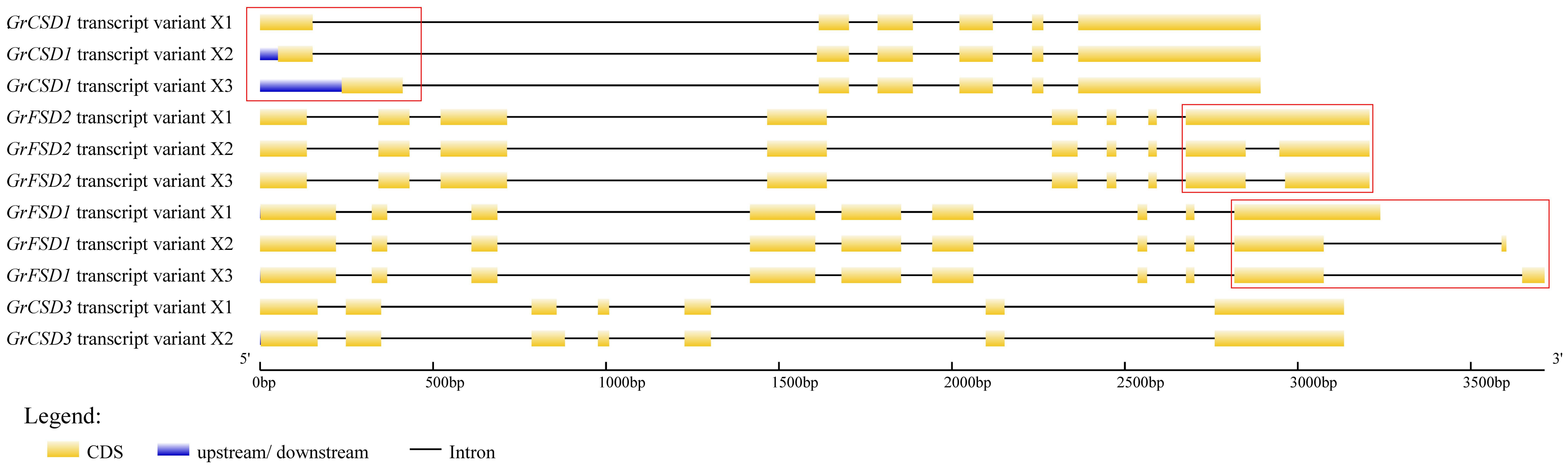

Additional file 12: Figure S4. Analysis of alternative splice in 4 *GrSOD* genes with at least two transcript variants. Rectangular frame colored red indicated the exons with differences.
