# Supplementary material for: Genome-wide characterization and expression analyses of superoxide dismutase (SOD) genes in Gossypium hirsutum
Source: BMC Genomics. 2017 May 12;18:376. doi: 10.1186/s12864-017-3768-5 (PMC5429560; doi:10.1186/s12864-017-3768-5)

Additional file 13

(**A**) The details of *CCS* genes in the genome of upland cotton.

Gene name	Sequence ID	Genomic position	Description
GhCCS1	Gh_A08G1589_NBI-AD1_v1.1	A08:94358411..94361302 -	copper chaperone for SOD1
GhCCS2	Gh_D08G1899_NBI-AD1_v1.1	D08:56882631..56885395 -	copper chaperone for SOD1

(**B**) The sites of *GhCCSs* targeted by ghr-miR398 and novel_mir_1205.

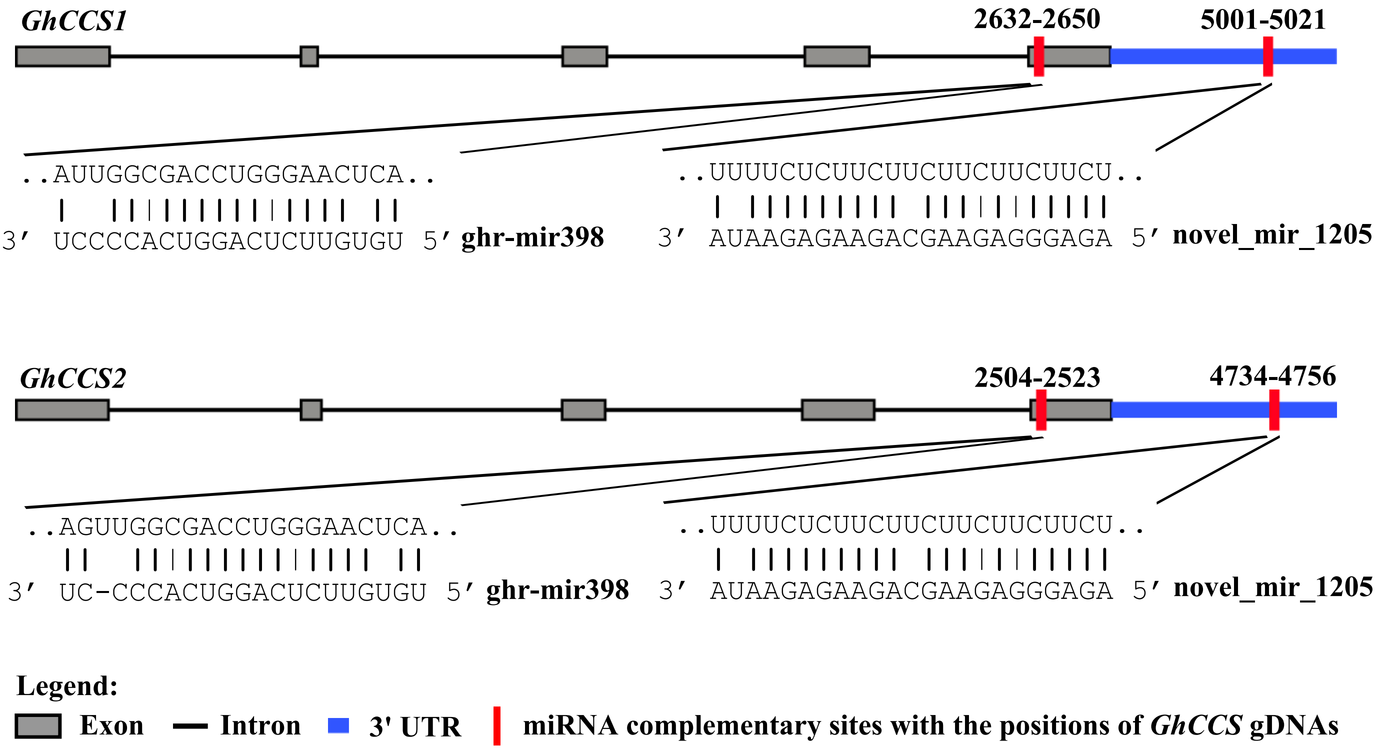

Supplement: Supplementary file 13 — The relevant information of upland cotton CCS genes. (PDF 3302 kb) [file 12864_2017_3768_MOESM13_ESM.pdf]
